# Molecular phylogeny of mega-diverse *Carabus* attests late Miocene evolution of alpine environments in the Himalayan–Tibetan Orogen

**DOI:** 10.1038/s41598-023-38999-6

**Published:** 2023-08-15

**Authors:** Joachim Schmidt, Lars Opgenoorth, Kangshan Mao, Chitra B. Baniya, Sylvia Hofmann

**Affiliations:** 1 General and Systematic Zoology, Institute of Biosciences, University of Rostock, 18055 Rostock, Germany; 2 Plant Ecology and Geobotany, Faculty of Biology, Philipps-University Marburg, 35043 Marburg, Germany; 3 College of Life Sciences, Sichuan University, Chengdu, 610065 China; 4 Central Department of Botany, Tribhuvan University, 44600 Kirtipur, Nepal; 5 Department Conservation Biology, UFZ–Helmholtz-Centre for Environmental Research GmbH, 04318 Leipzig, Germany; 6 Leibniz Institute for the Analysis of Biodiversity Change, Museum Koenig, 53113 Bonn, Germany

**Keywords:** Palaeontology, Phylogenetics, Speciation, Taxonomy, Evolution, Zoology, Entomology, Ecology, Biogeography, Palaeoecology

## Abstract

The timing, sequence, and scale of uplift of the Himalayan–Tibetan Orogen (HTO) are controversially debated. Many geoscientific studies assume paleoelevations close to present-day elevations and the existence of alpine environments across the HTO already in the late Paleogene, contradicting fossil data. Using molecular genetic data of ground beetles, we aim to reconstruct the paleoenvironmental history of the HTO, focusing on its southern margin (Himalayas, South Tibet). Based on a comprehensive sampling of extratropical *Carabus*, and ~ 10,000 bp of mitochondrial and nuclear DNA we applied Bayesian and Maximum likelihood methods to infer the phylogenetic relationships. We show that *Carabus* arrived in the HTO at the Oligocene–Miocene boundary. During the early Miocene, five lineages diversified in different parts of the HTO, initially in its southern center and on its eastern margin. Evolution of alpine taxa occurred during the late Miocene. There were apparently no habitats for *Carabus* before the late Oligocene. Until the Late Oligocene elevations must have been low throughout the HTO. Temperate forests emerged in South Tibet in the late Oligocene at the earliest. Alpine environments developed in the HTO from the late Miocene and, in large scale, during the Pliocene–Quaternary. Findings are consistent with fossil records but contrast with uplift models recovered from stable isotope paleoaltimetry.

## Introduction

**Figure 1:**
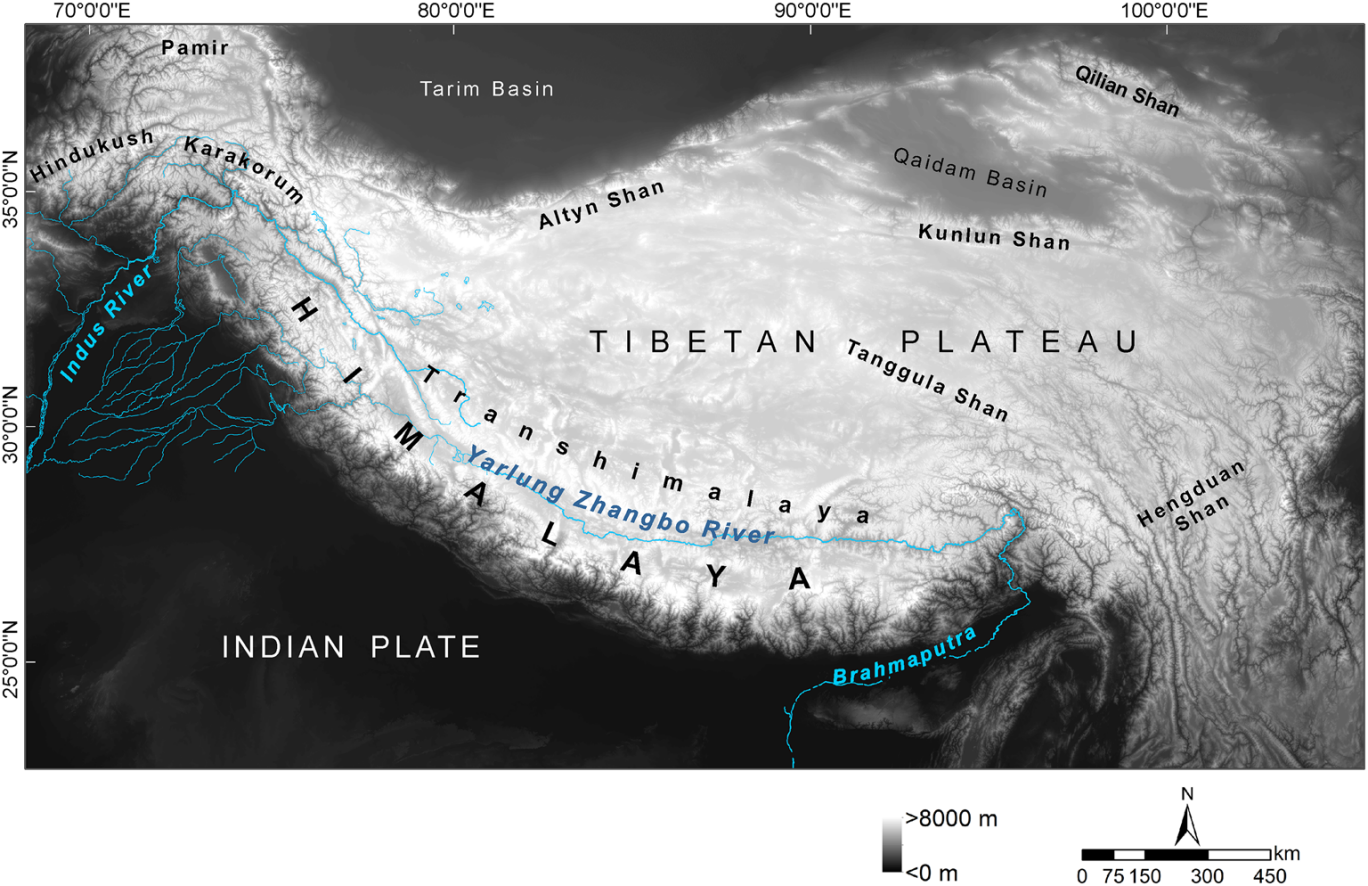
Overview map showing the main geological features of the Himalaya-Tibet orogeny. The Tibetan Himalaya (Northern Himalaya or Tethys Himalaya) and the Lesser Himalaya which are north and south, respectively, closely linked with the Greater Himalaya are not indicated; for a detailed geography of these geological unites see, e.g.,^3^.

With an extent of about 2.5 Mio km^2^ and an average elevation above 4000 m, the Himalayan–Tibetan orogenetic system (HTO) is the Earth's highest and largest mountain system (Fig. 1). It roughly encompasses the todays Tibetan Plateau and its deeply rugged eastern macro-slope which is built by the Hengduan Shan Mountains and the Three River Valleys (Mekong, Salween, Yangtze), the Qinghai Plateau with the Qilian Mountains, the Himalayas and the Karakorum. In biogeography, this orogenetic system is often referred to as the Tibetan Plateau or the Qinghai-Tibet-Plateau^1^. Here, we explicitly study evolutionary events in deep times and therefore, we avoid the term ‘plateau’ because it may potentially create a false impression on the paleotopography of that area^2^.

The Cenozoic topographic formation of the HTO is crucial for understanding the development of regional and global atmospheric circulation systems, local paleoenvironments, and the evolution of its mega-diverse biota^4–6^. However, the timing, sequence, and scale of surface uplift of the respective parts of the HTO are still in flux and controversially debated^2,7,8^. Several geoscientific studies present evidence for a high elevated Tibetan Plateau as early as the Eocene or even earlier (e.g.,^9–12^). Stable isotope paleoaltimetry estimates show local elevations from different parts of HTO close to modern values, corresponding to subalpine- alpine ecotones, by the middle Oligocene at the latest^13–16^. Other studies, e.g., based on tectonic data, sedimentation records, or fossil findings, suggest significant uplift of the HTO and the development of the respective paleoenvironments with the beginning of the Neogene at the earliest^17–23^. Several models for the topographic development of the orogenetic system were recently proposed, assuming the occurrence of very high elevated areas (≥ 4000 m a.s.l.) in the HTO during the Paleogene (e.g.,^2,8,12,24^). These models, however, differ significantly in their uplift scenarios for certain parts of the HTO. Most frequently, the quantity of enhanced elevation, considered in those models, is calculated from stable isotope paleoaltimetry^13–16^, a method that might be biased in different ways and that does not seem to be applicable to Eocene Asia^25^. Consequently, paleoelevations of the HTO derived by this method might be seriously overestimated^25^, implying alpine environments during the Paleogene.

The fossil record for the Cenozoic HTO is relatively rich and has significantly contributed to the ongoing discussion on the uplift history of the orogenic system^2,7^. However, insufficient age constraints of the deposits entail substantially different uplift scenarios^2,8^. Importantly, no fossil evidence exists for the occurrence of cold temperate and alpine environments in the southern and central HTO during the Paleogene, while tropical to warm temperate conditions prevailed in the area up to the early Miocene (Supporting Information Table S1). This general lack of fossil records from cold environments is particularly relevant with respect to the results from the stable isotope paleoaltimetry (see above).

Understanding the history of the spatio-temporal surface uplift on the southern HTO margin seems even more challenging. There is evidence that the Tibetan Himalaya (or Tethys Himalaya) and the Lesser Himalaya had a marine development until the Eocene (for overview see^12^). The Tibetan Himalaya interlocks closely with the Greater Himalaya on its northern side, and with the Lesser Himalaya on its southern side (for details of the highly complex Himalayan geology see^12^). The Himalayas probably raised rapidly in the early-mid Miocene, although most supporting data are gained from the Tibetan Himalaya (e.g.,^26–28^). For the Greater Himalaya, 5000 m and higher mean elevations, corresponding to alpine and nival regions are estimated and dated at about 15 Mya based, again, on stable isotope paleoaltimetry^29^. A more recent study suggests a likewise high elevated area stretching into today's Nepal during the Miocene^30^. In contrast, fossil data indicate the presence of cold temperate environments and a significant uplift of the Himalayas only at the Late Miocene or Pliocene^20,31^.

Any of the different scenarios for the topographic evolution of the central and southern parts of the HTO necessarily lead to different models of the local paleoenvironments, making biogeographic conclusions challenging or even impossible. Nevertheless, phylogeny offers an independent line of evidence for the positioning of major topographical features, which have been proved valid in refining the timing of events substantiated by geologic records^32^. Perhaps most noteworthy, there is no evidence from any phylogeographic study for the presence of cold temperate or alpine conditions in the Paleogene HTO (except for^33^, but see^34^, although potential misinterpretation of data may have distorted our understanding of the origin and historical biogeography of the terrestrial biota of the HTO^1,35^; see “Discussion”).

Here we aim to contribute to better understanding of the paleoenvironmental history of High Asia. We use extant flightless Himalayan ground beetles as a proxy for the topographic and climatic development of the HTO, focusing particularly on its southern margin. The bedrock of our study is a molecular data set of a comprehensive sampling of *Carabus*. Importantly, we included all species groups distributed in the Himalaya and on the Tibetan Plateau and all species of the most diverse Himalayan endemic *Carabus* subgenus *Meganebrius*. Due to their early evolutionary origin, decidedly limited dispersal ability, and strong climatic ties, cladogenesis within endemic lineages of these beetles can reflect early events as well as local differences in the spatio-temporal surface uplift history of the HTO^36^; for details see Supporting Information Text. We investigate specifically the striking peculiarities in their distribution using methods of molecular phylogeny. Based on the time frame in which montane-adapted and alpine species have evolved, spatially explicit inferences can be made about paleoenvironmental conditions across the HTO and thus its surface elevation.

## Results

### Phylogeny of *Carabus* from the Himalaya and Tibetan Plateau

**Figure 2:**
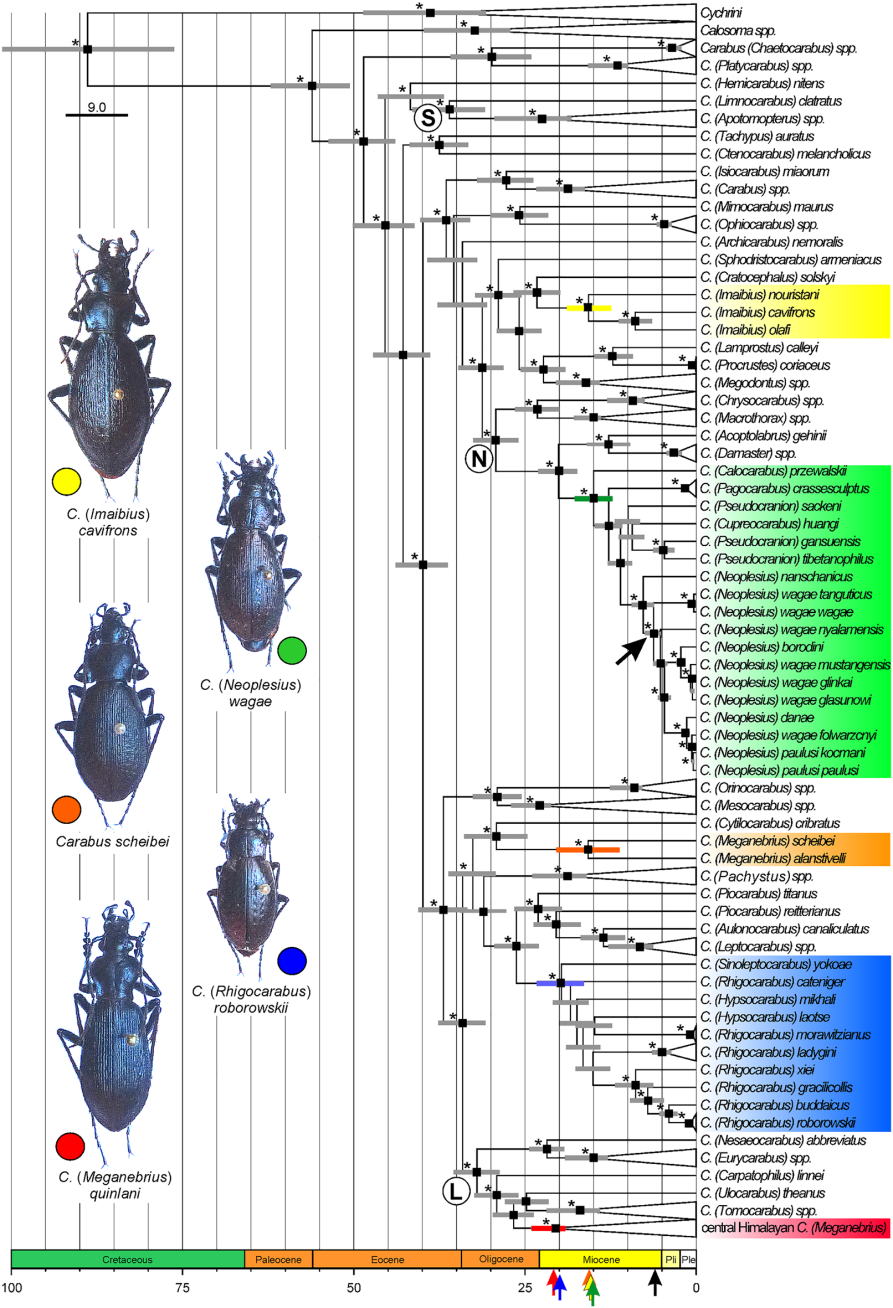
Ultrametric time-calibrated phylogeny of *Carabus* beetles and outgroups. The tree was generated with BEAST2 based on the concatenated sequence data. Lineages endemic to certain parts of the HTO are highlighted by different colours (photographs of representative species are inserted to the left). Black rectangles and stars at branch nodes refer to posterior probabilities ≥ 0.98 and bootstrap values > 70.0, respectively. Grey bars specify the 95% HPD of the respective node age (coloured bars highlight the 95% HPD for crown group ages of endemic lineages). Coloured arrows at the time axis point to the node ages of the crown groups of the respective endemic lineages; the black arrow points to the node age of southern Tibetan *Neoplesius*. Suprageneric taxa discussed in the text are marked by capitals: L: Latipalpi; N: Neocarabi; S: Spinulati. The subtree of the central Himalayan *Meganebrius* is shown in Fig. 3. For the uncollapsed tree inferred with MrBayes see Supporting Information Fig. S1.

From the multigene analyses, we inferred a well-resolved tree with strong support for a great majority of the clades (Fig. 2, Supporting Information Fig. S1). Main branching patterns are widely consistent with previous results^37^ with few exceptions: In our phylogenetic tree, the subgenus *Hemicarabus* clusters with the *Carabus* Spinulati group, and the subgenera *Tachypus* and *Ctenocarabus* form a well-supported clade.

The Himalayan subgenus *Meganebrius* is recovered polyphyletic with two well-supported monophyla (Fig. 2): all species from the Greater Himalaya of central and east Nepal together form a species-diverse clade (in the following referred to as 'central Himalayan *Meganebrius*'). This clade is sister to a clade formed by the Holarctic *Tomocarabus* and the Middle Asian *Ulocarabus*, which are nested in the Latipalpi group of *Carabus*^37^. Two species distributed in widely separated parts of the Greater Himalaya, namely *C. alanstivelli* from the Far West of Nepal and *C. scheibei* from the Kashmir-Himalaya, form a clade outside of Latipalpi which constitutes the sister group of the West Asian subgenus *Cytilocarabus* (in the following referred to as 'west Himalayan *Carabus scheibei* group'). The west Himalayan subgenus *Imaibius* forms the sister group of *Cratocephalus* from the Tian Shan and, consistent with previous results^37^, is closely related to the western Palearctic subgenera *Lamprostus*, *Megodontus*, and *Procrustes* (Fig. 2).

The subgenus *Neoplesius* from central and south Tibet clusters as part of an East Tibetan clade comprising the subgenera *Calocarabus*, *Pagocarabus*, *Pseudocranion* and *Cupreocarabus* (in the following called '*Pagocarabus* clade'). This clade together with the East Asian subgenera *Acoptolabrus* and *Damaster* form a well-supported clade within the Neocarabi group^37^ (Fig. 2). The taxon *Rhigocarabus* from east and central Tibet appears paraphyletic due to the east Tibetan *Hypsocarabus* that clusters within this group although with low support. However, both these subgenera together with east Tibetan *Sinoleptocarabus* form a well-supported clade (in the following called '*Rhigocarabus* clade') which is the sister group of a clade formed by East Asian species of the subgenera *Aulonocarabus*, *Leptocarabus*, *Pachystus*, and *Piocarabus* (Fig. 2).

### Molecular dating of endemic HTO species groups

**Figure 3:**
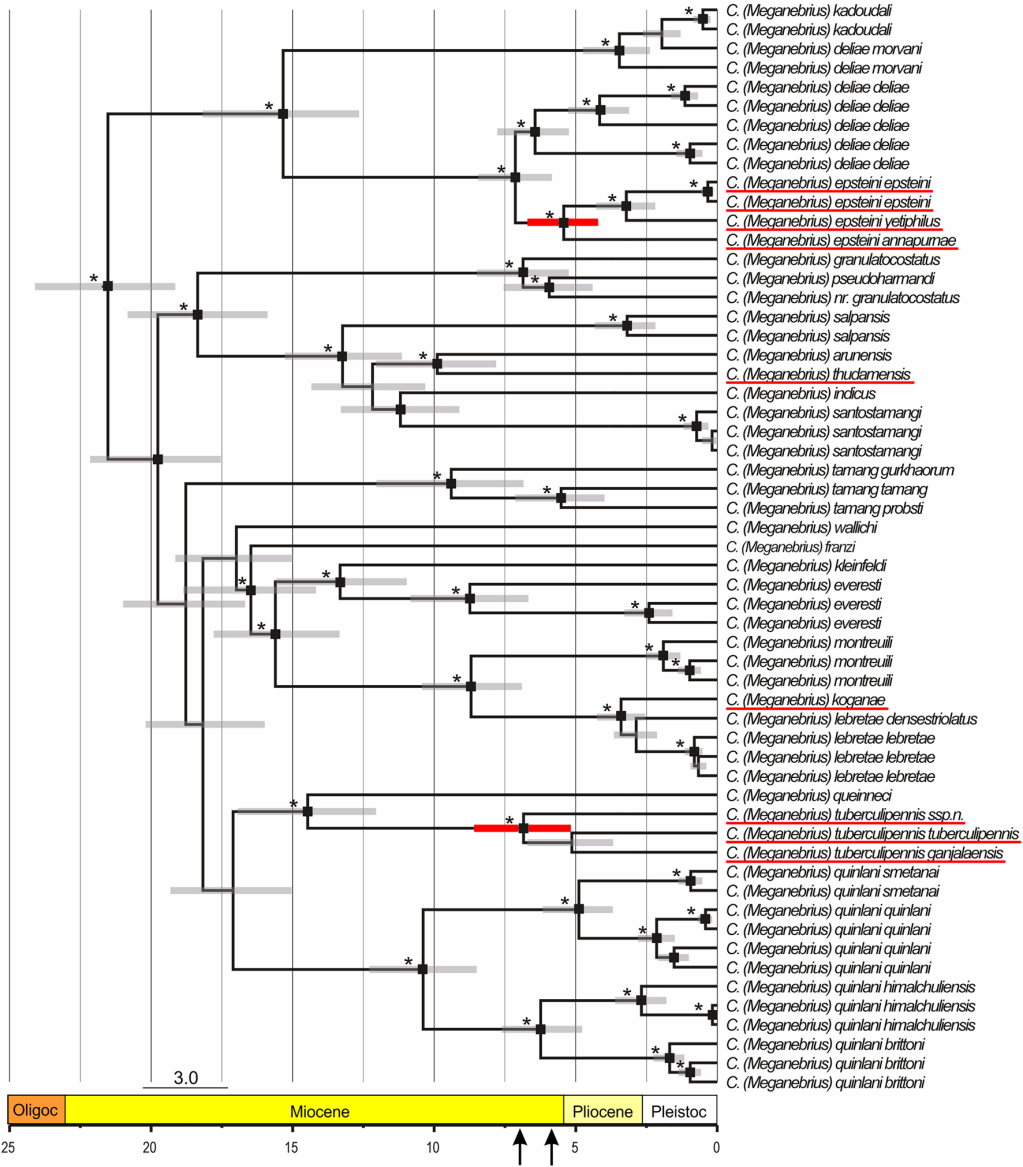
Subtree for *Meganebrius* of the time-calibrated phylogeny as shown in Fig. 2. Names of species and subspecies adapted to the subalpine-alpine belt are underlined red. Black rectangles and stars at branch nodes refer to posterior probabilities ≥ 0.98 and bootstrap values > 70.0, respectively. Grey bars specify the 95% HPD of the respective node age, red coloured bars highlight the 95% HPD for crown-group ages of subalpine-alpine clades, and black arrows at the time axis point to the node ages of the crown groups of these clades.

The crown age of central Himalayan *Meganebrius* is estimated at *ca*. 21.5 (19.15–24.09) Mya, and its separation from other lineages of the Tomocaraboides group occurred at ca. 26.7 (23.74–29.73) Mya (Fig. 2). Diversification of central Himalayan *Meganebrius* has taken place continuously during the whole Late Cenozoic. All main lineages within this group were present at least in the Mid Miocene, and all species are not younger than the Pliocene. The crown ages of *Meganebrius* lineages, strictly adapted to habitats at and above the temperature-driven forest line, are estimated to ca. 5.4 (4.19–6.70; the polytypic *Carabus epsteini*) and 6.8 Mya (5.16–8.60; the polytypic *C. tuberculipennis*) (Fig. 3).

The crown ages of the two species groups endemic to the western Himalaya, namely the *C. scheibei* group and subgenus *Imaibius*, were found to be slightly younger than those of central Himalayan *Meganebrius*: 15.8 (11.23–20.44) Mya and 15.7 (12.43–18.85) Mya, respectively.

The East Tibetan *Pagocarabus* clade evolved during the early Miocene, splitting from the East Asian *Acoptolabrus*-*Damaster* clade *ca*. 20.2 (17.39–23.05) Mya, while the crown age of the *Pagocarabus* clade is estimated *ca*. 15 (12.28–17.73) Mya. The stem and crown group ages of the East Tibetan *Rhigocarabus* clade are older compared to those of the *Pagocarabus* clade (26.2, 22.99–29.48 Mya; 19.7, 16.42–23.30 Mya).

The endemic Tibetan *Neoplesius* evolved considerably later than the Himalayan subgenera of *Carabus*. This group splits from the East Tibetan *Pagocarabus* clade *ca*. 11 (9.38–12.94) Mya, while the crown age of the central and south Tibetan *Neoplesius* species is estimated *ca*. 6.2 (5.12–7.49) Mya. The ages of *Neoplesius* taxa endemic to local valleys in the interior of South Tibet (incl. *Carabus borodini, C. danae*, *C. paulusi,* some subspecies of *C. wagae*) range between 4.7 and 0.4 Mya and are distinctly older than the local Last Glacial Maximum (LGM). The species *Carabus* (*Neoplesius*) *wagae* is the only *Carabus* taxon with a trans-Tibetan distribution, with *C.* (*N.*) *wagae wagae* from the Tibetan Himalaya and *C.* (*N.*) *wagae tanguticus* from Qinghai. These two taxa diverged during the Late Quaternary (*ca*. 0.35 Mya).

### Ancestral habitats

Since ancestral state reconstruction is sensitive to incomplete taxon sampling from outgroups^38^, *Cychrini* and *Calosoma* were removed before analysis. Estimation of habitat preferences for ancestral *Carabus* lineages revealed moderate temperate climatic conditions (Supporting Information Fig. S2). The ancestral habitat reconstruction (AHR) does not show evidence for an adaptation of any of the *Carabus* lineages to the alpine environment before the Late Miocene (Supporting Information Fig. S2). This result agrees with the phylogeny indicating that terminal monophyla characterized by the trait ‘adapted to the alpine climate’ evolved in the Himalaya and on the Tibetan Plateau at about 7 Mya (Figs. 2, 3).

## Discussion

### Emergence of *Carabus* in the HTO and high-altitude environments in the very early Miocene

Our results show that *Carabus* might have existed already during the early Eocene, with a potentially wide distribution in the pre-Palearctic Boreal during the late Eocene. The genus did probably not occur in the HTO before the Oligocene–Miocene boundary. Our dated tree indicates the arrival of *Carabus* in the HTO between *ca*. 26.7 Mya (23.74–29.73 Ma; first separation of HTO *Carabus* from non-HTO clades) and *ca*. 21.5 Mya (19.15–24.09 Ma; first cladogenesis within HTO crown groups). A similar phylogenetic age is suggested for the ground beetle genus *Pterostichus* in the southern HTO^36^, which also shows an extratropical distribution. We assume that flying ancestors colonized the HTO soon after the emergence of extensive temperate forests, which are the preferred habitat of ancient *Carabus* and *Pterostichus* ground beetles^36,39^. Recent geological studies show a separate uplift of the different parts of the HTO during early stages of its evolution^2,40^. The HTO was probably isolated from the pre-Palearctic region by an extent tropical lowland area reaching from the Paratethys eastwards to the Qaidam Basin until the Mid Miocene^41^. We suspect that at the time of its colonization by *Carabus* ancestors, the HTO was a mountain island landscape, suitable for species adapted to temperate and colder climates. Dispersal by flight was a basic requirement to overcome the extent tropical lowland areas isolating these suitable mountain habitats from the areas of origin in the pre-Palearctic Boreal.

Since the Oligocene–Miocene boundary was warmer than the middle Oligocene^3^, and because fossil floras implies a wet climate across the HTO^42–44^, we suspect that the existence of temperate forests is linked to a significant uplift of certain parts of the HTO into the temperate climatic belt during that time. Our results indicate a tropic or subtropic environment of the HTO until the late Paleogene which agrees with fossil data (Supporting Information Table S1). Despite caution is warranted within our interpretation due to potential alternative scenarios (e.g., pure vicariance, see below), our findings are in support to the assumption that the evolution of the mega-diverse high-altitude biota of the HTO has taken place almost entirely in the Neogene, when suitable habitats emerged. This would largely contradict current paleoenvironmental models derived from stable isotope paleoaltimetry. These models propose an extensively uplifted Tibetan Plateau to alpine heights, resulting in the presence of large-scale alpine paleoenvironments, since the late Eocene or even earlier^9–12,45^.

**Figure 4:**
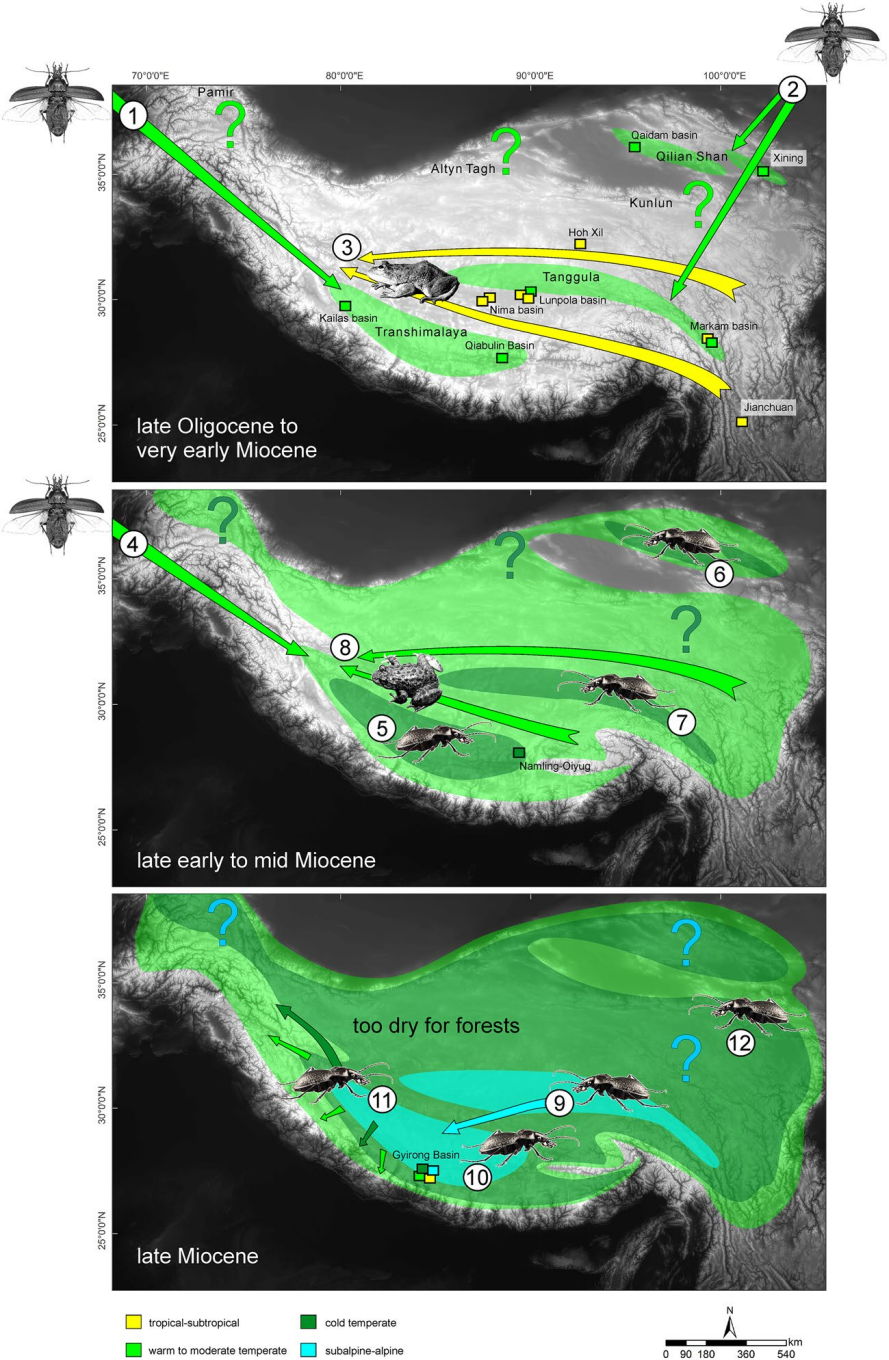
Simplified illustration of the Oligocene–Miocene paleoenvironmental evolution of the HTO modelled on the current topography. Time slices are based on the evolutionary history of *Carabus* ground beetles, amphibians^46,47,48^, and paleontological records (coloured squares, for details, see Supporting Information Table S2). Proposed extensions of temperate and alpine environments are shown as coloured areas; coloured arrows indicate dispersal events in *Carabus* and frogs. Large question marks point to regional uncertainties in the paleoenvironmental reconstruction due to the lack of paleontological and phylogeographic data. (1) Appearance of central Himalayan *Meganebrius* based on dispersal event of winged ancestor from western pre-Palearctic. (2) Appearance of east Tibetan *Rhigocarabus* and *Pagocarabus* groups based on dispersal events of winged ancestors from eastern pre-Palearctic. (3) Trans-Tibet dispersal of subtropical *Chrysopaa* spiny frogs; alternative dispersal routes are shown north and south of Tanggula Shan. (4) Appearance of western Himalayan *Carabus scheibei* group and *Imaibius* based on dispersal events of winged ancestors from western pre-Palearctic. (5–7) Radiation of wingless central Himalayan *Meganebrius* [5] and east-Tibetan *Rhigocarabus* and *Pagocarabus* [6, 7] in the course of ongoing surface uplift of the respective parts of the HTO. (8) Trans-Tibet dispersal of warm temperate *Allopaa* spiny frogs; alternative dispersal routes are shown north and south of Tanggula Shan. (9) Dispersal of wingless subalpine *Neoplesius* from east to south Tibet and subsequent diversification. (10) Evolution of subalpine-alpine lineages within central Himalayan *Meganebrius*. (11) Range shift towards the HTO margins in south Tibetan *Carabus* and amphibians (*Nanorana*, *Scutiger*) adapted to temperate climates in response to the surface uplift, cooling, and drying of Tibet. (12) Ongoing radiation of east Tibetan *Carabus* and amphibians in today's western China.

Our divergence time estimates also show that during the very early Miocene, different species groups of *Carabus* started to diverge almost simultaneously in two different parts of the orogenic system: in the southern central HTO (central Himalayan *Meganebrius*) and elsewhere in its eastern or northeastern region (*Pagocarabus* and *Rhigocarabus* clades; Fig. 4). An early colonization of the southern HTO margin by *Carabus* seems possible and is supported by geoscientific evidence for high elevations in the Lhasa Terrane which prevailed at the beginning of the Neogene at latest (overview in^40^). Because today’s distribution of the species-diverse central Himalayan *Meganebrius* is restricted to the Nepal Himalaya, we suspect a spatially narrow area of suitable habitats in the southern central Paleo-Tibet during the time at which ancestral lineages may have occurred (Fig. 4). Within this area, local climatic conditions might have been cold and humid enough due to highly elevated terrain, providing suitable environmental conditions for temperate forests during that period. Our hypothesized southern mountain range, which we assume was geographically separated from other mountains to the north by wide lowland areas, corresponds in parts to the Transhimalaya (Gangdese Shan) as modeled by Spicer and colleagues^2^.

Alternatively, vicariance mechanisms may have isolated central Himalayan *Meganebrius* from its pre-Palearctic sister taxon by extirpation of intervening relatives. We cannot exclude this scenario; however, we are not aware of any factor that could explain such a large-scale extinction event in this complex mountain system. We consider this scenario as unlikely because other *Carabus* lineages, with habitat preferences similar to *Meganebrius* and partly syntopic with these species, colonized also areas of the HTO since the late early Miocene where *Meganebrius* is absent (see below, *Carabus scheibei* group and subgenus *Imaibius*).

Today, the lower limits of the vertical distribution of *Carabus* taxa on the southern slope of the central Himalaya range between 1800 and 2000 m and are associated to the lower limit of the lower cloud forest zone^49^. Due to the significantly warmer climate during the early Miocene^3^, the vertical (temperature-bound) range limits of HTO *Carabus* were supposedly higher than today. The early to mid-Miocene climate was characterized by global mean annual temperatures (MAT) about 5–6 K higher than today^3^. However, those values may not apply to low latitudes and earlier evolutionary stages of the HTO as shown by the CLAMP (Climate Leaf Analysis Multivariate Program) data of fossil floras^50^. These data show MAT across the central and eastern Himalaya that were only 1–2 K higher during the mid-Miocene compared to present temperatures. Local lapse rates along mountain slopes are markedly impacted by mass elevation and lee effects^51^. These effects may have changed fundamentally in the course of the spatio-temporal development of the topography of the HTO. Similar to what is observed for the HTO tree lines, an increase in seasonal temperature due to mass elevation and lee effects shifts the recent vertical distributions of ground beetles significantly from the Himalayan south face to the north towards the Transhimalaya against the latitudinal trend^52,53^. However, during the early Miocene, mass elevation and lee effects on the southern slope of HTO were probably less impactful because the Greater Himalaya was still of lower elevation. This scenario agrees with recent CLAMP analyses for the warm temperate Qiabulin forest flora of the very early Miocene, north of Mt. Everest, indicating a paleoelevation of 2300 ± 900 m for this flora^54^. Given the spatio-temporal and ecological overlap of the Qiabulin flora with today's *Meganebrius* fauna, this flora may have provided suitable conditions for these beetles in the early Miocene. Indeed, the Qiabulin flora has thrived only about 300–400 m above today's lower vertical distributional border of *Meganebrius* on the southern slope of the Greater Himalaya.

Assuming a lower vertical distributional limit between 2200 and 2400 m of the central Himalayan *Meganebrius* during its earliest evolutionary history and considering an impact of mass elevation and lee effects towards the interior of the HTO (although less pronounced than today), the maximum elevation of mountain ranges in central South Tibet must have been lower than 3000 m during the very early Miocene. Moreover, since the recent distribution of *Meganebrius* is restricted to the Central Himalaya, we assume that ancestral species occurred likewise in a narrow area on the southern central margin of the HTO. If so, there might have been significant sloping not only to the south, but also to the north, west, and east of the mountain ranges in central South Tibet, with vast areas characterized by tropical climate unsuitable for *Carabus* beetles (Fig. 4). This could imply that today's eastern and western parts of the Transhimalaya and Greater Himalaya, and the central parts of Tibet, were significantly lower than the southern central HTO during the very early Miocene.

### Topography in central South Tibet during the late-early to mid-Miocene

**Figure 5:**
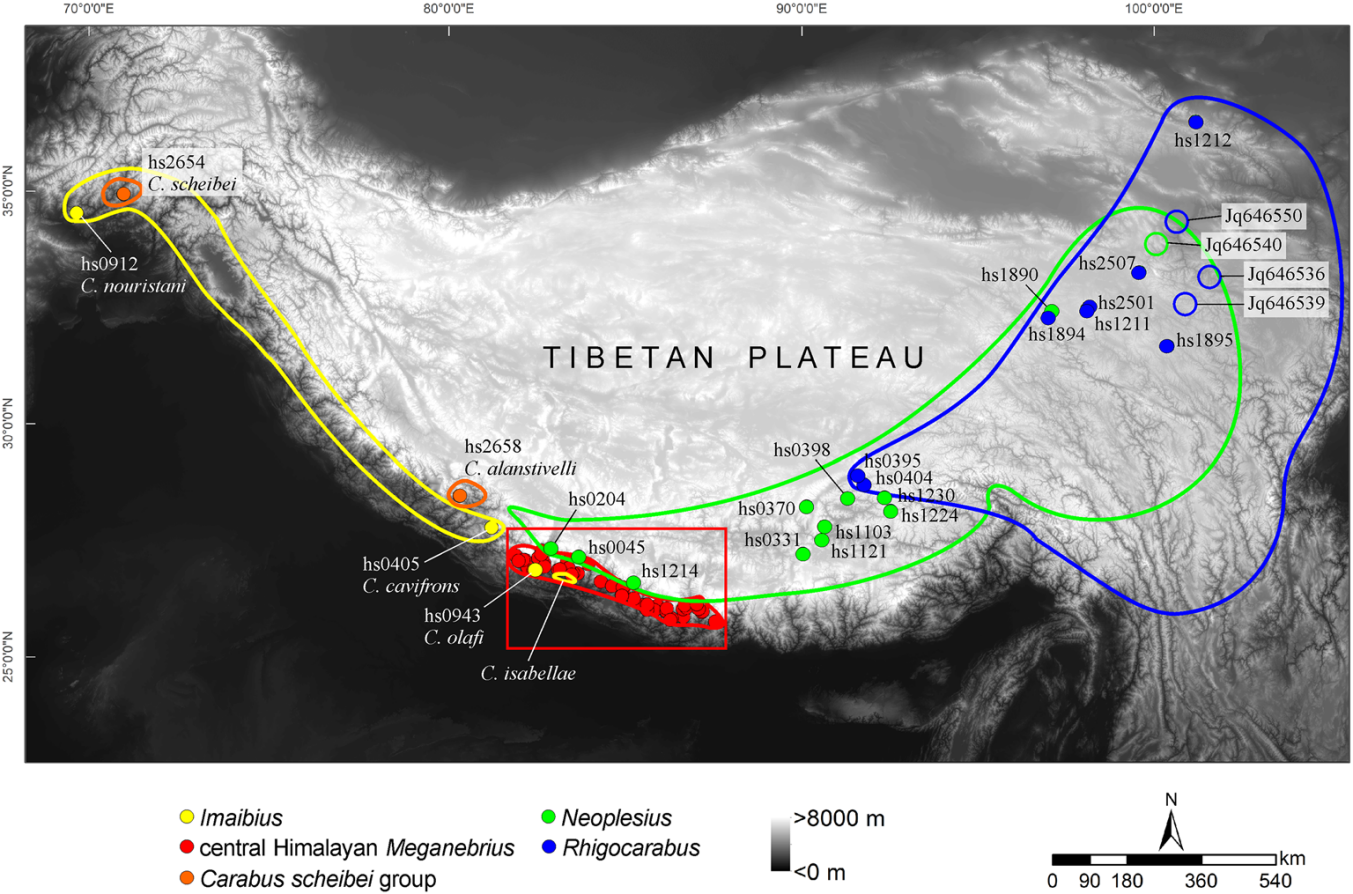
Overview of distributional areas (continuous lines) and sampling localities (coloured dots with voucher ID) of *Carabus* species groups endemic to the Himalaya and the central Tibetan Plateau. Unfilled circles indicate GenBank vouchers of which the exact locality is unknown. Different colours mark different species groups. Note the isolated position of central Himalayan *Meganebrius* (red) within the Greater Himalaya of Nepal, the wide distributional gap of *Carabus* in the Greater Himalaya east of Nepal, and the disjunct distribution patterns in the subgenus *Imaibius* (yellow) and the *Carabus scheibei* group (orange). For sampling locations of *Meganebrius* (red framed box) see Fig. 6. Samples from the Indian Himalaya (distributional area of *Imaibius*) and the easternmost parts of Tibet (distributional area of *Neoplesius* and *Rhigocarabus*) were not available.

**Figure 6:**
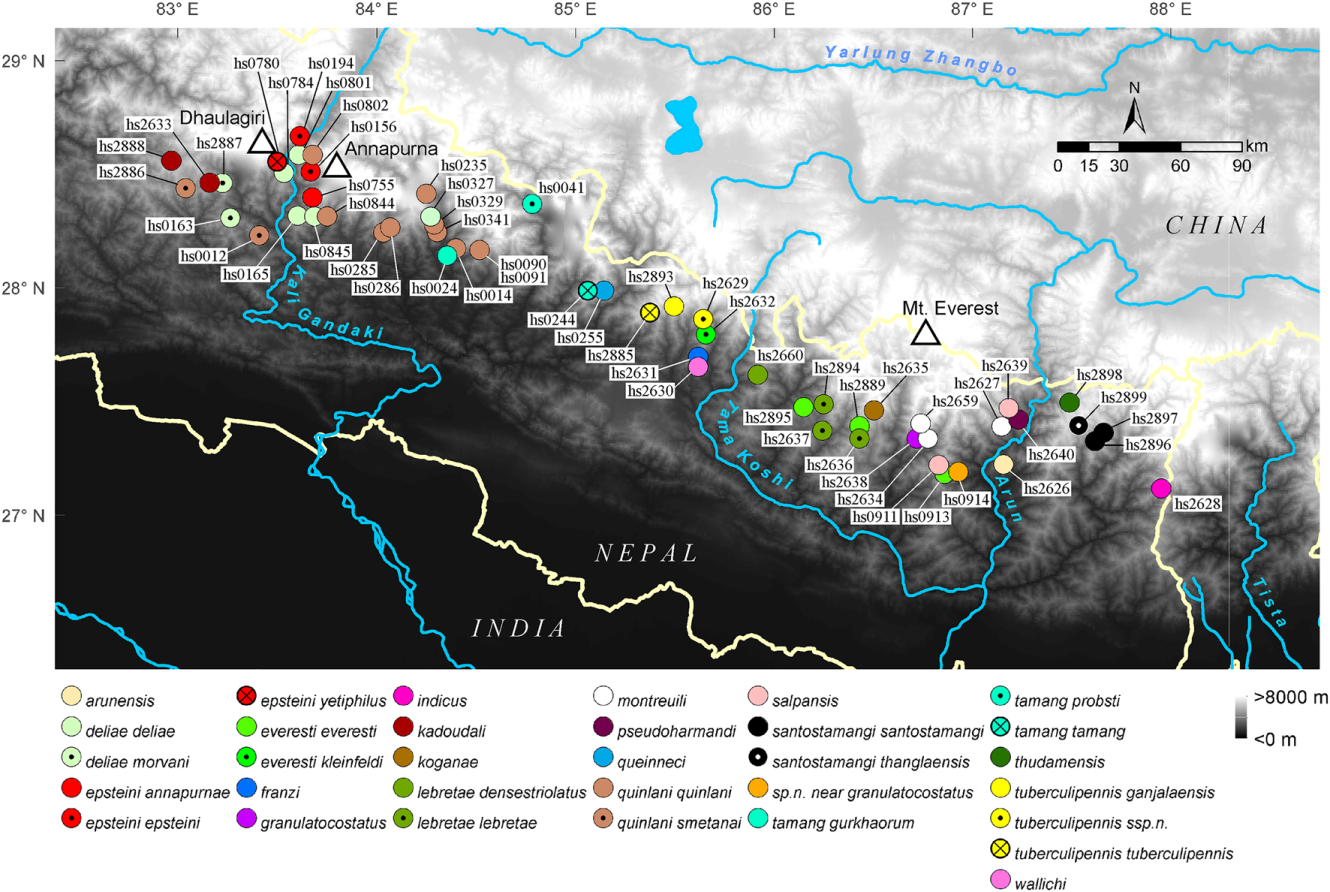
Sampling localities of central Himalayan *Meganebrius* species (coloured dots with voucher ID). Different colours mark different species; dots and crosses indicate different subspecies. The map section corresponds to the red framed box in Fig. 5 and spans the whole distributional area of the *Meganebrius* group.

The evolution of the *Carabus* lineages endemic to the Western Himalaya, namely *Imaibius* and the *C. scheibei* group, provides some information on the potential development of paleoenvironments along the southern and western HTO margins during the late-early Miocene. These taxa began to diversify *ca*. 6 My later than the Central Himalayan *Meganebrius* (Fig. 2). Today, these species groups have their eastern distributional border in the western part of the central Himalaya. The eastern distributional edge of *Imaibius* overlaps with the western distributional edge of central Himalayan *Meganebrius* in the massifs of Annapurna and Dhaulagiri (Figs. 5 and 6) where the respective local endemic species occur syntopic in the warm-temperate elevational belt. This pattern indicates that at the time of arrival of *Imaibius* and the *C. scheibei* group in the HTO in the late-early Miocene, temperate forests may have extended more to the western HTO margin while the central part of the southern HTO was probably already too high to be colonized by these beetles (Fig. 4). We assume that warm temperate forests were widely fragmented by higher uplifted mountains dominated by cold temperate environments. This scenario is supported by fossil evidence of the cold temperate Namling flora in the central Transhimalaya^20,55^ (Fig. 4).

The disjunct distributional pattern of the western Himalayan *C. scheibei* group appears particularly informative for the reconstruction of the ancestral distributions of *Carabus* in the southern parts of the HTO. This group contains the polytypic species *C. scheibei* occurring north of the Indus transverse valley, and *C. alanstivelli* in the Karnali River system of Far West Nepal (Fig. 5). A very similar distribution can be found in the ground beetle *Ethira* clade and in spiny frogs of the genus *Nanorana*, supporting the 'out of Tibet into the Himalayan exile' hypothesis^36,46^. Based on this hypothesis, species of the *C. scheibei* group could be descendants of a *Carabus* lineage originally distributed along the western face of Paleo-Tibet. In the course of the uplifting and drying out of Tibet they were forced to follow the horizontal habitat shifts within the epigenetic transverse valleys from Tibet’s interior to the HTO margin formed by the Greater and Lesser Himalayas. Such scenario might also apply to development of the disjunct distribution of *Imaibius* with two local endemic species on both sides of the Kali Gandaki transverse valley (Fig. 5). We therefore assume that the estimated crown ages of 15.8 and 15.7 Mya in the *C. scheibei* group and *Imaibius* could be linked to the existence of temperate forest habitats of these species in the western Paleo-South Tibet.

Unfortunately, a recent phylogeographic analysis of Himalayan herpetofauna^56^ did not consider the Tibetan-origin hypotheses for these organisms but linked changes of in situ diversification and dispersal rates over time directly to the age of the Himalaya to test different geological uplift models. By focusing primarily on the temporal dimension, that study supposes a beginning of the uplift of the Himalaya in the Paleocene with a rapid rising of it during the whole Miocene. Our results do not support this scenario but indicate a direct relation of Himalayan taxa evolution to the significant surface uplift in the central South Tibet at about the Oligocene–Miocene boundary, followed by the rising of adjacent orogenic parts, including the Greater and Lesser Himalayas. The fauna of paleo-South Tibet might have been step-wise 'squeezed out' of Tibet towards the HTO margins, tracking suitable habitats along the Himalayan transverse valleys^36^.

Our belief of the presence of cold temperate environments in large parts of central South Tibet during the late-early Miocene is supported by fossil records. For example, the presence of the moderately cold-temperate Namling forest flora (~ 15 Mya^55^) in the central part of the Transhimalaya matches almost exactly the estimated time when western Himalayan *Carabus* started to diversify. However, it must be noted, that there is higher variation in the vertical distribution estimated for this flora, ranging between 2500 and 3000 m^20^, and up to > 5000 m^50^. Today’s closest relatives of this flora occur along the southern slopes of the central Himalaya at the middle cloud forest zone^49^ (ca. 2500–3000 m) syntopic with central Himalayan *Meganebrius*. If, however, at the same time at which this flora thrived, the adjacent Everest area was uplifted to significant height as previously proposed^29^, mass elevation and lee effects would have significantly influenced the local climate and may have forced Transhimalayan plants and animals to shift their vertical ranges upward. Furthermore, the warmer climate during the mid-Miocene^3^ argues for higher vertical distributions of the cold-adapted biota compared to the present. Hence, we suspect a higher distribution of the Namling flora than Zhou et al.^20^ estimated. On the other hand, the CLAMP-based calculation for this flora^40^ (high alpine environment) might be overestimated (see re-evaluation of this method by^57^). Irrespective of these uncertainties, a significant sloping of paleo-South Tibet from its center to the West at *ca*. 15 Mya seems likely because of the existence of a moderately cold-temperate Namling flora simultaneously with the appearance of an endemic *Carabus* lineage (*C. scheibei* group) along the western margin of paleo-Tibet. It is therefore plausible to suppose that the central part of southern HTO provided habitats for cold temperate biota already during the late-early Miocene, while warm temperate forest may have dominated along mountain crests on the western HTO margin (Fig. 4).

Sloping of the southern central HTO towards its northern and eastern faces could have been more pronounced than to its western face, at least until the mid-Miocene. From our phylogenetic tree, there is no indication for dispersal of *Carabus* faunas from the southern to the eastern HTO margin and vice versa until the late Miocene. One reason could be a dispersal barrier such as a vast and lower elevation area in between (Fig. 4). This area may correspond to the central Tibetan Valley identified by Spicer et al., but, according to these authors, existed only until the end of the Paleogene^2^. At the time when central Himalayan *Meganebrius* began to diversify, different *Carabus* lineages with an origin in the eastern pre-Palearctic evolved probably along the eastern or northeastern margin of the HTO. Estimated crown ages for the two East Tibetan species groups, *Rhigocarabus* and *Pagocarabus*, date to the early Miocene (Fig. 2). Like the *Carabus* fauna of paleo-South Tibet, the highly diverse East Tibetan *Carabus* fauna may have evolved geographically separated from other parts of the orogenetic system. In fact, *Carabus* faunas from different plateau margins apparently did not come into contact before the latest Miocene.

Noteworthy, a recent phylogeographic study in spiny frogs provides support for the scenario of a pronounced topography of Tibet in the early Miocene, suspecting trans-Tibet dispersal events of the subtropical *Chrysopaa* during the late Oligocene and for the warm-temperate *Allopaa* during the early Miocene^47^. These findings imply climatic conditions suitable for the amphibians in vast areas of paleo-Tibet. Considering this information, together with our *Carabus* data herein, and the paleontological evidence for the contemporary presence of cold temperate environments on the central HTO and its eastern margin^20^; Table S1), a more pronounced paleosurface relief compared to today can be assumed (Fig. 4). The high dynamic of the paleotopography of Tibet's interior may has prevented dispersal events of the wingless beetles at least until the Late Miocene, leaving its traces in the phylogeographic structure of the cold-adapted species groups. High-altitude amphibians such as lazy toads and spiny frogs evolved apparently in separate parts of the HTO; the subsequent trans-Tibet dispersal of subalpine-alpine species were estimated to have occurred from the latest Miocene at the earliest^47,48^.

### Indications for Late Miocene development of alpine environments in the HTO

Contemporary alpine environments may have existed in the southern, central, and eastern parts of the HTO at the end of the Miocene (Fig. 4): Given the crown ages of central Himalayan *Meganebrius* species groups, which are strictly adapted to habitats at the forest line and above, and assuming a colonization of such habitats by these beetles as soon as they were available, alpine environments would have developed in the Himalaya around 7–5 Mya (Fig. 3, Supporting Information Fig. S2). Also, our phylogeny of the subalpine-alpine *Neoplesius* hints to the presence of extensive forest line habitats in Tibet. This group originated apparently at the eastern HTO margin and started to diversify in South Tibet *ca*. 6.2 Mya (Fig. 2; Supporting Information Fig. S2). However, all but one of the south Tibetan *Neoplesius* lineages remained restricted to certain massifs and high valleys of the Tibetan Himalaya and Transhimalaya (Figs. 2, 4). A trans-Tibet dispersal event of *Carabus* beetles seemingly did not occur before the late Quaternary, indicated by the estimated node age (0.35 Mya) of *C.* (*Neoplesius*) *wagae wagae* from the Tibetan Himalaya and *C.* (*N.*) *wagae tanguticus* from Qinghai (Fig. 2). The high local endemism observed in *Neoplesius* and many other alpine groups of ground beetles of southern and central Tibet^52^ is in contrast to the hypothesis of a continuous alpine landscape across today's Tibet before the Quaternary. From the latter “early Neogene alpine Plateau” scenario^58,59^, trans-Tibet distribution patterns would be expected also for those lineages of wingless Tibetan ground beetles which have a phylogenetic age significantly older than the Quaternary. Such cases, however, haven’t been described so far. We, therefore, tend to assume that alpine environments may have undergone periods of long-lasting separation since their first appearance in the late Miocene.

Our conclusions are further underlined by potential interactions of the late Neogene topographic and climatic developments in the southern, central, and eastern parts of the HTO. The significant surface uplift of Southeast Tibet and the eastern Himalaya during the late Neogene^60,61^ must have strengthened mass elevation and lee effects. Particularly an increase of effective blocking of the humid air masses of the Indian and East Asian monsoon can be assumed^62,63^, and, thus, a warming of the Tibetan interior enabling its biota to upslope their distribution ranges. If so, and if large areas of Tibet were part of the subalpine-alpine belt during the late Miocene, as our *Carabus* data suggest, this belt might have become successively fragmented due to the increasing mass elevation and lee effects. The large coherent alpine area of today's Tibetan Plateau could result from the surface uplift that continuously shifts the plateau into the alpine belt against the trend of climate warming of Tibet's interior due to mass elevation and lee effects^52^. Orogenic rising along the HTO margin, climate response, and ongoing uplift in the HTO interior could have caused trans-Tibet dispersal events of subalpine-alpine *Carabus* at different times since the end of the Miocene.

Basically, the age of alpine environments across the HTO is of particular interest with respect to the inconsistencies between phylogenetically predominantly young alpine taxa and geoscientific models, which suppose a highly uplifted Tibetan Plateau during the Eocene (reviewed in^35^). A *tabula rasa* due to an extensive Pleistocene ice sheet that supposedly covered large parts of the HTO (^64^ and elsewhere) is still one of the commonly believed scenarios for the Tibetan Plateau. Such large-scale extinction events would be reflected in the node ages of alpine HTO lineages^35^. However, Kuhle's ice-sheet hypothesis has long been rejected^65^, and the extent of glaciers on the HTO is well-known at least for the LGM, showing only moderate maximum glacier advances (overview in^66^). Moreover, paleoglaciations of the Tibetan Plateau were generally low, with an average shift of the equilibrium line altitude (∆ELA) of 494 ± 280 m for pre-LGM glacial deposits^67^. A moderate LGM temperature depression on the Tibetan Plateau of about 3–4 K could have enabled the survival of a highly diverse subalpine-alpine fauna and flora^68^. Based on our phylogeny, local endemics of the *Carabus* subgenus *Neoplesius* might have persisted in central parts of the Tibetan Himalaya and Transhimalaya since their arrival in the Late Miocene. Apparently, Quaternary climatic oscillations can neither explain the young node ages in phylogenies of alpine HTO taxa nor resolve the discrepancies between phylogeographic and geoscientific results as highlighted previously^35^. In light of our dated phylogeny, we assume that extensive alpine environments across central Tibet (connecting opposite plateau margins and the Himalayas with eastern Tibet) developed at the end of the Miocene. Small separated alpine environments may have existed on top areas of some prominent mountain ranges of the HTO already before that time but were probably not colonized by the *Carabus* beetles.

The fact that no fossil evidence exists for an alpine biota in the HTO before the late Miocene indirectly supports our model of a young age of the alpine environment (Fig. 4, Supporting Information Table S1). It must be noted that few phylogeographic studies in plants argue for the existence of an alpine vegetation belt across the HTO already during the Paleogene^58^. Accordingly, an Eocene origin of alpine *Gentiana* and, thus, very high elevations in the HTO are proposed based on stable isotope analyses and the assumption of an ancestral adaptation of this genus to alpine environments. An alpine origin of *Gentiana* was recently refuted^33^ but at the same time a pre-Neogene emergence of other alpine flora on the HTO was brought to attention. Yet, a reassessment of these data revealed a potential bias in methodological quality and demonstrated that a much younger age of alpine habitats (*ca*. 7.5 Mya) is supported^69^, which is consistent with the estimated age in our *Carabus* phylogeny.

In summary, soil arthropods, like ground beetles, have widely colonized the high-altitude environment as soon as they arrived in the HTO. Ground beetle phylogenies could therefore echo the spatio-temporal evolution of this environment. Our *Carabus* phylogeny provide indications in support of a young age of both temperate (late Oligocene to very early Miocene) and alpine (late Miocene to Quaternary) environment in the HTO. Our results disagree with other paleoaltimetric models for the HTO by the following aspects:

i. The emergence of *Carabus* in the HTO during the Oligocene- Miocene boundary suggests subtropical or warm-temperate environments, and, thus, probably low average elevations in the orogenetic system before that period. In contrast, results from stable isotope analysis imply paleoelevations close to present heights since the late Eocene or even earlier^13–16^. If so, large cold-temperate and alpine habitats should have existed across the HTO long before *Carabus* colonized these areas. Given the high phylogenetic age of the genus and the strong dispersal ability of its winged ancestral lineages, a "non-colonization" of such vast, suitable habitats does not seem plausible, although it cannot be excluded.
ii. A higher uplifted area on the southern central margin of the HTO, near today's Nepal, may have been present during the Miocene. This concept conflicts with geoscientific models of the HTO paleotopograpy, which postulate a highly elevated southern Tibetan Plateau during the late Paleogene that covered the entire West–East extension of the orogenic system^24,33^. If those models are true, we would expect an initiation of the *Carabus* evolution *along* the edges of the HTO, e.g., in the Northwest because of short distances to temperate habitats in the pre-Palearctic region. Moreover, recent central Himalayan *Meganebrius* should occur along the entire Himalayan arc as well as in southeastern Tibet. However, *Carabus* evolved initially in the center of the southern HTO, as indicated by our phylogeny, and the distribution of central Himalayan *Meganebrius* remained restricted to this single area. All western Himalayan *Carabus* lineages evolved apparently some million years later.
iii. Our *Carabus* phylogeny does not provide evidence for the geoscientific model of a stepwise rise and growth of the Tibet-Qinghai Plateau to the north and east^10,24^. If so, the high-altitude fauna of the southern HTO should be phylogenetically older than that of the eastern HTO margin. However, the almost contemporary presence of *Carabus* on the southern and eastern margin of the HTO indicates temperate habitats and thus moderately elevated areas in very distant parts of the orogenetic system during the very early Miocene.
iv. Finally, our results do not support the presence of a highly elevated altiplano during the Miocene^12^. An evolution of *Carabus* in disjunct centers in the HTO, probably preventing a faunal exchange within temperate *Carabus*, seems plausible. Only species adapted to the subalpine-alpine zone might have been able to disperse across central Tibet during the Late Cenozoic. Consequently, the paleotopography of Tibet could have been much more pronounced than today and its plateau-like shape might be a young geomorphological feature of the Pliocene–Quaternary.

### Limits of the study and future research

Our approach generally suffers from the critical assumption that niches are stable over time. In fact, in vertebrate ectotherms, niches seem to be significantly higher conserved through time than in endotherms^70^. The more this applies to soil arthropods, like ground beetles, which are species with narrow trophic niches, and limited niche plasticity^71–73^.

There are also issues specific to the ancestral habitat inference, especially due to the fact that no current methods can merge areas (habitats) backwards in deep times^74^, making it difficult to us to assign species within a well-defined temporal paleogeographic framework without imposing a specific historical-biogeographic scenario a priori. Another limitation in our study resides in the availability of data from the high mountains along the northern margin of the HTO, particularly the Karakoram and Altyn Tagh mountain ranges where the genus *Carabus* is obviously absent. Information from these parts of the orogenic system might be crucial for revealing the evolution of the HTO fauna (see Fig. 4). We therefore encourage the investigation of additional species groups of ground beetles and other low dispersing soil arthropods in these mountains. Further challenges may arise from existing uncertainties in molecular dating. Although a recent phylogenetic study in the *Carabus* sister group *Calosoma* largely supports our dating approach^75^, in previous studies younger evolutionary ages were estimated for both these groups^37,76^. Latter approaches would indicate slightly younger ages for the paleoecological scenarios presented here (contrasting even more with results from isotopic studies).

Our results may help better understand the highly complex geomorphological and paleoecological history in the southern parts of the HTO. In any case, they highlight the importance of considering alternative scenarios for the evolution of the HTO, which so far has been mainly approached by geological models. The *Carabus* data offer indications that today's HTO's topography and environmental conditions are relatively young features. An asynchronous surface uplift might be characteristic for the different parts of the HTO and its respective geological units.

## Methods

### Taxon sampling

Representatives of 43 subgenera of *Carabus* are included in the analyses, with the *Carabus* sister taxon *Calosoma*, and the Carabinae tribe Cychrini used as outgroups (in total 193 samples). The sampling considers all subgeneric *Carabus* species groups occurring in the Himalayas and the central HTO, these are (i) *Imaibius* (west Himalayan endemic), (ii) *Meganebrius* (Himalayan endemic), (iii) *Neoplesius* (Tibetan Plateau endemic), and (iv) *Rhigocarabus* (East Tibet endemic); for distributional areas of these subgenera see Figs. 5 and 6. Our sampling contains three species of *Imaibius*, 10 species of *Rhigocarabus* and related *Hypsocarabus* and *Sinoleptocarabus*, as well as 18 specific and subspecific taxa of *Neoplesius* and related *Calocarabus*, *Cupreocarabus*, *Pagocarabus* and *Pseudocranion*. The *Neoplesius* sample comprises most of the taxa occurring in the Transhimalaya and Tibetan Himalaya. For *Meganebrius* all described species and most of the subspecies are included in the analyses together with two hitherto undescribed taxa (of these, *Carabus dilatotarsalis*, is considered a junior synonym of *C. lebretae lebretae*; unpubl. data). For details of the taxon sampling see Supporting Information Tables S2, S3, S7 and S8.

### Sequence data acquisition

Genomic DNA was extracted from femoral or thoracic muscles of specimens preserved in ethanol or of dried museum specimens, using the DNeasy Blood & Tissue Kit (Qiagen, Venlo, Netherlands) following the manufacturer's protocol. In total, 9437 bp of two mitochondrial (COI, 1444 bp; ND5, 1028 bp), three ribosomal nuclear (18 s, 1886 bp; 28 s, 1048 bp; ITS2, 1456 bp), three protein-coding nuclear markers (CAD: carbamoylphosphate synthetase domain of the rudimentary gene, 811 bp; PepCK: phosphoenolpyruvate carboxykinase gene, 623 bp; wg: wingless gene, 439 bp), and the non-protein-coding nuclear HUWE1 locus (702 bp) were amplified via the polymerase chain reaction (PCR) using basic protocols recommended by the manufacturers (Supporting Information Table S3, Fig. S3); primers and PCR conditions are presented in Supporting Information Tables S4 and S5. PCR products were purified using the mi-PCR Purification Kit (Metabion, Planegg, Germany), and Sanger sequenced on an ABI 3730 XL sequencer by LGC Genomics (Berlin, Germany).

### Sequence alignment

Ribosomal RNA (rRNA) 18s, 28s, and ITS2 sequences were aligned based on their secondary structures using RNAsalsa 0.8.1^77^. As the initial input, we used constraint files based on the secondary structures of *Bembidion chalceum* 18S rRNA downloaded from http://www.rna.ccbb.utexas.edu (EF648647) and *Apis mellifera* 28S rRNA which is provided with the RNAsalsa package. Before aligning ITS2 data, sequences were annotated and trimmed using the ITS2 database^78^, and references therein). We predicted the secondary structure of ITS2 for an arbitrarily chosen sequence (*Platyceps creutzeri*) using the Vienna RNAfold web server^78^ and default settings. The sequences were then aligned with our data using the MUSCLE algorithm in MEGA X^79^. Starting with these initial alignments and the respective constraint file, RNAsalsa implements a workflow for both RNA secondary structure prediction and enhanced structural alignment that results in a final multiple sequence alignment together with a consensus structure.

The sequences of the protein-coding genes (mtDNA: COI, ND5; nuDNA: wingless, pepck, cad) and the non-protein-coding nuclear locus (HUWE1) were also aligned with MUSCLE using default settings in MEGA X. Alignment based on nucleotides and amino acids produced similar results, since no ambiguities, such as deletions, insertions, or stop codons, were found.

### Phylogenetic reconstruction

The final concatenated rRNA, mtDNA, and nuDNA sequence dataset consisted of 155 species and subspecies and contained 9437 alignment positions, of which 2718 were phylogenetically informative. Nuclear data were unphased as most taxa had only single representative individuals. The dataset was partitioned a priori by genes and codons, and PartitionFinder 1.1.1^80^ was applied to optimize partitions using linked branch lengths, the Bayesian information criterion (BIC), the *greedy* search algorithm, and the substitution models implemented in MrBayes (Supporting Information Table S6). We inferred a Bayesian inference (BI) tree based on our final dataset using MrBayes v. 3.2.6^81^. For the rRNA stem regions, the doublet model (16 × 16) proposed by Schoniger and von Haeseler^82^ was assigned in the Bayesian analysis. For this procedure, unambiguous stem pairs were derived based on the consensus structure from RNAsalsa and specified in the MrBayes input file. For the analysis of the remaining positions, the standard 4 × 4 option was applied using a GTR evolutionary model for all nucleotide partitions. The site-specific rates were set variable.

MrBayes was run for five million generations, sampling trees every 500th generation and using a random tree as a starting point. Inspection of the standard deviation of split frequencies after the final run as well as the effective sample size value of the traces using Tracer v1.7.1^83^ indicated convergence of Markov chains. In all analyses, four parallel Markov chain Monte Carlo simulations with four chains (one cold and three heated) were run. The first 25% of the samples of each run were discarded as burn-in. Based on the sampled trees, consensus trees were produced using the sumt command in MrBayes. We also inferred a maximum likelihood tree using RAxML v.8.2.12^84^ with the GRTCAT approximation, 1000 bootstrap replicates, and with the partition scheme as selected by PartitionFinder (Supporting Information Table S6).

### Molecular dating

Based on the full concatenated dataset, divergence dates were estimated using BEAST2 v. 2.6.2^85,86^. Data were partitioned based on the scheme selected by PartitionFinder (Supporting Information Table S6), with unlinked substitution models, unlinked uncorrelated relaxed clock models, and a linked tree model. It is impossible to consider secondary structure information in BEAST (ambiguities are treated as unknown data, so we did not remove stem regions)—thus, all positions of the respective rRNA partition were treated under the same evolutionary model. Age constraints were derived from a previous calibration analysis of the phylogeny of *Carabus* ground beetles^34^, which agrees widely with the results of a latest analyses in the sister group *Calosoma*^75^ root (the most common ancestor [MRCA] of Cychrini/Carabini): 84.7 Mya, 69.7–103 (lognormal, M: 4.439, S: 0.0995); the MRCA of *Calosoma*/*Carabus*: 56.1 Mya, 47.8–67.3 (lognormal, M: 3.50, S: 0.148, offset at 23.03 justified by the minimum age of the fossil record^87^; the MRCA of *Tachypus*/*Ctenocarabus*: 34.3 Mya, 28.7–41.0 Mya (lognormal, M: 3.535, S: 0.091); the MRCA *Carabus rugosus/morbillosus*: 17.4 Mya, 14.8–20.4 Mya (lognormal, M: 2.855, S: 0.083); the MRCA of *Carabus riffensis* and European *Mesocarabus*: 22.9 Mya, 19.6–26.7 Mya (lognormal, M: 3.13, S: 0.078); the MRCA of *Eurycarabus/Nesaeocarabus*: 22.10 Ma, 18.9–25.7 Mya (lognormal, M: 3.0935, S: 0.079). We constrained *Calosoma* as well as Cychrini to be monophyletic^75^. Analyses were based upon five independent BEAST runs with a chain length of 100 Mio each, a thinning interval of 10,000, a lognormal relaxed clock model, a Yule tree prior, a random tree as starting tree, and the site models selected using bModelTest package^88^ implemented in BEAST2. Runs were then combined with BEAST2 LogCombiner v.2.6.2 by resampling trees from the posterior distributions at a lower frequency, resulting in 9,005 trees. Convergence and stationary levels were verified with Tracer by a standard deviation of split frequencies < 0.01 as well as an effective sample size value > 200 of the traces. The final tree was obtained with TreeAnnotator v.2.6.2 and visualized with FigTree v.1.4^89^.

### Paleoecological and paleoelevational estimations and ancestral state reconstruction

For the reconstruction of paleoenvironments in the HTO we consider two basic facts: (i) an origin of *Carabus* ground beetles in extratropical areas of the pre-Palearctic region^37,39^, and (ii) long-term stasis in their climatic niches (niche conservatism), i.e., the tendency to keep ancestral ecological niche characteristics over time. The latter implies that speciation takes place primarily in geographic, not ecological, dimensions^90,91^; an adaptation of extratropical *Carabus* to tropical environments during its evolutionary history can be considered unlikely^37,39^ (see Supporting Information Text for further details). In this sense, the estimated divergence times of temperate and cold-adapted *Carabus* occurring in the today’s HTO are indicative of the time of speciation happening in response to changes in the distribution of paleo-high montane forest and, thus, the presence of temperate or colder climates in the orogenetic system. Because the HTO has grown within low latitudes^92^, and today the Himalayan foothills are still situated in the tropical zone^49^, it is reasonable to assume that the occurrence of both, extratropical paleoenvionments and *Carabus* beetles, are associated with areas uplifted to significant elevations. Elevational records of recent *Carabus* species of the HTO are presented in Supporting Table S2. The vertical distributions of all these species are situated in the zonal areas of the temperate and boreal forests and alpine steppe of the respective parts of the todays HTO^49^. Like the Coexistence approach, which was developed for fossil floras^93^, we use ecological information from recent species of certain *Carabus* lineages to derive the environmental adaptations of the ancestral species of these lineages. Based on ecological characteristics of *Carabus* ground beetles^37,39^ we assume that adaptation to the alpine environment is a derived pattern. The presence of this “alpine” character state in all species of a certain clade represents a synapomorphic feature of this clade. Consequently, the crown group age of this clade can be used to date the minimum age of alpine environments in the area of origin of the crown group (synapomorphy based approach). Due to uncertainties regarding lapse rates and regional temperature regimes during deep times, paleoelevations cannot be derived directly from vertical distributions of the recent species^2,94^. Therefore, we translate our paleoenvironmental scenarios into paleoelevational estimations in those cases where paleoelevational scenarios exist from the literature for the same part of the HTO and geological period as our data.

We use ancestral habitat reconstructions as an additional tool to verify synapomorphy-based hypotheses of the onset of alpine adaptations of the *Carabus* lineages. The ancestral habitat type of the *Carabus* lineages was estimated with RASP v 4.2^95^ based on the BBM (Bayesian Binary MCMC) analyses, using the BEAST2 consensus tree as input. Outgroups (*Calosoma*, *Cychrini*) were removed before analysis, as recommended^38^. We coded five states to the tips of the tree according to temperature preferences of the extant species: A, warm temperate (= lower cloud forest zone in the HTO); B, temperate (= middle cloud forest zone in the HTO); C, cold temperate (= upper cloud forest zone in the HTO); D, subarctic (= subalpine); E, arctic (= alpine) (Supporting Information Table S7). Analysis run for 500,000 MCMC cycles with 10 chains and a sampling frequency of 1000.

### Supplementary Information

Supplementary Information.

## Data Availability

All data needed to evaluate the conclusions in the paper are present in the paper and/or the Supporting Information. All sequence data used in the analyses are deposited in GenBank and listed in the Supplementary Table S3.
