# Supplementary material for: Molecular phylogeny of mega-diverse Carabus attests late Miocene evolution of alpine environments in the Himalayan–Tibetan Orogen
Source: Sci Rep. 2023 Aug 15;13:13272. doi: 10.1038/s41598-023-38999-6 (PMC10427656; doi:10.1038/s41598-023-38999-6)
Supplement: Supplementary file 1 — Supplementary Information. [file 41598_2023_38999_MOESM1_ESM.docx]

**Supplementary Information for**

Molecular phylogeny of mega-diverse *Carabus* attests Miocene evolution of alpine environments in the Himalayan-Tibetan Orogen

Joachim Schmidt^†^, Lars Opgenoorth, Kangshan Mao, Chitra B. Baniya, Sylvia Hofmann^†^

^†^These authors contributed equeally to this study.

**This PDF file includes:**

Supplementary text

Figures S1 to S3

Tables S1 to S7

SI References

Supplementary Information Text

**Ground beetles as paleoenvironmental proxies**

The beetle family Carabidae is one of the most species-diverse faunal groups with more than 40,000 described species worldwide (1). One of the global centers of Carabidae diversity is located in the Himalayan-Tibetan Orogen (HTO) (2). The majority of the species occurring in the HTO are locally endemic to certain massifs, mountain peaks, and valley systems. However, so far, only a single phylogeographic study addresses the evolution and biogeographic history of a ground beetle group from the HTO (3). This study indicates strict endemism of species in certain parts of the Himalayas and long isolation among populations of wingless ground beetles, with individual haplotypes on single mountain slopes and side valleys. Furthermore, winglessness of the species, together with strict preferences to specific humidity and temperature conditions, result in very poor dispersal ability and species-specific elevational limits, with deep valleys and dry slopes representing effective distributional barriers (4, 5). Consequently, the distribution of extant species and haplotypes of flightless ground beetle groups in the HTO allow robust conclusions about the local spatio-temporal history of the paleoenvironments in the course of the changing topography and climate of the HTO system (6-8).

Moreover, ground beetles are a phylogenetically old group of organisms that started to diversify at the beginning of the Cretaceous period (9, and references therein). Since modern genera of extratropical ground beetles of today's HTO are common in Paleogene fossil deposits of the pre-Palearctic region (10-12), it can be assumed that they colonized (and radiated in) the HTO as soon as it was uplifted to extratropical altitudes. The significant surface uplift in the HTO led to the development of high-altitude environments suitable for these ground beetles. Therefore, the uplift time – associated with the emergence of temperate habitats – should correspond to the first immigration of ancestral species that were pre-adapted to temperate climates. In response to the high-altitude conditions, most ground beetle lineages evolved towards winglessness, i.e., from strong dispersers (fully winged ancestral species) to very weak dispersers (flightless descendant species). The ability to fly was the fundamental premise for colonizing the HTO by ancestral lineages from the Cenozoic Boreal (4, 13). Subsequent development of winglessness triggered the evolution of endemic lineages and their geographic isolation in the course of the topographic changes of the HTO (3). Thus, deep phylogenic splits of endemic Himalayan ground beetle lineages can be highly informative on the environmental and uplift history of the mountain system.

One of the characteristic elements of the ground beetle fauna in the HTO is the mega-diverse Holarctic genus *Carabus*. Based on the latest revisions of the fossil data and secondary calibration points, the split of *Carabus* from its sister taxon *Calosoma* was estimated to the Late Cretaceous or early Paleogene (14, 15). The genus *Carabus* originated from the Cenozoic pre-Palearctic region (16). This taxon comprises almost 1,000 described extant species, all of which are distributed in the warm-temperate to subarctic zones, with the far most of them occurring in the Palearctic region and in the HTO, a hotspot of species diversity (2, 17). Most *Carabus* beetles are of large body size, flightless, but good runners on the ground surface of different habitats, including high mountains up to the alpine zone (18-20).

The *Carabus* fauna of the HTO shows a geographical distribution pattern that is strongly linked to mountain topography (Figures 5, 6). About 20 subgenera of *Carabus* are known to occur along the eastern Tibetan Plateau margin. None of these subgenera can be found in the Greater Himalaya, except for one (21): *Neoplesius* is the only Tibetan *Carabus* subgenus characterized by a trans-Tibetan distribution spanning from Qinghai in the north to the northern face of the Greater Himalaya in the south. The Himalayan *Carabus* fauna is strictly endemic. Notably, the species diverse subgenus *Imaibius* is distributed in the western Himalaya with by far the most species occurring in the Kashmir region and two local endemic species occurring in disjunct areas in western-central Nepal (17, 22; Figure 5). Further, species of the diverse lineage *Meganebrius* are distributed in a small part of the Greater Himalaya between the Dhaulagiri Himal in the West and the Kangchenjunga Himal in the East (17, 23; Figure 6). Two additional species, previously assigned to the subgenus *Meganebrius*, are found in far separated areas: the polytypic *Carabus scheibei* in the northern part of the Kashmir Himalaya, and the polytypic *C. alanstivelli* in the Far West of Nepal (Figure 5). Most strikingly, not a single *Carabus* taxon occurs in the eastern area of the Greater Himalaya east of Kangchenjunga. There is an enigmatic wide gap in the *Carabus* distribution between the transverse valleys of Tista River in the West and Brahmaputra in the East (Figure 5). The Central Himalaya hotspot of *Carabus* diversity ends abruptly on the southern face of Kangchenjunga Himal, while the above-mentioned East Tibet hotspot of *Carabus* diversity starts instantly east of the Brahmaputra transverse valley (17, 21). These gaps cannot be explained by the current ecological conditions in the area. Such distributional patterns cannot be found in plants, birds, butterflies, and other species groups with high dispersal power since their current distributions reflect more or less strongly the current climatic conditions in the HTO (overview in 24). Such marked distributional disjunctions are, however, a common phenomenon in HTO ground beetles, and spatio-temporal differences in the surface uplift and resulting paleoenvironments are hypothesized as the ultimate causes (3, 4, 13). Understanding the phylogeographic history of Himalayan-Tibetan *Carabus* by considering the ecological information gained from the species-specific habitat preferences and vertical distributions can offer insights into the spatio-temporal development of HTO paleoenvironments.

**SI References**

1. E. Arndt, R. G. Beutel, K. Will, "Carabidae Latreille, 1802" in Handbook of Zoology, Vol. IV, Arthropoda: Insecta Part 38 Coleoptera, Beetles*,* N. P. Kristensen, R. G. Beutel, Eds. (De Gruyter, Berlin, Ossining, 2005), pp. 119–146.

2. I. Löbl, D. Löbl, *Catalogue of Palearctic Coleoptera. Vol. 1. Archostemata-Myxophaga-Adephaga* (Brill, Leiden-Boston, ed. Revised and Updated Edition, 2017), pp. 1443.

3. J. Schmidt, L. Opgenoorth, S. Holl, R. Bastrop, Into the Himalayan exile: the phylogeography of the ground beetle *Ethira* clade supports the Tibetan origin of forest-dwelling Himalayan species groups. *PLoS One* 7, e45482 (2012).

4. J. Schmidt, Biogeographisch-phylogenetische Untersuchungen an Hochgebirgslaufkäfern zur Erkundung der Umweltgeschichte des Himalaya-Tibet Orogens. *Angewandte Carabidologie* 10, 41–66 (2013).

5. J. Schmidt, J. Bohner, R. Brandl, L. Opgenoorth, Mass elevation and lee effects markedly lift the elevational distribution of ground beetles in the Himalaya-Tibet orogen. *PLoS One* 12, e0172939 (2017).

6. F. Heberdey, Die Bedeutung der Eiszeit für die Fauna der Alpen. *Zoogeographica* 1, 353–412 (1933).

7. T. C. Atkinson, K. R. Briffa, G. R. Coope, Seasonal temperatures in Britain during the past 22,000 years, reconstructed using beetle remains. *Nature* 325, 587–592 (1987).

8. J. Schmidt, L. Opgenoorth, J. Martens, G. Miehe, Neoendemic ground beetles and private tree haplotypes: two independent proxies attest a moderate LGM summer temperature depression of 3 to 4K for the southern Tibetan Plateau. *Quaternary Sci. Rev.* 30, 1918–1925 (2011).

9. P. Brandmayr, An outlook on the evolutionary history of Carabidae (Coleoptera, Adephaga). *Memorie della Società Entomologica Italiana* 97, 15–46 (2021).

10. J. Schmidt, I. Belousov, P. Michalik, X-ray microscopy reveals endophallic structures in a new species of the ground beetle genus *Trechus* Clairville, 1806 from Baltic amber (Coleoptera, Carabidae, Trechini). *Zookeys* 614, 113–127 (2016).

11. J. Schmidt, P. Michalik, The ground beetle genus Bembidion Latreille in Baltic amber: Review of preserved specimens and first 3D reconstruction of endophallic structures using X-ray microscopy (Coleoptera, Carabidae, Bembidiini). *Zookeys* 662, 101–126 (2017).

12. V. M. Ortuño, A. Arillo, Fossil carabids from Baltic amber – I – A new species of the genus *Calathus* Bonelli, 1810 (Coleoptera: Carabidae: Pterostichinae). *Zootaxa* 2239, 55–61 (2009).

13. J. Martens, "Fauna – Himalayan patterns of diversity" in Nepal. An introduction to the natural history, ecology and human environment in the Himalayas. A companion to the Flora of Nepal*,* G. Miehe, C. Pendry, Eds. (Royal Botanic Garden Edinburgh, Edinburgh, UK, 2015), pp. 168–173.

14. L. Opgenoorth, S. Hofmann, J. Schmidt, Rewinding the molecular clock in the genus *Carabus* (Coleoptera: Carabidae) in light of fossil evidence and the Gondwana split: A reanalysis. *PLoS One* 16, e0256679 (2021).

15. E. F. A. Toussaint *et al.*, HyRAD-X Exome Capture Museomics Unravels Giant Ground Beetle Evolution. *Genome Biol. Evol.* 13, evab112 (2021).

16. T. Deuve, A. Cruaud, G. Genson, J. Y. Rasplus, Molecular systematics and evolutionary history of the genus Carabus (Col. Carabidae). *Mol. Phylogenet. Evol.* 65, 259–275 (2012).

17. T. Deuve, *Illustrated Catalogue of the Genus Carabus of the World* (Pensoft Publishers, 2004).

18. C. H. Lindroth, Die fennoskandischen Carabidae. I. Spezieller Teil. *Goteborgs Kungliga Vetenskaps och Vitter Hets-Samhalles Handlingar Sjatte Foljden. Ser. B* 4, 1–709 (1945).

19. H. Turin, L. D. Penev, A. Casale, *The Genus Carabus in Europe. A Synthesis* (Pensoft, Sofia-Moscow, 2003).

20. Y. Imura *et al.*, Evolutionary history of carabid ground beetles with special reference to morphological variations of the hind-wings. *Proc. Jpn. Acad. Ser. B Phys. Biol. Sci.* 94, 360–371 (2018).

21. T. Deuve, *Cychrus, Calosoma et Carabus de Chíne* (Pensoft, 2013).

22. T. Deuve, J. Schmidt, Description d’un nouvel *Imaibius* du Népal (Coleoptera, Carabidae). *Revue Francaise d’Entomologie (N.S.)* 29, 11–14 (2007).

23. T. Deuve, J. Schmidt, Deux nouvelles espèces de *Meganebrius* Kraatz, 1895, du Népal oriental (Coleoptera, Carabidae). *L’Entomologiste* 73, 53–59 (2017).

24. G. Miehe, C. Pendry, *Nepal. An introduction to the natural history, ecology and human environment in the Himalayas. A companion to the Flora of Nepal* (Royal Botanic Garden Edinburgh, Edinburgh, UK, 2015).


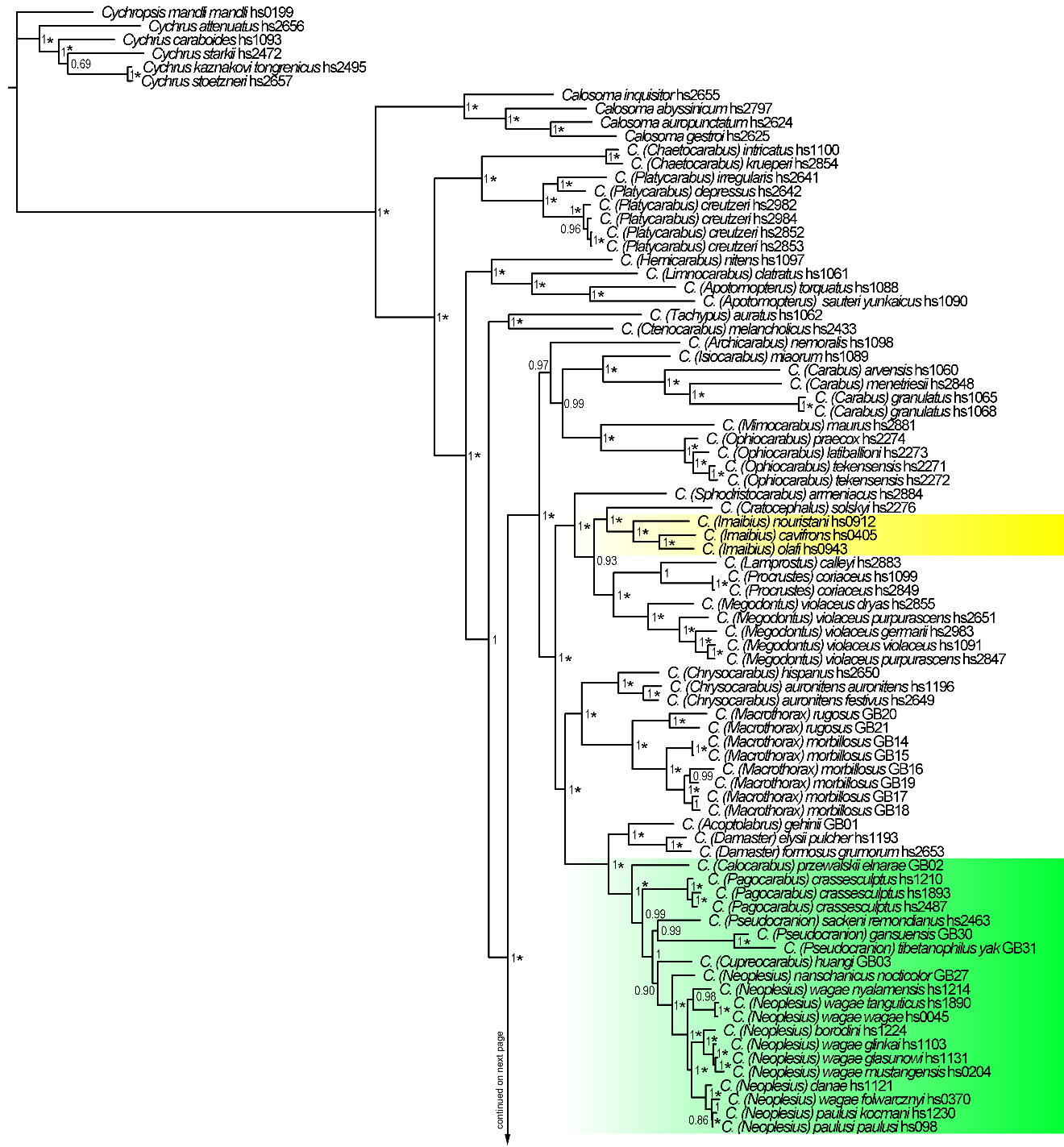


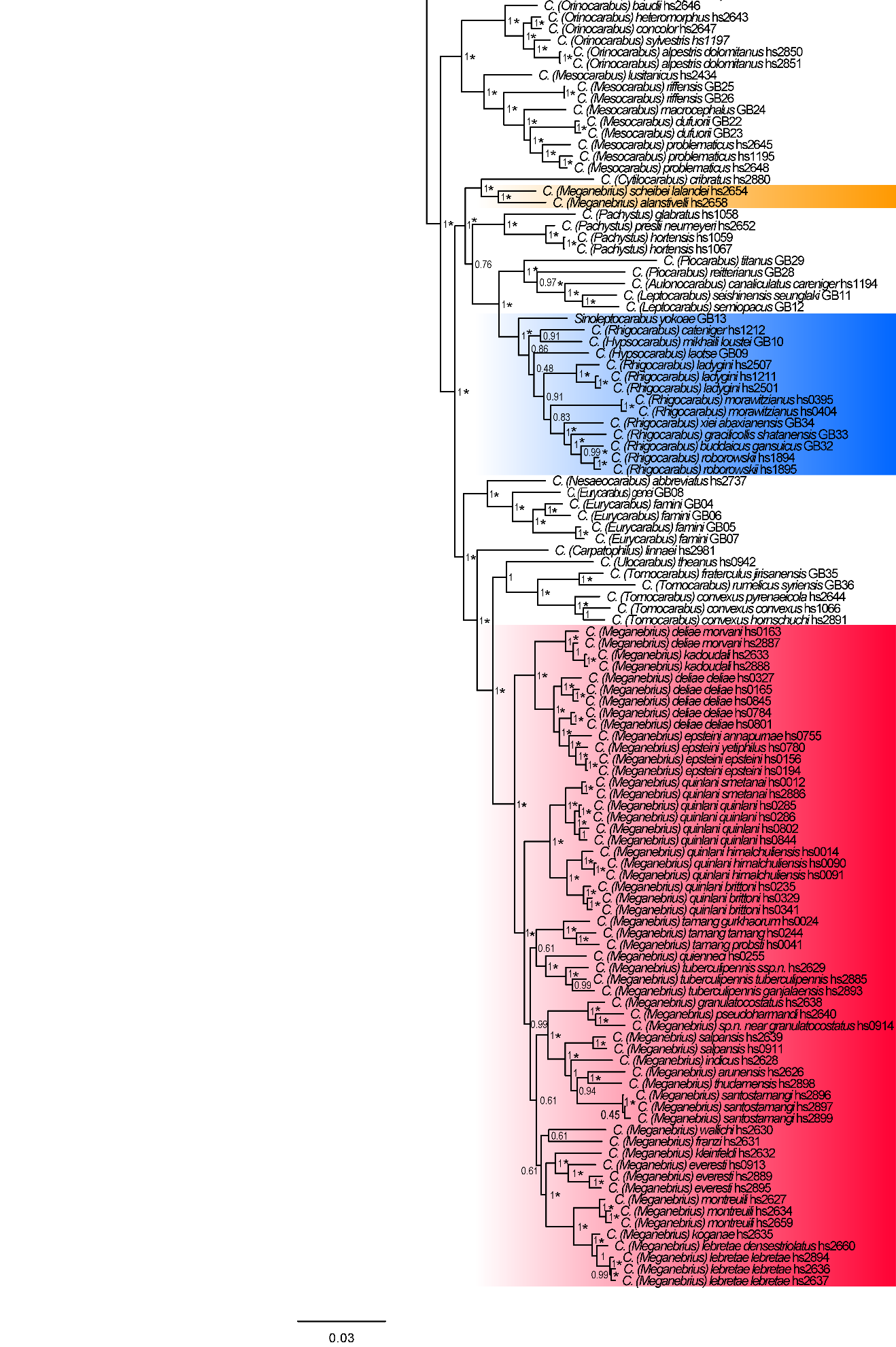


Figure S1. Consensus tree inferred with MrBayes based on the concatenated sequence data of *Carabus* beetles and outgroups. Lineages endemic to certain parts of the HTO are highlighted by the same color code as in Figure 2. Black rectangles and stars at branch nodes refer to posterior probabilities ≥0.98 and bootstrap values >70.0, respectively.


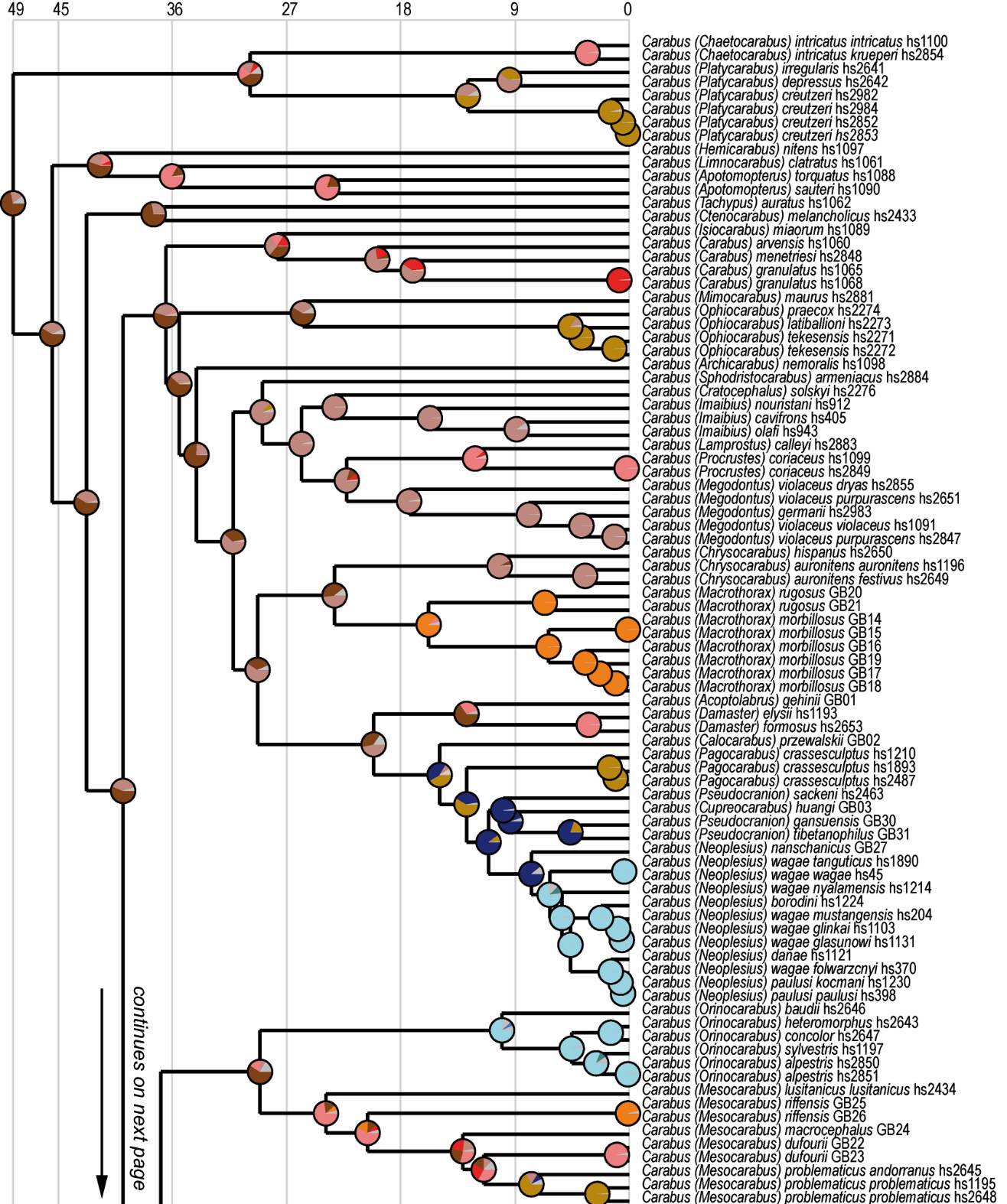


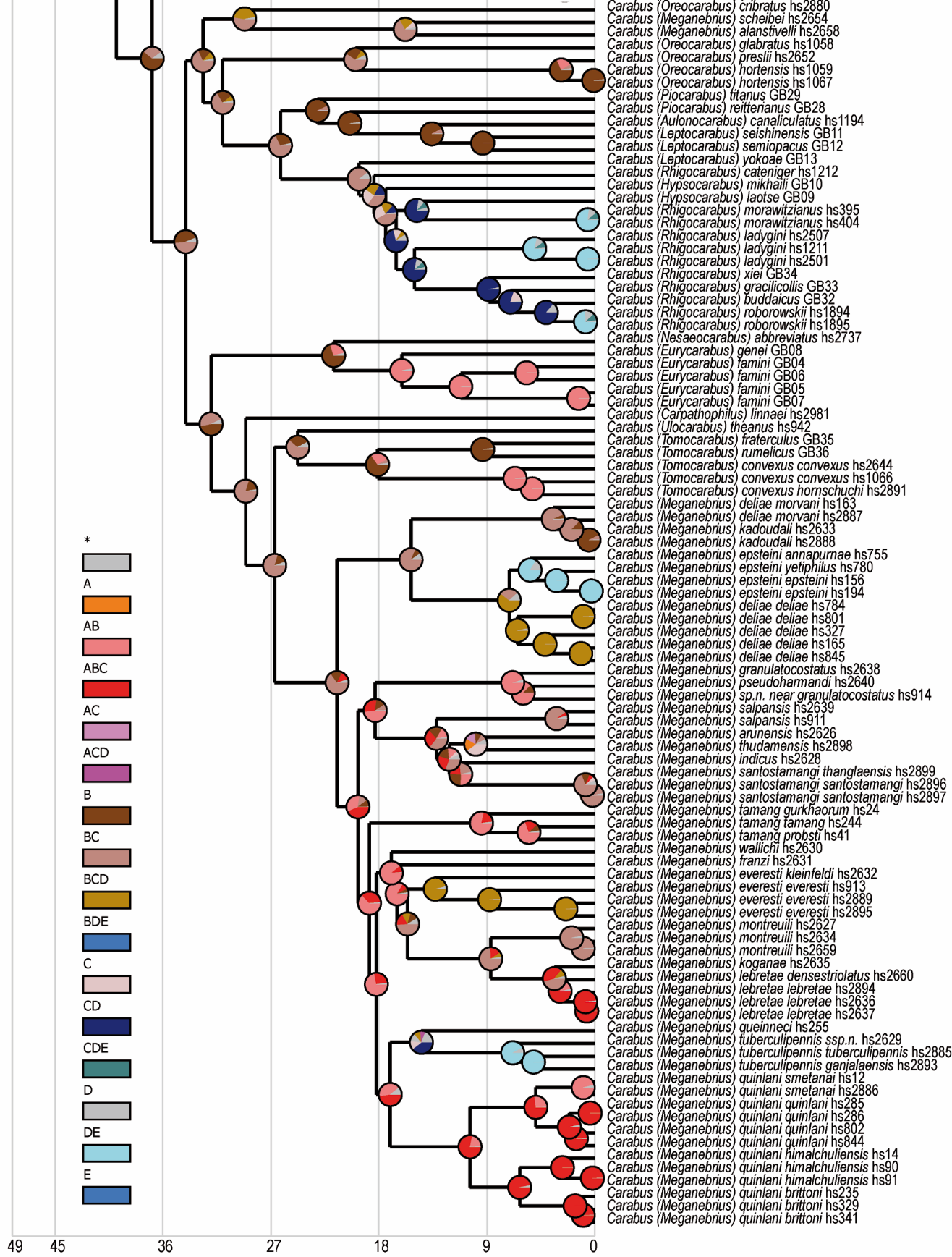


Figure S2. Ancestral state estimation based on the BBM method in RASP. Pie charts on each node of the tree indicate marginal probabilities for each alternative ancestral habitat. Letters and colors in the legend refer to extant and possible ancestral habitats, and combinations of these: A, warm temperate; B, temperate; C, cold temperate; D, subarctic/subalpine; E, arctic/alpine.


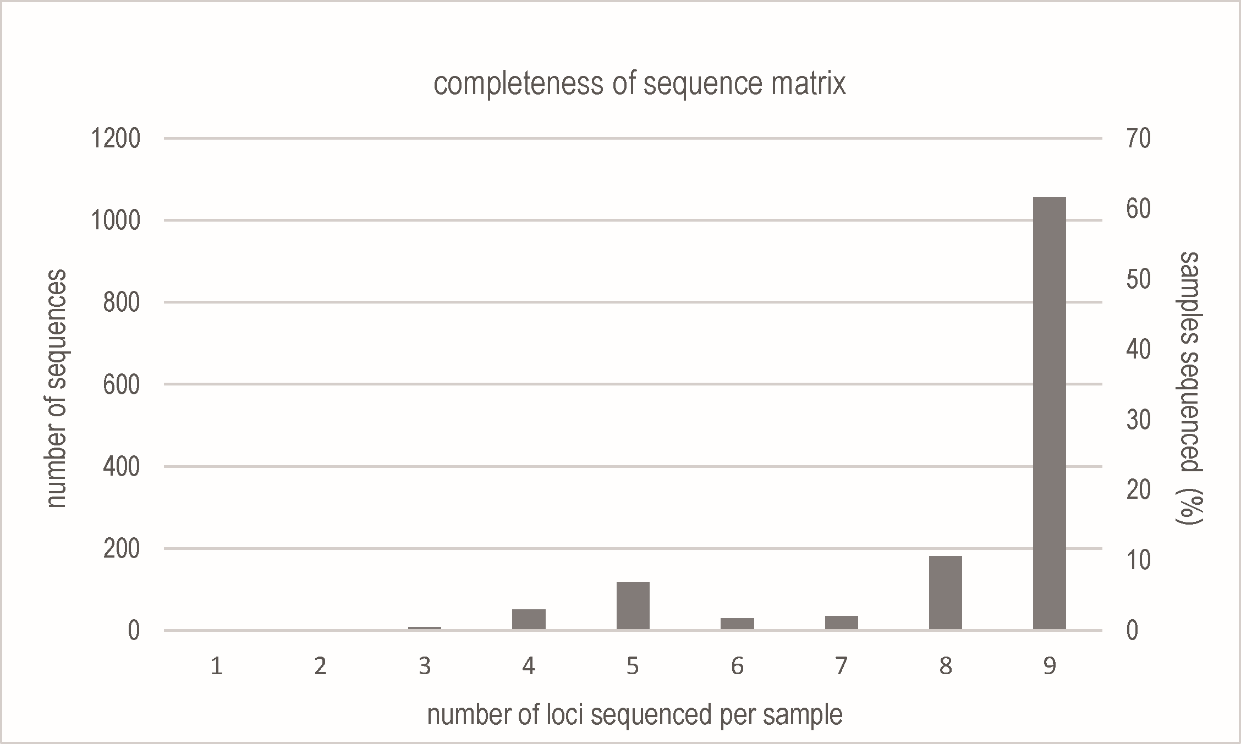


Figure S3. Summary of Supplementary Table S3. Distribution of sequenced samples over the number of loci.

Table S1. Eocene to Miocene fossil data from the Himalaya-Tibet Orogen and associated paleoelevational belts.

| Age (Ma) | Locality | Paleoelevation | Paleoelevational belt | Proxy | Method | Source |
| --- | --- | --- | --- | --- | --- | --- |
| 40-37 | Hoh Xil basin | < 2000 m | subtropical, dry | pollen record | comparison with recent floras | [1] |
| 39.5 (previously dated to 26-23.5) | Lunpola basin | ~ 1000 m | tropic-subtropic | fossil fishes and flora | comparison with recent fauna and floras | [2, 3] |
| 39.5 (previously dated to 26-23.5) | Lunpola basin | < 2300 m | tropic-subtropic | fossil palm leaves | CLAMP | [3, 4] |
| 38 | Xining basin | “high” | warm temperate | pollen record | comparison with recent floras | [5] |
| 35 | Markam basin | 2640–2900 m | subtropical to warm temperate | fossil forest flora | CLAMP | [6] |
| 34.7-33.4 | Markam basin | 3085 ± 910 m | temperate | fossil forest flora | CLAMP | [6] |
| 30.8 | Qaidam basin | 3300 ± 1400 m | temperate | fossil forest flora | CLAMP | [7] |
| 26-23.5 | Nima basin | ~ 1000 m | tropic-subtropic | fossil fishes and flora | comparison with recent fauna and flora | [2] |
| 25.5-19.8 | Lunpola basin | max. 3190 ± 100 m | tropic to temperate | pollen record | CA | [8] |
| 23.3 | Kailas basin | 1500–2900 m | warm temperate | fossil forest flora | CA | [9] |
| 22 | Shuanghe Formation at Jianchuan | - | subtropic | fossil forest flora | CA, LMA | [10] |
| 21-19 | Qiabulin basin | 2300 +/- 900 m | warm temperate | fossil forest flora | CLAMP | [11] |
| 15 | Namling-Oiyug basin | 3791–5584 m | cold temperate | fossil forest flora | CLAMP | [12] |
| 15 | Namling-Oiyug basin | 2500–3000 m | cold temperate | fossil forest flora | CA | [13] |
| 15 | Namling-Oiyug basin | ~ 5400 m | cold temperate | fossil forest flora | CLAMP | [14] |
| 10,8-7,2 | Gyirong Basin: Danzengzhukang Formation | < 3000 m | subtropical to warm temperate | palynological data, *Hipparion* fauna, C4 grasses | review multiple fossil data | [15] |
| 7,2-3,2 | Gyirong Basin: lower Woma formation | variable topography: 2000–4500 m | warm temperate to subalpine | palynological data, ostracod fauna | review multiple fossil data | [15] |

[1] Y. Miao, et al., A Late-Eocene palynological record from the Hoh Xil Basin, northern Tibetan Plateau, and its implications for stratigraphic age, paleoclimate and paleoelevation. *Gondwana Res.* 31, 241–252 (2016).

[2] F. Wu, Fossil climbing perch and associated plant megafossils indicate a warm and wet central Tibet during the late Oligocene. *Sci. Rep.* 7, 878 (2017).

[3] X. Fang, X. Revised chronology of central Tibet uplift (Lunpola Basin). *Sci. Adv.* 6, eaba7298 (2020).

[4] T. Su, et al., No high Tibetan Plateau until the Neogene. *Sci. Adv.* 5, eaav2189 (2019).

[5] G. Dupont-Nivet, C. Hoorn, M. Konert, Tibetan uplift prior to the Eocene-Oligocene climate transition: Evidence from pollen analysis of the Xining Basin. *Geology* 36, 987–990 (2008).

[6] T. Su, et al., Uplift, climate and biotic changes at the Eocene–Oligocene transition in south-eastern Tibet. *National Sci. Rev.* 6, 495–504 (2018).

[7] B. Song, Qaidam Basin leaf fossils show northeastern Tibet was high, wet and cool in the early Oligocene. *Earth Planet. Sci. Lett.* 537, 116175 (2020).

[8] J. Sun, et al., Palynological evidence for the latest Oligocene−early Miocene paleoelevation estimate in the Lunpola Basin, central Tibet. *Palaeogeogr. Palaeoclimatol. Palaeoecol.* 399, 21–30 (2014).

[9] K. Ai, et al., The uppermost Oligocene Kailas flora from southern Tibetan Plateau and its implications for the uplift history of the southern Lhasa terrane. *Palaeogeogr. Palaeoclimatol. Palaeoecol.* 515, 143–151 (2019).

[10] B. N. Sun, et al., Reconstructing Neogene vegetation and climates to infer tectonic uplift in western Yunnan, China. *Palaeogeogr. Palaeoclimatol. Palaeoecol.* 304, 328–336 (2011).

[11] L. Ding, et al., Quantifying the rise of the Himalaya orogen and implications for the South Asian monsoon. *Geology*, 45, 215–218 (2017).

[12] R. A. Spicer, et al., Constant elevation of southern Tibet over the past 15 million years. *Nature* 421, 622–624 (2003).

[13] Z. Zhou, Q. Yang, K. Xia, Fossils of *Quercus* sect. *Heterobalanus* can help explain the uplift of the Himalayas. *Chinese Sci. Bull.* 52, 238–247 (2007).

[14] M. A. Khan, et al., Miocene to Pleistocene floras and climate of the Eastern Himalayan Siwaliks, and new palaeoelevation estimates for the Namling–Oiyug Basin, Tibet. *Glob. Planet. Change* 113, 1–10 (2014).

[15] Y.-D. Xu, et al., Extended stratigraphy, palynology and depositional environments record the initiation of the Himalayan Gyirong Basin (Neogene China). *J. Asian Earth Sci.* 44, 77–93 (2012).

Table S2. List of *Carabus* and outgroup species used in this study with taxonomic information, sampling localities and voucher numbers.

|  | Locality data | coordinates | ID |
| --- | --- | --- | --- |
| *Calosoma (Calosoma) inquisitor* (L.) | GREECE Kefallinia, Sami, alt. 50 m | 38°15'N 20°39'E | hs2655 |
| *Calosoma (Campalita) maderae auropunctatum* (Herbst) | GERMANY Mecklenburg, Boizenburg, Nostorf, alt. 10 m | 53°25'N 10°36'E | hs2624 |
| *Calosoma (Carabomorphus) gestroi* Breuning | ETHIOPIA Bale Mts., Fincha Habera, alt. 3500 m | 07°00'N 39°43'E | hs2625 |
| *Calosoma (Carabops) abyssinicum* Gestro | ETHIOPIA Oromia, Mt. Kaka, alt. 3420 m | 07°24'N 39°12'E | hs2797 |
| *Carabus (Apotomopterus) sauteri yunkaicus* Deuve | CHINA Guangxi, Maoer Shan, alt. 900 m | 25°52'N 110°29'E | hs1090 |
| *Carabus (Apotomopterus) t.orquatus torquatus* Cavazzuti | CHINA Guangxi, Maoer Shan, alt. 900 m | 25°52'N 110°29'E | hs1088 |
| *Carabus (Archicarabus) nemoralis* Müller | GERMANY Mecklenburg, Admannshagen, alt. 8 m | 54°08'N 11°59'E | hs1098 |
| *Carabus (Aulonocarabus) canaliculatus* *careniger* Chaudoir | CHINA Hebei, Wuling Shan, alt. 1600 m | 40°35'N 117°29'E | hs1194 |
| *Carabus (Carabus) arvensis* Herbst | GERMANY Mecklenburg, Göldenitzer Moor, alt. 40 m | 54°00'N 12°19'E | hs1060 |
| *Carabus (Carabus) granulatus* L. | GERMANY Mecklenburg, Admannshagen, alt. 8 m | 54°08'N 11°59'E | hs1065 |
| *Carabus (Carabus) granulatus* L. | GERMANY Mecklenburg, Rostock, Rosenort, alt. 1 m | 54°14'N 12°11'E | hs1068 |
| *Carabus (Carabus) menetriesi* Faldermann | GERMANY Bavaria, NP Bayerischer Wald, Spiegelau, Gr. Filz, alt. 757 m | 48°55'N 13°24'E | hs2848 |
| *Carabus (Carpatophilus) linnaei* Panzer | ITALY Trentino, Passo Rolle S-slope, alt. 1700 m | 46°17'N 11°48'E | hs2981 |
| *Carabus (Chaetocarabus) intricatus intricatus* L. | GERMANY, Biosphärenreservat Pfälzerwald, „Schmaler Hals“, alt. 550 m | 49°14'N 07°48'E | hs1100 |
| *Carabus (Chaetocarabus) intricatus krueperi* Reitter | GREECE Pieria, Ano Milia, alt. 1200 m | 40°14'N 22°14'E | hs2854 |
| *Carabus (Chrysocarabus) auronitens auronitens* Fabricius | GERMANY Saxonia, Schwarzwasser Valley, Kühnhaide to Pobershau, alt. 670 m | 50°37'N 13°14'E | hs1196 |
| *Carabus (Chrysocarabus) auronitens festivus* Dejean | FRANCE Tarn, Montagne Noire, SW of Mazamet, alt. 900 m | 43°28'N 02°14'E | hs2649 |
| *Carabus (Chrysocarabus) hispanus* Fabricius | FRANCE Cévennes, Lozère, N of Mont Aigoual, alt. 1150 m | 44°11'N 03°31'E | hs2650 |
| *Carabus (Cratocephalus) solskyi* Ballion | CHINA Xinjiang, Narat Mt. Range, Erbotu Valley, alt. 1915 m | 43°06'N 83°00'E | hs2276 |
| *Carabus (Ctenocarabus) melancholicus* Fabricius | SPAIN Sierra de Gredos, valley above Gavilanes, alt. 1100 m | 40°16'N 04°51'W | hs2433 |
| *Carabus (Cytilocarabus) cribratus* Quensel | ARMENIA Aragatsotn, Mt. Aragatsotn, alt. 2700 m | 40°30'N 44°15'E | hs2880 |
| *Carabus (Damaster) elysii pulcher* Kleinfeld | CHINA Henan Prov., Jigong Shan, 700 m | 31°81'N 114°08'E | hs1193 |
| *Carabus (Damaster)* *formosus* *grumorum* Semenov | CHINA Sichuan, Ganze, alt. 3460 m | 31°40'N 99°44'E | hs2653 |
| *Carabus (Hemicarabus) nitens* L. | GERMANY Mecklenburg, Göldenitzer Moor, alt. 40 m | 54°00'N 12°19'E | hs1097 |
| *Carabus (Imaibius) cavifrons* Mandl | NEPAL Jumla, Gothichaur Valley, alt. 2900 m | 29°10'N 82°22'E | hs0405 |
| *Carabus (Imaibius) nouristani* Ledoux | AFGHANISTAN Pashinkanda, Darah-I-Nur, 40 km NE Jalalabad, alt. 3000 m | 34°45'N 70°40'E | hs0912 |
| *Carabus (Imaibius) olafi* Deuve & Schmidt | NEPAL Myagdi, Baglung Lekh, 10 km W Baglung alt. 2400 m | 28°19'N 83°32'E | hs0943 |
| *Carabus (Isiocarabus) miaorum* Lassalle & Prunier | CHINA Guangxi, Maoer Shan, alt. 900 m | 25°52'N 110°29'E | hs1089 |
| *Carabus (Lamprostus) calleyi* Fischer | ARMENIA Vayots Dzor, W-slope Vorotan-Pass, alt. 2250 m | 39°41'N 45°42'E | hs2883 |
| *Carabus (Limnocarabus) clatratus* L. | GERMANY Mecklenburg, Göldenitzer Moor, alt. 40 m | 54°00'N 12°19'E | hs1061 |
| *Carabus (Meganebrius) alanstivelli* Morvan | NEPAL Bahjura, 44 km NE Chainpur, alt. 3900 m | 29°48'N 81°30'E | hs2658 |
| *Carabus (Meganebrius) arunensis* Heinertz | NEPAL, Arun Valley, Sankhuwasabha, Chichila, alt. 1900 m | 27°28'N 87°14'E | hs2626 |
| *Carabus (Meganebrius) deliae deliae* Morvan | NEPAL S-slope Dhaulagiri Himal, Lete Pass, alt. 4150m | 28°35'N 83°35'E | hs0784 |
| *Carabus (Meganebrius) deliae deliae* Morvan | NEPAL Kali Gandaki Vall. to Nilgiri Himal, above Sauru, alt. 3650 m | 28°40'N 83°39'E | hs0801 |
| *Carabus (Meganebrius) deliae deliae* Morvan | NEPAL S-slope Annapurna South Himal, Ghorepani, alt. 2800 m | 28°24'N 83°42'E | hs0165 |
| *Carabus (Meganebrius) deliae deliae* Morvan | NEPAL S-slope Annapurna South Himal, NW Tadapani, alt. 3300 m | 28°24'N 83°45'E | hs0845 |
| *Carabus (Meganebrius) deliae deliae* Morvan | NEPAL SE-slope Lamjung Himal, below Sundar Danda alt. 3400 m | 28°24'N 84°21'E | hs0327 |
| *Carabus (Meganebrius) deliae morvani* Lassalle | NEPAL SW-slope Dhaulagiri Himal, E-slope of Jaljala Pass, alt. 3000 m | 28°31'N 83°15'E | hs2887 |
| *Carabus (Meganebrius) deliae morvani* Lassalle | NEPAL Myagdi, Baglung Lekh, 30 km W Baglung, alt. 2900 m | 28°22'N 83°20'E | hs0163 |
| *Carabus (Meganebrius) epsteini annapurnae* Deuve & Schmidt | NEPAL S-slope of Annapurna South Himal, above Khopra, alt. 4100 m | 28°29'N 83°44'E | hs0755 |
| *Carabus (Meganebrius) epsteini epsteini* Heinertz | NEPAL Kali Gandaki Valley, Yakkharka above Marpha, alt. 3750 m | 28°45'N 83°39'E | hs0194 |
| *Carabus (Meganebrius) epsteini epsteini* Heinertz | NEPAL Kali Gandaki Vall. to Nilgiri South Himal, Thulo Bugin, alt. 4000 m | 28°37'N 83°41'E | hs0156 |
| *Carabus (Meganebrius) epsteini yetiphilus* Deuve & Schmidt | NEPAL S-slope of Dhaulagiri Himal, Lete Pass, alt. 4150m | 28°35'N 83°35'E | hs0780 |
| *Carabus (Meganebrius) everesti everesti* Andrewes | NEPAL Bhojpur, Bilbate E of Salpa Pass, alt. 2800 m | 27°25'N 86°57'E | hs0913 |
| *Carabus (Meganebrius) everesti everesti* Andrewes | NEPAL Solu Khumbu, Taktor W Junbesi, alt. 3100 m | 27°35'N 86°31'E | hs2889 |
| *Carabus (Meganebrius) everesti everesti* Andrewes | NEPAL Dolakha, NW Jiri, alt. 2860 m | 27°41'N 86°14'E | hs2895 |
| *Carabus (Meganebrius) everesti kleinfeldi* Korell | NEPAL Helambu, Paanch Pokhari Lekh, Hile Kharka, alt. 3600 m | 27°59'N 85°44'E | hs2632 |
| *Carabus (Meganebrius) franzi* Mandl | NEPAL Helambu, Paanch Pokhari Lekh, above Kamikharka, alt. 3000 m | 27°53'N 85°42'E | hs2631 |
| *Carabus (Meganebrius) granulatocostatus* Mandl | NEPAL, Solu Khumbu, below Najing, alt. 2650 m | 27°34'N 86°48'E | hs2638 |
| *Carabus (Meganebrius) indicus* Fairmaire | NEPAL, Taplejung Distr., Panduwa Bhanjyang, alt. 3720 m | 27°23'N 88°01'E | hs2628 |
| *Carabus (Meganebrius) kadoudali* Morvan | NEPAL W-slope Dhaulagiri Himal, Thankur, alt. 3250 m | 28°36'N 83°01'E | hs2888 |
| *Carabus (Meganebrius) kadoudali* Morvan | NEPAL S-slope Dhaulagiri Himal, W-slope of Jaljala Pass, alt. 3400 m | 28°31'N 83°13'E | hs2633 |
| *Carabus (Meganebrius) koganae* Colas | NEPAL Solu Khumbu, S Dudh Kund, alt. 4300 m | 27°41'N 86°35'E | hs2635 |
| *Carabus (Meganebrius) lebretae densestriolatus* Mandl | NEPAL Dolakha, Tingsang La, alt. 3030 m | 27°49'N 86°00'E | hs2660 |
| *Carabus (Meganebrius) lebretae lebretae* Colas | NEPAL Solu Khumbu, Taktor W Junbesi, alt. 3100 m | 27°35'N 86°31'E | hs2636 |
| *Carabus (Meganebrius) lebretae lebretae* Colas | NEPAL, Ramechhap, between Likhu Khola and Khimti Khola, alt. 2800 m | 27°35'N 86°20'E | hs2637 |
| *Carabus (Meganebrius) lebretae lebretae* Colas | NEPAL Dolakha, upper Khimti Khola Vall. alt. 3180m | 27°42'N 86°20'E | hs2894 |
| *Carabus (Meganebrius) montreuili* Deuve | NEPAL Solu Khumbu, Surkie La, alt. 3000 m | 27°34'N 86°49'E | hs2634 |
| *Carabus (Meganebrius) montreuili* Deuve | NEPAL Solu Khumbu, Majang Khola, alt. 3580 m | 27°38'N 86°49'E | hs2659 |
| *Carabus (Meganebrius) montreuili* Deuve | NEPAL Sankhuwasabha, Arun Vall. to Makalu, Dhara Kharka, alt. 2935 m | 27°38'N 87°13'E | hs2627 |
| *Carabus (Meganebrius) pseudoharmandi* Mandl | NEPAL Sankhuwasabha, Arun Valley to Makalu, Nagala Kharka, alt. 2530 m | 27°40'N 87°18'E | hs2640 |
| *Carabus (Meganebrius) queinneci* Deuve | NEPAL Ganesh Himal, Pansang Danda, alt. 3820 m | 28°09'N 85°10'E | hs0255 |
| *Carabus (Meganebrius) quinlani himalchuliensis* Lassalle | NEPAL S-slope Manaslu Himal, Dudh Pokhari Lekh, alt. 2900 m | 28°18'N 84°35'E | hs0090 |
| *Carabus (Meganebrius) quinlani himalchuliensis* Lassalle | NEPAL S-slope Manaslu Himal, Dudh Pokhari Lekh, alt. 2900 m | 28°18'N 84°35'E | hs0091 |
| *Carabus (Meganebrius) quinlani himalchuliensis* Lassalle | NEPAL S-slope Manaslu Himal, Bara Pokhari, alt. 3100 m | 28°18'N 84°28'E | hs0014 |
| *Carabus (Meganebrius) quinlani brittoni* Mandl | NEPAL N-slope Lamjung Himal, Temang, alt. 2670 m | 28°32'N 84°18'E | hs0235 |
| *Carabus (Meganebrius) quinlani brittoni* Mandl | NEPAL SE-slope Lamjung Himal, below Sundar Danda alt. 2400m | 28°22'N 84°22'E | hs0341 |
| *Carabus (Meganebrius) quinlani brittoni* Mandl | NEPAL SE-slope Lamjung Himal, below Sundar Danda alt. 3400 m | 28°24'N 84°21'E | hs0329 |
| *Carabus (Meganebrius) quinlani quinlani* Mandl | NEPAL S-slope Annapurna II Himal, Sikles, alt. 2050 m | 28°21'N 84°06'E | hs0285 |
| *Carabus (Meganebrius) quinlani quinlani* Mandl | NEPAL SW-slope Lamjung Himal, Bachhar Kharka, alt. 2380 m | 28°22'N 84°08'E | hs0286 |
| *Carabus (Meganebrius) quinlani quinlani* Mandl | NEPAL S-slope Annapurna South Himal, NW Tadapani, alt. 3300 m | 28°24'N 83°45'E | hs0844 |
| *Carabus (Meganebrius) quinlani quinlani* Mandl | NEPAL Kali Gandaki Vall. to Nilgiri Himal, above Sauru, alt. 3650 m | 28°40'N 83°39'E | hs0802 |
| *Carabus (Meganebrius) quinlani smetanai* Häckel & Brezina | NEPAL Myagdi, Baglung Lekh, above Okhle, alt. 2450 m | 28°18'N 83°29'E | hs0012 |
| *Carabus (Meganebrius) quinlani smetanai* Häckel & Brezina | NEPAL SW-slope Dhaulagiri Himal, Dhorpatan, alt. 2900 m | 28°29'N 83°06'E | hs2886 |
| *Carabus (Meganebrius) salpansis* Deuve | NEPAL Bhojpur, Lawari Kharka alt. 3250 m | 27°23'N 86°55'E | hs0911 |
| *Carabus (Meganebrius) salpansis* Deuve | NEPAL Sankhuwasabha, Arun Valley to Makalu, Bawala Pass, alt. 4200 m | 27°43'N 87°15'E | hs2639 |
| *Carabus (Meganebrius) santostamangi santostamangi* Deuve & Schmidt | NEPAL Taplejung, Palung Khola, alt. 3100 m | 27°35'N 87°41'E | hs2896 |
| *Carabus (Meganebrius) santostamangi santostamangi* Deuve & Schmidt | NEPAL Taplejung, E-slope Palung Khola Vall., alt. 3720 m | 27°36'N 87°42'E | hs2897 |
| *Carabus (Meganebrius) santostamangi thanglaensis* Deuve & Schmidt | NEPAL Sankhuawasabha/Taplejung, Thangla Phedi alt. 4150 m | 27°39'N 87°36'E | hs2899 |
| *Carabus (Meganebrius) scheibei* Eidam | PAKISTAN Dir, Hindukush, Lawarai Pass, alt. 3200 m | 35°21'N 71°48'E | hs2654 |
| *Carabus (Meganebrius)* sp.n. near *granulatocostatus* | NEPAL Bhojpur, Bilbate E of Salpa Pass, alt. 2800 m | 27°25'N 86°57'E | hs0914 |
| *Carabus (Meganebrius) tamang gurkhaorum* Lassalle | NEPAL S-slope Manaslu Himal, south below Bara Pokhari, alt. 2200 m | 28°16'N 84°26'E | hs0024 |
| *Carabus (Meganebrius) tamang probsti* Deuve | NEPAL NE-slope Manaslu Himal, above Prok, alt. 2950 m | 28°31'N 84°50'E | hs0041 |
| *Carabus (Meganebrius) tamang tamang* Deuve | NEPAL Ganesh Himal, Pansang Danda, alt. 3820 m | 28°09'N 85°10'E | hs0244 |
| *Carabus (Meganebrius) thudamensis* Deuve & Schmidt | NEPAL Sankhuawasabha, Thudam, alt. 3860 m | 27°45'N 87°33'E | hs2898 |
| *Carabus (Meganebrius) tuberculipennis ganjalaensis* Deuve | NEPAL Helambu, near Kanja La pass, alt. 4500 m | 28°06'N 85°34'E | hs2893 |
| *Carabus (Meganebrius) tuberculipennis* ssp.n. | NEPAL Helambu, Paanch Pokhari, alt. 4100 m | 28°03'N 85°43'E | hs2629 |
| *Carabus (Meganebrius) tuberculipennis tuberculipennis* Mandl | NEPAL Helambu, S Gosainkund, Gyaje Kharka, 3900 m | 28°04'N 85°27'E | hs2885 |
| *Carabus (Meganebrius) wallichi* Hope | NEPAL Helambu, Paanch Pokhari Lekh, Dshungel Camp, alt. 2800 m | 27°52'N 85°42'E | hs2630 |
| *Carabus (Megodontus) germarii* Sturm | ITALIA Trentino, above Passo Rolle, 2070 m | 46°18'N 11°49'E | hs2983 |
| *Carabus (Megodontus) violaceus dryas* Gistel | GREECE above Pieria, alt. 1800 m | 40°16'N 22°12'E | hs2855 |
| *Carabus (Megodontus) violaceus purpurascens* Fabricius | UK, Devon, Dartmoor, Dendles Wood, alt. 220 m | 50°26'N 03°57'W | hs2847 |
| *Carabus (Megodontus) violaceus purpurascens* Fabricius | FRANCE Hautes-Pyrénées, Col d'Aspin, alt. 1500 m | 42°56'N 00°19'E | hs2651 |
| *Carabus (Megodontus) violaceus violaceus* L. | GERMANY Mecklenburg, Rostock, Rosenort, alt. 1 m | 54°14'N 12°11'E | hs1091 |
| *Carabus (Mesocarabus) lusitanicus lusitanicus* Fabricius | SPAIN Sierra de Guadarrama, E Puerto de los Cotos, alt. 1700 m | 40°50'N 03°57'W | hs2434 |
| *Carabus (Mesocarabus) problematicus andorranus* Lapouge | ANDORRA Port d'Envalira, alt. 2400 m | 42°32'N 01°43'E | hs2645 |
| *Carabus (Mesocarabus) problematicus problematicus* Herbst | GERMANY Thüringen, Schellroda, alt. 450 m | 50°55'N 11°06'E | hs1195 |
| *Carabus (Mesocarabus) problematicus problematicus* Herbst | FRANCE Cévennes, Lozère, N of Mont Aigoual, alt. 1150 m | 44°11'N 03°31'E | hs2648 |
| *Carabus (Mimocarabus) maurus* (Adams) | ARMENIA Aragatsotn, Mt. Aragatsotn, alt. 2700 m | 40°30'N 44°15'E | hs2881 |
| *Carabus (Neoplesius) borodini* Heinz | CHINA South Tibet, Gangdise Shan, above Mila, alt. 5100 m | 29°50'N 92°21'E | hs1224 |
| *Carabus (Neoplesius) danae* Kaláb | CHINA South Tibet, Gangdise Shan W Lhasa, Chüsül Valley, alt. 4300 m | 29°30'N 90°41'E | hs1121 |
| *Carabus (Neoplesius) paulusi kocmani* Kaláb | CHINA South Tibet, Gangdise Shan, N Menpa Monastery, alt. 5300 m | 30°08'N 92°12'E | hs1230 |
| *Carabus (Neoplesius) paulusi paulusi* Kaláb | CHINA South Tibet, Gangdise Shan NE Lhasa, Chak La, alt. 5000 m | 30°07'N 91°16'E | hs0398 |
| *Carabus (Neoplesius) wagae folwarzcnyi* Deuve | CHINA South Tibet, Nyainqentanglha Shan, Shogu Tsu Vall., alt. 4700 m | 29°56'N 90°14'E | hs0370 |
| *Carabus (Neoplesius) wagae glasunowi* Heinz | CHINA South Tibet, Tibetan Himalaya, Karola, alt. 5000 m | 28°54'N 90°09'E | hs1131 |
| *Carabus (Neoplesius) wagae glinkai* Heinz | CHINA South Tibet, Tibetan Himalaya, Kampa La, alt. 4800 m | 29°12'N 90°37'E | hs1103 |
| *Carabus (Neoplesius) wagae mustangensis* Deuve | NEPAL N-slope Annapurna Himal, south above Muktinath, alt. 4350 m | 28°48'N 83°52'E | hs0204 |
| *Carabus (Neoplesius) wagae nyalamensis* Deuve | CHINA South Tibet, N-slope Greater Himalaya, S of Nyalam, alt. 4000 m | 28°09'N 85°58'E | hs1214 |
| *Carabus (Neoplesius) wagae tanguticus* Semenov | CHINA Qinghai, Barganxiang, Gangbo N, alt. 4250 m | 33°58'N 96°34'E | hs1890 |
| *Carabus (Neoplesius) wagae wagae* Fairmaire | NEPAL N-slope Manaslu Himal, Dharmasala, alt. 4700 m | 28°40'N 84°35'E | hs0045 |
| *Carabus (Nesaeocarabus) abbreviatus* Brullé | SPAIN, Teneriffa, above Orotava, Los Organos, alt. 1200 m | 28°21'N 16°30'W | hs2737 |
| *Carabus (Ophiocarabus) latiballioni* Deuve | CHINA Xinjiang, ESE Tekes, Tagymbel, S Karatogai Vill., alt. 2200 m | 43°09'N 82°15'E | hs2273 |
| *Carabus (Ophiocarabus) praecox* Semenov | CHINA Xinjiang, Narat Mt. Range, Bodon Valley, alt. 3000-3240 m | 43°01'N 83°10'E | hs2274 |
| *Carabus (Ophiocarabus) tekesensis* Deuve & Tian | CHINA Xinjiang, Narat Mt. Range, NE of Tshon-Kushtai, alt. 2635 m | 42°56'N 82°15'E | hs2271 |
| *Carabus (Ophiocarabus) tekesensis* Deuve & Tian | CHINA Xinjiang, Narat Mt. Range, NE of Tshon-Kushtai, alt. 2500 m | 42°56'N 82°14'E | hs2272 |
| *Carabus (Orinocarabus) alpestris dolomitanus* Mandl | ITALY Dolomiti, Rosengarten, Tschagerjoch to Rotwandhütte, alt. 2500 m | 46°26'N 11°38'E | hs2850 |
| *Carabus (Orinocarabus) alpestris dolomitanus* Mandl | ITALY Dolomiti, Schlern, Schlernhaus to Rosenzahnscharte, alt. 2500 m | 46°30'N 11°37'E | hs2851 |
| *Carabus (Orinocarabus) baudii* Kraatz | ITALY Piemont, Crissolo, Monviso, Pian del Re, alt. 2600 m | 44°42'N 07°05'E | hs2646 |
| *Carabus (Orinocarabus) concolor* Fabricius | ITALY Valle d'Aosta, Valtournenche, Promiot to Mt. Zerebiom, alt. 2500 m | 45°47'N 07°36'E | hs2647 |
| *Carabus (Orinocarabus) heteromorphus* Daniel | ITALY Piemont, Gran Paradiso, Col de Nivolet, alt. 2600 m | 45°29'N 07°08'E | hs2643 |
| *Carabus (Orinocarabus) sylvestris* Panzer | GERMANY Saxonia, Schwarzwasser Valley, Kühnhaide to Pobershau, alt. 670 m | 50°37'N 13°14'E | hs1197 |
| *Carabus (Pachystus) glabratus* Paykull | RUSSIA Karelia, White Sea, Kindo Peninsula, alt. 10 m | 66°33'N 33°06'E | hs1058 |
| *Carabus (Pachystus) hortensis* L. | SWEDEN Smaland, near Väckelsang, alt. 150 m | 56°35'N 14°55'E | hs1059 |
| *Carabus (Pachystus) hortensis* L. | GERMANY Mecklenburg, Rostock, Rosenort, alt. 1 m | 54°14'N 12°11'E | hs1067 |
| *Carabus (Pachystus) preslii neumeyeri* Schaum | GREECE Kefallinia, Antisamos, alt. 100 m | 38°15'N 20°40'E | hs2652 |
| *Carabus (Pagocarabus) crassesculptus* Kraatz | CHINA Gansu, Station Tianshu, alt. 2900 m | 37°12'N 102°47'E | hs1210 |
| *Carabus (Pagocarabus) crassesculptus* Kraatz | CHINA Gansu, Aze Station, alt. 3550 m | 33°40'N 101°51'E | hs1893 |
| *Carabus (Pagocarabus) crassesculptus* Kraatz | CHINA Qinghai, road Qingshuihe-Yushu, alt. 4200 m | 33°12'N 97°26'E | hs2487 |
| *Carabus (Platycarabus) creutzeri* Fabricius | ITALY Dolomiti, Schlern, Schlernhaus to Rosenzahnscharte, alt. 2500 m | 46°30'N 11°37'E | hs2852 |
| *Carabus (Platycarabus) creutzeri* Fabricius | ITALY Dolomiti, Latemar, Poppekanzel, alt. 2300 m | 46°24'N 11°36'E | hs2853 |
| *Carabus (Platycarabus) creutzeri* Fabricius | ITALY Trentino, Passo Rolle S-slope, alt. 1700 m | 46°16'N 11°47'E | hs2982 |
| *Carabus (Platycarabus) creutzeri* Fabricius | ITALY Trentino, above Passo Rolle, alt. 2070 m | 46°17'N 11°48'E | hs2984 |
| *Carabus (Platycarabus) depressus* Bonelli | ITALY Aosta, Col Gr. San Bernardo, south slope, alt. 2450 m | 45°52'N 07°09'E | hs2642 |
| *Carabus (Platycarabus) irregularis* Fabricius | GERMANY Bayern, Garmisch-Patenkirchen, Alpspitze, alt. 1900 m | 47°26'N 11°03'E | hs2641 |
| *Carabus (Procrustes) coriaceus* L. | GERMANY Mecklenburg, Nienhagen, Ehbruch, alt. 10 m | 54°09'N 11°58'E | hs1099 |
| *Carabus (Procrustes) coriaceus* L. | GERMANY Mecklenburg, Admannshagen, alt. 8 m | 54°08'N 11°59'E | hs2849 |
| *Carabus (Pseudocranion) sackeni remondianus* Deuve | CHINA Sichuan, road E Maoxian-Songpan, 1 km SE Maanyao, alt. 3750 m | 32°00'N 103°45'E | hs2463 |
| *Carabus (Rhigocarabus) cateniger* Morawitz | CHINA Gansu, Pass NW Eboling, alt. 3700 m | 38°00'N 100°55'E | hs1212 |
| *Carabus (Rhigocarabus) ladygini* Semenov | CHINA Qinghai, 5 km SW Pass Bayankala, alt. 4680 m | 34°05'N 97°36'E | hs1211 |
| *Carabus (Rhigocarabus) ladygini* Semenov | CHINA Qinghai, Bayankala pass, alt. 4700 m | 34°08'N 97°39'E | hs2501 |
| *Carabus (Rhigocarabus) ladygini* Semenov | CHINA Qinghai, pass W Mt. Maqen Gangri, alt. 4750 m | 34°49'N 99°03'E | hs2507 |
| *Carabus (Rhigocarabus) morawitzianus mayumiae* Deuve & Imura | CHINA South Tibet, Nyainqentanglha Shan, Gyoze La, alt. 4900 m | 30°37' 91°31'E | hs0395 |
| *Carabus (Rhigocarabus) morawitzianus mayumiae* Deuve & Imura | CHINA South Tibet, Gangdise Shan E Reting, alt. 5100 m | 30°25'N 91°41'E | hs0404 |
| *Carabus (Rhigocarabus) roborowskii* Semenov | CHINA Qinghai, Barganxiang, Gangbo N, alt. 4250 m | 33°58'N 96°34'E | hs1894 |
| *Carabus (Rhigocarabus) roborowskii* Semenov | CHINA Qinghai, 80 km S Darleg, alt. 4400 m | 33°09'N 99°38'E | hs1895 |
| *Carabus (Sphodristocarabus) armeniacus* Mannerheim | ARMENIA Syunik, E-slope Vorotan-Pass, alt. 2330 m | 39° 41'N 45°43'E | hs2884 |
| *Carabus (Tachypus) auratus* L. | GERMANY Mecklenburg, Rostock, Kassebohm, alt. 5 m | 54°04'N 12°09'E | hs1062 |
| *Carabus (Tomocarabus) convexus convexus* Fabricius | GERMANY Sachsen-A., opencast mine Profen, alt. 175 m | 51°08'N 12°09'E | hs1066 |
| *Carabus (Tomocarabus) convexus pyrenaeicola* Csiki | FRANCE Hautes-Pyrénées, Col de Hautacam, alt. 1500 m | 42°58'N 00°00'E | hs2644 |
| *Carabus (Tomocarabus) convexus hornschuchi* Hoppe | GREECE Macedonia, Voras Oros, Kajmakcalon, alt. 1800 m | 40°54'N 21°50'E | hs2891 |
| *Carabus (Ulocarabus) theanus* Reitter | AFGHANISTAN Baglan, N-slope Salang Pass, alt. 2200 m | 35°27'N 68°59'E | hs0942 |
| *Cychrus (Cychropsis) mandli* (Paulus) | NEPAL Kali Gandaki Valley, Yakkharka above Marpha, alt. 3750 m | 28°45'N 83°39'E | hs0199 |
| *Cychrus (Cychrus) atennuatus* (Fabricius) | FRANCE Cévennes, dep. Ardèche, Croix de Bauzon, alt. 1200 m | 44°38'N 04°05'E | hs2656 |
| *Cychrus (Cychrus) caraboides* (L.) | GERMANY Mecklenburg, Rostock, Rosenort, alt. 1 m | 54°14'N 12°11'E | hs1093 |
| *Cychrus (Cychrus) kaznakovi tongrenicus* Deuve | CHINA Qinghai, 40 km N Maqen, alt. 3500 m | 34°44'N 100°14'E | hs2495 |
| *Cychrus (Cychrus) starcki* Reitter | ABKHAZIA southern Bertshil mountain range, alt. 1440 m | 43°24’N 40°16’E | hs2472 |
| *Cychrus (Cychrus) stoetzneri* Roeschke | CHINA, Sichuan, Amula hotspring, alt. 3980 m | 31°51'N 99°14'E | hs2657 |

Table S3. List of *Carabus* and outgroup species used in this study with information on gene fragment, voucher and GenBank accession number.

|  | COI | ND5 | ITS2 | 28S | 18S | CAD | wingless | PepCK | HUWE1 |
| --- | --- | --- | --- | --- | --- | --- | --- | --- | --- |
| *Calosoma (Calosoma) inquisitor* (L.) | OP756682 | OP761311 | OP757436 | OP758463 | OP758644 | OP761750 | n.a. | OP761608 | n.a. |
| *Calosoma (Campalita) maderae auropunctatum* (Herbst) | OP756651 | n.a. | OP757406 | OP758433 | OP758614 | OP761721 | OP761431 | OP761581 | OP745157 |
| *Calosoma (Carabomorphus) gestroi* Breuning | OP756652 | OP761281 | OP757407 | OP758434 | OP758615 | OP761722 | OP761432 | OP761582 | OP745158 |
| *Calosoma (Carabops) abyssinicum* Gestro | OP756689 | OP761316 | OP757441 | OP758470 | OP758651 | OP761757 | OP761467 | OP761613 | OP745188 |
| *Carabus (Acoptolabrus) gehinii* Fairmaire | LC553403 | D50429 | n.a. | LC567590 | n.a. | n.a. | AY183606 | AY183527 | n.a. |
| *Carabus (Apotomopterus) sauteri yunkaicus* Deuve | OP756604 | OP761235 | OP757361 | OP758383 | OP758564 | OP761672 | OP761383 | OP761535 | OP745113 |
| *Carabus (Apotomopterus) torquatus torquatus* Cavazzuti | OP756602 | n.a. | OP757359 | OP758381 | OP758562 | OP761670 | OP761381 | OP761533 | OP745111 |
| *Carabus (Archicarabus) nemoralis* Müller | OP756608 | OP761238 | OP757364 | OP758387 | OP758568 | OP761676 | OP761386 | OP761538 | OP745116 |
| *Carabus (Aulonocarabus) canaliculatus* *careniger* Chaudoir | OP756614 | OP761245 | OP757370 | OP758394 | OP758575 | OP761682 | OP761393 | OP761545 | OP745122 |
| *Carabus (Calocarabus) przewalskii elnarae* Cavazzuti | JQ646585 | n.a. | n.a. | JQ647375 | n.a. | n.a. | n.a. | n.a. | JQ646932 |
| *Carabus (Carabus) arvensis* Herbst | OP756596 | OP761227 | OP757354 | OP758374 | OP758555 | OP761663 | OP761374 | OP761526 | OP745106 |
| *Carabus (Carabus) granulatus* L. | n.a. | OP761230 | n.a. | OP758377 | OP758558 | OP761666 | OP761377 | OP761529 | n.a. |
| *Carabus (Carabus) granulatus* L. | OP756601 | OP761233 | n.a. | OP758380 | OP758561 | OP761669 | OP761380 | OP761532 | OP745110 |
| *Carabus (Carabus) menetriesi* Faldermann | OP756691 | OP761318 | OP757443 | OP758472 | OP758653 | OP761759 | OP761469 | OP761615 | OP745190 |
| *Carabus (Carpathophilus) linnaei* Panzer | OP756715 | OP761343 | OP757469 | OP758499 | OP758680 | OP761785 | OP761496 | OP761637 | OP745216 |
| *Carabus (Chaetocarabus) intricatus intricatus* L. | OP729898 | OP761240 | OP757366 | OP758389 | OP758570 | OP761678 | OP761388 | OP761540 | OP745118 |
| *Carabus (Chaetocarabus) intricatus krueperi* Reitter | OP756698 | OP761325 | OP757450 | OP758479 | OP758660 | OP761766 | OP761476 | OP761622 | OP745197 |
| *Carabus (Chrysocarabus) auronitens auronitens* Fabricius | OP756616 | OP761247 | OP757372 | OP758396 | OP758577 | OP761684 | OP761395 | OP761547 | OP745124 |
| *Carabus (Chrysocarabus) auronitens festivus* Dejean | OP756676 | OP761305 | OP757431 | OP758458 | OP758639 | OP761745 | OP761456 | OP761602 | OP745181 |
| *Carabus (Chrysocarabus) hispanus* Fabricius | OP756677 | OP761306 | OP757432 | OP758459 | OP758640 | OP761746 | OP761457 | OP761603 | OP745182 |
| *Carabus (Cratocephalus) solskyi* Ballion | OP756639 | OP761270 | OP757395 | OP758420 | OP758601 | OP761708 | OP761419 | OP761570 | OP745147 |
| *Carabus (Ctenocarabus) melancholicus* Fabricius | OP756641 | OP761273 | OP757398 | OP758423 | OP758604 | OP761711 | OP761422 | OP761573 | OP745149 |
| *Carabus (Cupreocarabus) huangi* Deuve | JQ646535 | n.a. | n.a. | JQ647330 | n.a. | n.a. | JQ646737 | JQ646837 | JQ646902 |
| *Carabus (Cytilocarabus) cribratus* Quensel | OP756701 | OP761328 | OP757453 | OP758482 | OP758663 | OP761769 | OP761479 | OP761625 | OP745200 |
| *Carabus (Damaster) elysii pulcher* Kleinfeld | OP756613 | OP761244 | OP757369 | OP758393 | OP758574 | OP761681 | OP761392 | OP761544 | OP745121 |
| *Carabus (Damaster)* *formosus* *grumorum* Semenov | OP756680 | OP761309 | OP757435 | OP758462 | OP758643 | OP761749 | OP761460 | OP761606 | OP745184 |
| *Carabus (Eurycarabus) famini* Dejean | JQ646543 | n.a. | n.a. | JQ647320 | n.a. | n.a. | n.a. | JQ646830 | JQ646896 |
| *Carabus (Eurycarabus) famini* Dejean | JQ646554 | n.a. | n.a. | JQ647337 | n.a. | n.a. | JQ646754 | JQ646844 | JQ646909 |
| *Carabus (Eurycarabus) famini* Dejean | JQ689878 | JQ689846 | JQ689669 | JQ689699 | n.a. | n.a. | n.a. | n.a. | JQ689756 |
| *Carabus (Eurycarabus) famini* Dejean | JQ689884 | JQ689853 | JQ689675 | JQ689705 | n.a. | n.a. | n.a. | n.a. | JQ689761 |
| *Carabus (Eurycarabus) genei* Gené | JQ646619 | AF231691 | n.a. | JQ647336 | n.a. | n.a. | n.a. | JQ646843 | JQ646908 |
| *Carabus (Hemicarabus) nitens* L. | OP756607 | OP761237 | OP757363 | OP758386 | OP758567 | OP761675 | OP761385 | OP761537 | OP745115 |
| *Carabus (Hypsocarabus) laotse* Breuning | JQ646616 | AB050750 | n.a. | JQ647409 | n.a. | n.a. | JQ646816 | n.a. | n.a. |
| *Carabus (Hypsocarabus) mikhaili loustei* Deuve & Mourzine | JQ646537 | n.a. | n.a. | JQ647315 | n.a. | n.a. | JQ646739 | n.a. | JQ646892 |
| *Carabus (Imaibius) cavifrons* Mandl | OP756726 | OP761354 | OP757479 | OP758510 | OP758691 | OP761796 | OP761506 | OP761646 | OP745226 |
| *Carabus (Imaibius) nouristani* Ledoux | OP756739 | OP761367 | OP757492 | OP758523 | OP758704 | OP761809 | OP761519 | OP761656 | OP745239 |
| *Carabus (Imaibius) olafi* Deuve & Schmidt | OP756743 | OP761371 | OP757496 | OP758527 | OP758708 | OP761813 | OP761523 | OP761660 | OP745243 |
| *Carabus (Isiocarabus) miaorum* Lassalle & Prunier | OP756603 | OP761234 | OP757360 | OP758382 | OP758563 | OP761671 | OP761382 | OP761534 | OP745112 |
| *Carabus (Lamprostus) calleyi* Fischer | OP756703 | OP761330 | OP757455 | OP758484 | OP758665 | OP761771 | OP761481 | OP761627 | OP745202 |
| *Carabus (Leptocarabus) seishinensis seunglaki* Kwon & Lee | JQ646580 | n.a. | n.a. | JQ647371 | n.a. | n.a. | JQ646783 | JQ646869 | n.a. |
| *Carabus (Leptocarabus) semiopacus* Reitter | JQ646578 | AB031456 | n.a. | JQ647369 | n.a. | n.a. | JQ646781 | AY183569 | JQ646928 |
| *Carabus (Leptocarabus) yokoae* Deuve | n.a. | AB031425 | n.a. | JQ647326 | n.a. | n.a. | n.a. | n.a. | n.a. |
| *Carabus (Limnocarabus) clatratus* L. | OP756597 | OP761228 | OP757355 | OP758375 | OP758556 | OP761664 | OP761375 | OP761527 | OP745107 |
| *Carabus (Macrothorax) morbillosus* Fabricius | JQ646591 | n.a. | n.a. | JQ647381 | n.a. | n.a. | JQ646791 | n.a. | JQ646936 |
| *Carabus (Macrothorax) morbillosus* Fabricius | JQ689883 | JQ689852 | JQ689674 | JQ689704 | n.a. | n.a. | n.a. | n.a. | JQ689760 |
| *Carabus (Macrothorax) morbillosus* Fabricius | JQ689896 | JQ689866 | JQ689687 | JQ689716 | n.a. | n.a. | n.a. | n.a. | JQ689772 |
| *Carabus (Macrothorax) morbillosus* Fabricius | JQ689897 | JQ689867 | JQ689688 | JQ689717 | n.a. | n.a. | n.a. | n.a. | JQ689773 |
| *Carabus (Macrothorax) morbillosus* Fabricius | JQ689898 | JQ689868 | JQ689689 | JQ689718 | n.a. | n.a. | n.a. | n.a. | JQ689774 |
| *Carabus (Macrothorax) morbillosus* Fabricius | JX279622 | JX279703 | JX279661 | JX279676 | n.a. | n.a. | n.a. | n.a. | JX279650 |
| *Carabus (Macrothorax) rugosus* Fabricius | JQ689882 | JQ689851 | JQ689673 | JQ689703 | n.a. | n.a. | n.a. | n.a. | JQ689759 |
| *Carabus (Macrothorax) rugosus* Fabricius | JQ689892 | JQ689861 | JQ689683 | JQ689712 | n.a. | n.a. | n.a. | n.a. | JQ689768 |
| *Carabus (Meganebrius) alanstivelli* Morvan | OP756685 | OP761312 | OP757437 | OP758466 | OP758647 | OP761753 | OP761463 | OP761609 | OP745185 |
| *Carabus (Meganebrius) arunensis* Heinertz | OP756653 | OP761282 | OP757408 | OP758435 | OP758616 | OP761723 | OP761433 | n.a. | OP745159 |
| *Carabus (Meganebrius) deliae deliae* Morvan | OP756628 | OP761259 | OP757384 | OP758408 | OP758589 | OP761696 | OP761407 | OP761559 | OP745136 |
| *Carabus (Meganebrius) deliae deliae* Morvan | OP756719 | OP761347 | OP757472 | OP758503 | OP758684 | OP761789 | OP761499 | OP761641 | OP745219 |
| *Carabus (Meganebrius) deliae deliae* Morvan | OP756731 | OP761359 | OP757484 | OP758515 | OP758696 | OP761801 | OP761511 | OP761651 | OP745231 |
| *Carabus (Meganebrius) deliae deliae* Morvan | OP756732 | OP761360 | OP757485 | OP758516 | OP758697 | OP761802 | OP761512 | OP761652 | OP745232 |
| *Carabus (Meganebrius) deliae deliae* Morvan | OP756735 | OP761363 | OP757488 | OP758519 | OP758700 | OP761805 | OP761515 | OP761655 | OP745235 |
| *Carabus (Meganebrius) deliae morvani* Lassalle | OP756627 | OP761258 | OP757383 | OP758407 | OP758588 | OP761695 | OP761406 | OP761558 | OP745135 |
| *Carabus (Meganebrius) deliae morvani* Lassalle | OP756706 | OP761334 | OP757459 | OP758488 | OP758669 | OP761775 | OP761485 | OP761631 | OP745206 |
| *Carabus (Meganebrius) epsteini annapurnae* Deuve & Schmidt | OP756729 | OP761357 | OP757482 | OP758513 | OP758694 | OP761799 | OP761509 | OP761649 | OP745229 |
| *Carabus (Meganebrius) epsteini epsteini* Heinertz | OP756626 | OP761257 | OP757382 | OP758406 | OP758587 | OP761694 | OP761405 | OP761557 | OP745134 |
| *Carabus (Meganebrius) epsteini epsteini* Heinertz | OP756633 | OP761264 | OP757389 | OP758413 | OP758594 | OP761701 | OP761412 | OP761564 | OP745141 |
| *Carabus (Meganebrius) epsteini yetiphilus* Deuve & Schmidt | OP756730 | OP761358 | OP757483 | OP758514 | OP758695 | OP761800 | OP761510 | OP761650 | OP745230 |
| *Carabus (Meganebrius) everesti everesti* Andrewes | OP756740 | OP761368 | OP757493 | OP758524 | OP758705 | OP761810 | OP761520 | OP761657 | OP745240 |
| *Carabus (Meganebrius) everesti everesti* Andrewes | OP756708 | OP761336 | OP757461 | OP758490 | OP758671 | OP761777 | OP761487 | OP761633 | OP745208 |
| *Carabus (Meganebrius) everesti everesti* Andrewes | OP756712 | OP761340 | OP757465 | OP758494 | OP758675 | OP761781 | OP761491 | OP761636 | OP745212 |
| *Carabus (Meganebrius) everesti kleinfeldi* Korell | OP756659 | OP761288 | OP757414 | OP758441 | OP758622 | n.a. | OP761439 | OP761587 | OP745165 |
| *Carabus (Meganebrius) franzi* Mandl | OP756658 | OP761287 | OP757413 | OP758440 | OP758621 | OP761728 | OP761438 | OP761586 | OP745164 |
| *Carabus (Meganebrius) granulatocostatus* Mandl | OP756665 | OP761294 | OP757420 | OP758447 | OP758628 | OP761734 | OP761445 | OP761593 | OP745171 |
| *Carabus (Meganebrius) indicus* Fairmaire | OP756655 | OP761284 | OP757410 | OP758437 | OP758618 | OP761725 | OP761435 | n.a. | OP745161 |
| *Carabus (Meganebrius) kadoudali* Morvan | OP756660 | OP761289 | OP757415 | OP758442 | OP758623 | OP761729 | OP761440 | OP761588 | OP745166 |
| *Carabus (Meganebrius) kadoudali* Morvan | OP756707 | OP761335 | OP757460 | OP758489 | OP758670 | OP761776 | OP761486 | OP761632 | OP745207 |
| *Carabus (Meganebrius) koganae* Colas | OP756662 | OP761291 | OP757417 | OP758444 | OP758625 | OP761731 | OP761442 | OP761590 | OP745168 |
| *Carabus (Meganebrius) lebretae densestriolatus* Mandl | OP756687 | OP761314 | OP757439 | OP758468 | OP758649 | OP761755 | OP761465 | OP761611 | OP745187 |
| *Carabus (Meganebrius) lebretae lebretae* Colas | OP756663 | OP761292 | OP757418 | OP758445 | OP758626 | OP761732 | OP761443 | OP761591 | OP745169 |
| *Carabus (Meganebrius) lebretae lebretae* Colas | OP756664 | OP761293 | OP757419 | OP758446 | OP758627 | OP761733 | OP761444 | OP761592 | OP745170 |
| *Carabus (Meganebrius) lebretae lebretae* Colas | OP756711 | OP761339 | OP757464 | OP758493 | OP758674 | OP761780 | OP761490 | OP761635 | OP745211 |
| *Carabus (Meganebrius) montreuili* Deuve | OP756654 | OP761283 | OP757409 | OP758436 | OP758617 | OP761724 | OP761434 | OP761583 | OP745160 |
| *Carabus (Meganebrius) montreuili* Deuve | OP756661 | OP761290 | OP757416 | OP758443 | OP758624 | OP761730 | OP761441 | OP761589 | OP745167 |
| *Carabus (Meganebrius) montreuili* Deuve | OP756686 | OP761313 | OP757438 | OP758467 | OP758648 | OP761754 | OP761464 | OP761610 | OP745186 |
| *Carabus (Meganebrius) pseudoharmandi* Mandl | OP756667 | OP761296 | OP757422 | OP758449 | OP758630 | OP761736 | OP761447 | n.a. | n.a. |
| *Carabus (Meganebrius) queinneci* Deuve | OP756650 | OP761280 | OP757405 | OP758432 | OP758613 | OP761720 | OP761430 | OP761580 | OP745156 |
| *Carabus (Meganebrius) quinlani brittoni* Mandl | OP756640 | OP761271 | OP757396 | OP758421 | OP758602 | OP761709 | OP761420 | OP761571 | OP745148 |
| *Carabus (Meganebrius) quinlani brittoni* Mandl | OP756720 | OP761348 | OP757473 | OP758504 | OP758685 | OP761790 | OP761500 | OP761642 | OP745220 |
| *Carabus (Meganebrius) quinlani brittoni* Mandl | OP756721 | OP761349 | OP757474 | OP758505 | OP758686 | OP761791 | OP761501 | OP761643 | OP745221 |
| *Carabus (Meganebrius) quinlani quinlani* Mandl | OP756625 | OP761256 | OP757381 | OP758405 | OP758586 | OP761693 | OP761404 | OP761556 | OP745133 |
| *Carabus (Meganebrius) quinlani quinlani* Mandl | OP756736 | OP761364 | OP757489 | OP758520 | OP758701 | OP761806 | OP761516 | n.a. | OP745236 |
| *Carabus (Meganebrius) quinlani quinlani* Mandl | OP756737 | OP761365 | OP757490 | OP758521 | OP758702 | OP761807 | OP761517 | n.a. | OP745237 |
| *Carabus (Meganebrius) quinlani quinlani* Mandl | OP756693 | OP761320 | OP757445 | OP758474 | OP758655 | OP761761 | OP761471 | OP761617 | OP745192 |
| *Carabus (Meganebrius) quinlani quinlani* Mandl | OP756700 | OP761327 | OP757452 | OP758481 | OP758662 | OP761768 | OP761478 | OP761624 | OP745199 |
| *Carabus (Meganebrius) quinlani quinlani* Mandl | OP756733 | OP761361 | OP757486 | OP758517 | OP758698 | OP761803 | OP761513 | OP761653 | OP745233 |
| *Carabus (Meganebrius) quinlani quinlani* Mandl | OP756734 | OP761362 | OP757487 | OP758518 | OP758699 | OP761804 | OP761514 | OP761654 | OP745234 |
| *Carabus (Meganebrius) quinlani smetanai* Häckel & Brezina | OP756618 | OP761249 | OP757374 | OP758398 | OP758579 | OP761686 | OP761397 | OP761549 | OP745126 |
| *Carabus (Meganebrius) quinlani smetanai* Häckel & Brezina | n.a. | OP761333 | OP757458 | OP758487 | OP758668 | OP761774 | OP761484 | OP761630 | OP745205 |
| *Carabus (Meganebrius) salpansis* Deuve | OP756738 | OP761366 | OP757491 | OP758522 | OP758703 | OP761808 | OP761518 | n.a. | OP745238 |
| *Carabus (Meganebrius) salpansis* Deuve | OP756666 | OP761295 | OP757421 | OP758448 | OP758629 | OP761735 | OP761446 | n.a. | OP745172 |
| *Carabus (Meganebrius) santostamangi santostamangi* Deuve & Schmidt | n.a. | n.a. | OP757466 | OP758495 | OP758676 | OP761782 | OP761492 | n.a. | OP745213 |
| *Carabus (Meganebrius) santostamangi santostamangi* Deuve & Schmidt | OP729899 | OP761341 | OP757467 | OP758496 | OP758677 | OP761783 | OP761493 | n.a. | OP745214 |
| *Carabus (Meganebrius) santostamangi thanglaensis* Deuve & Schmidt | OP756714 | n.a. | n.a. | OP758498 | OP758679 | n.a. | OP761495 | n.a. | n.a. |
| *Carabus (Meganebrius) scheibei* Eidam | OP756681 | OP761310 | n.a. | n.a. | n.a. | n.a. | OP761461 | OP761607 | n.a. |
| *Carabus (Meganebrius)* sp.n. near *granulatocostatus* | OP756741 | OP761369 | OP757494 | OP758525 | OP758706 | OP761811 | OP761521 | OP761658 | OP745241 |
| *Carabus (Meganebrius) tamang gurkhaorum* Lassalle | n.a. | OP761272 | OP757397 | OP758422 | OP758603 | OP761710 | OP761421 | OP761572 | n.a. |
| *Carabus (Meganebrius) tamang probsti* Deuve | OP756727 | OP761355 | OP757480 | OP758511 | OP758692 | OP761797 | OP761507 | OP761647 | OP745227 |
| *Carabus (Meganebrius) tamang tamang* Deuve | OP756643 | OP761275 | OP757400 | OP758425 | OP758606 | OP761713 | OP761424 | OP761575 | OP745151 |
| *Carabus (Meganebrius) thudamensis* Deuve & Schmidt | OP756713 | OP761342 | OP757468 | OP758497 | OP758678 | OP761784 | OP761494 | n.a. | OP745215 |
| *Carabus (Meganebrius) tuberculipennis ganjalaensis* Deuve | OP756710 | OP761338 | OP757463 | OP758492 | OP758673 | OP761779 | OP761489 | OP761634 | OP745210 |
| *Carabus (Meganebrius) tuberculipennis* ssp.n. | OP756656 | OP761285 | OP757411 | OP758438 | OP758619 | OP761726 | OP761436 | OP761584 | OP745162 |
| *Carabus (Meganebrius) tuberculipennis tuberculipennis* Mandl | OP756705 | OP761332 | OP757457 | OP758486 | OP758667 | OP761773 | OP761483 | OP761629 | OP745204 |
| *Carabus (Meganebrius) wallichi* Hope | OP756657 | OP761286 | OP757412 | OP758439 | OP758620 | OP761727 | OP761437 | OP761585 | OP745163 |
| *Carabus (Megodontus) germarii* Sturm | OP756717 | OP761345 | OP757471 | OP758501 | OP758682 | OP761787 | OP761497 | OP761639 | OP745217 |
| *Carabus (Megodontus) violaceus dryas* Gistel | OP756699 | OP761326 | OP757451 | OP758480 | OP758661 | OP761767 | OP761477 | OP761623 | OP745198 |
| *Carabus (Megodontus) violaceus purpurascens* Fabricius | OP756678 | OP761307 | OP757433 | OP758460 | OP758641 | OP761747 | OP761458 | OP761604 | OP745183 |
| *Carabus (Megodontus) violaceus purpurascens* Fabricius | OP756690 | OP761317 | OP757442 | OP758471 | OP758652 | OP761758 | OP761468 | OP761614 | OP745189 |
| *Carabus (Megodontus) violaceus violaceus* L. | OP756605 | OP761236 | OP757362 | OP758384 | OP758565 | OP761673 | OP761384 | OP761536 | OP745114 |
| *Carabus (Mesocarabus) dufourii* Dejean | JQ689936 | JQ689873 | JQ689694 | JQ689722 | n.a. | n.a. | n.a. | n.a. | JQ689778 |
| *Carabus (Mesocarabus) dufourii* Dejean | JX278213 | n.a. | JX278889 | JX279131 | n.a. | n.a. | n.a. | n.a. | JX278660 |
| *Carabus (Mesocarabus) lusitanicus lusitanicus* Fabricius | OP756642 | OP761274 | OP757399 | OP758424 | OP758605 | OP761712 | OP761423 | OP761574 | OP745150 |
| *Carabus (Mesocarabus) macrocephalus* Dejean | JQ689879 | JQ689847 | JQ689670 | JQ689700 | n.a. | n.a. | n.a. | n.a. | JQ689757 |
| *Carabus (Mesocarabus) problematicus andorranus* Lapouge | OP756672 | OP761301 | OP757427 | OP758454 | OP758635 | OP761741 | OP761452 | OP761598 | OP745177 |
| *Carabus (Mesocarabus) problematicus problematicus* Herbst | OP756615 | OP761246 | OP757371 | OP758395 | OP758576 | OP761683 | OP761394 | OP761546 | OP745123 |
| *Carabus (Mesocarabus) problematicus problematicus* Herbst | OP756675 | OP761304 | OP757430 | OP758457 | OP758638 | OP761744 | OP761455 | OP761601 | OP745180 |
| *Carabus (Mesocarabus) riffensis* Fairmaire | JQ689881 | JQ689850 | JQ689672 | JQ689702 | n.a. | n.a. | n.a. | n.a. | JQ689758 |
| *Carabus (Mesocarabus) riffensis* Fairmaire | JX278137 | JX279488 | JX278812 | JX279055 | HG813129 | n.a. | n.a. | n.a. | JX278600 |
| *Carabus (Mimocarabus) maurus* (Adams) | OP756702 | OP761329 | OP757454 | OP758483 | OP758664 | OP761770 | OP761480 | OP761626 | OP745201 |
| *Carabus (Neoplesius) borodini* Heinz | OP756624 | OP761255 | OP757380 | OP758404 | OP758585 | OP761692 | OP761403 | OP761555 | OP745132 |
| *Carabus (Neoplesius) danae* Kaláb | OP756611 | OP761242 | OP757368 | OP758391 | OP758572 | OP761680 | OP761390 | OP761542 | OP745120 |
| *Carabus (Neoplesius) nanschanicus nocticolor* Deuve & Kaláb | JQ646540 | n.a. | n.a. | JQ647318 | n.a. | n.a. | JQ646741 | JQ646828 | JQ646894 |
| *Carabus (Neoplesius) paulusi kocmani* Kaláb | OP756623 | OP761254 | OP757379 | OP758403 | OP758584 | OP761691 | OP761402 | OP761554 | OP745131 |
| *Carabus (Neoplesius) paulusi paulusi* Kaláb | OP756724 | OP761352 | OP757477 | OP758508 | OP758689 | OP761794 | OP761504 | OP761645 | OP745224 |
| *Carabus (Neoplesius) wagae folwarzcnyi* Deuve | OP756722 | OP761350 | OP757475 | OP758506 | OP758687 | OP761792 | OP761502 | OP761644 | OP745222 |
| *Carabus (Neoplesius) wagae glasunowi* Heinz | OP756612 | OP761243 | n.a. | OP758392 | OP758573 | n.a. | OP761391 | OP761543 | n.a. |
| *Carabus (Neoplesius) wagae glinkai* Heinz | OP756610 | OP761241 | OP757367 | OP758390 | OP758571 | OP761679 | OP761389 | OP761541 | OP745119 |
| *Carabus (Neoplesius) wagae mustangensis* Deuve | OP756635 | OP761265 | OP757390 | OP758415 | OP758596 | OP761703 | OP761414 | OP761565 | OP745142 |
| *Carabus (Neoplesius) wagae nyalamensis* Deuve | OP756622 | OP761253 | OP757378 | OP758402 | OP758583 | OP761690 | OP761401 | OP761553 | OP745130 |
| *Carabus (Neoplesius) wagae tanguticus* Semenov | OP756629 | OP761260 | OP757385 | OP758409 | OP758590 | OP761697 | OP761408 | OP761560 | OP745137 |
| *Carabus (Neoplesius) wagae wagae* Fairmaire | OP756728 | OP761356 | OP757481 | OP758512 | OP758693 | OP761798 | OP761508 | OP761648 | OP745228 |
| *Carabus (Nesaeocarabus) abbreviatus* Brullé | OP756688 | OP761315 | OP757440 | OP758469 | OP758650 | OP761756 | OP761466 | OP761612 | n.a. |
| *Carabus (Ophiocarabus) latiballioni* Deuve | OP756637 | OP761268 | OP757393 | OP758418 | OP758599 | OP761706 | OP761417 | OP761568 | OP745145 |
| *Carabus (Ophiocarabus) praecox* Semenov | OP756638 | OP761269 | OP757394 | OP758419 | OP758600 | OP761707 | OP761418 | OP761569 | OP745146 |
| *Carabus (Ophiocarabus) tekesensis* Deuve & Tian | n.a. | OP761266 | OP757391 | OP758416 | OP758597 | OP761704 | OP761415 | OP761566 | OP745143 |
| *Carabus (Ophiocarabus) tekesensis* Deuve & Tian | OP756636 | OP761267 | OP757392 | OP758417 | OP758598 | OP761705 | OP761416 | OP761567 | OP745144 |
| *Carabus (Orinocarabus) alpestris dolomitanus* Mandl | OP756694 | OP761321 | OP757446 | OP758475 | OP758656 | OP761762 | OP761472 | OP761618 | OP745193 |
| *Carabus (Orinocarabus) alpestris dolomitanus* Mandl | OP756695 | OP761322 | OP757447 | OP758476 | OP758657 | OP761763 | OP761473 | OP761619 | OP745194 |
| *Carabus (Orinocarabus) baudii* Kraatz | OP756673 | OP761302 | OP757428 | OP758455 | OP758636 | OP761742 | OP761453 | OP761599 | OP745178 |
| *Carabus (Orinocarabus) concolor* Fabricius | OP756674 | OP761303 | OP757429 | OP758456 | OP758637 | OP761743 | OP761454 | OP761600 | OP745179 |
| *Carabus (Orinocarabus) heteromorphus* Daniel | OP756670 | OP761299 | OP757425 | OP758452 | OP758633 | OP761739 | OP761450 | OP761596 | OP745175 |
| *Carabus (Orinocarabus) sylvestris* Panzer | OP756617 | OP761248 | OP757373 | OP758397 | OP758578 | OP761685 | OP761396 | OP761548 | OP745125 |
| *Carabus (Pachystus) glabratus* Paykull | OP756594 | OP761225 | OP757352 | OP758372 | OP758553 | OP761661 | OP761372 | OP761524 | OP745105 |
| *Carabus (Pachystus) hortensis* L. | OP756595 | OP761226 | OP757353 | OP758373 | OP758554 | OP761662 | OP761373 | OP761525 | n.a. |
| *Carabus (Pachystus) hortensis* L. | OP756600 | OP761232 | OP757358 | OP758379 | OP758560 | OP761668 | OP761379 | OP761531 | n.a. |
| *Carabus (Pachystus) preslii neumeyeri* Schaum | OP756679 | OP761308 | OP757434 | OP758461 | OP758642 | OP761748 | OP761459 | OP761605 | n.a. |
| *Carabus (Pagocarabus) crassesculptus* Kraatz | OP756619 | OP761250 | OP757375 | OP758399 | OP758580 | OP761687 | OP761398 | OP761550 | OP745127 |
| *Carabus (Pagocarabus) crassesculptus* Kraatz | OP756630 | OP761261 | OP757386 | OP758410 | OP758591 | OP761698 | OP761409 | OP761561 | OP745138 |
| *Carabus (Pagocarabus) crassesculptus* Kraatz | OP756646 | OP761277 | OP757402 | OP758428 | OP758609 | OP761716 | OP761426 | OP761577 | OP745153 |
| *Carabus (Piocarabus) reitterianus* Breuning | JQ646533 | AB092733 | n.a. | JQ647311 | n.a. | n.a. | JQ646735 | n.a. | n.a. |
| *Carabus (Piocarabus) titanus* Breuning | JQ646532 | AB092726 | n.a. | JQ647310 | n.a. | n.a. | JQ646734 | JQ646823 | n.a. |
| *Carabus (Platycarabus) creutzeri* Fabricius | OP756696 | OP761323 | OP757448 | OP758477 | OP758658 | OP761764 | OP761474 | OP761620 | OP745195 |
| *Carabus (Platycarabus) creutzeri* Fabricius | OP756697 | OP761324 | OP757449 | OP758478 | OP758659 | OP761765 | OP761475 | OP761621 | OP745196 |
| *Carabus (Platycarabus) creutzeri* Fabricius | OP756716 | OP761344 | OP757470 | OP758500 | OP758681 | OP761786 | n.a. | OP761638 | n.a. |
| *Carabus (Platycarabus) creutzeri* Fabricius | OP756718 | OP761346 | n.a. | OP758502 | OP758683 | OP761788 | OP761498 | OP761640 | OP745218 |
| *Carabus (Platycarabus) depressus* Bonelli | OP756669 | OP761298 | OP757424 | OP758451 | OP758632 | OP761738 | OP761449 | OP761595 | OP745174 |
| *Carabus (Platycarabus) irregularis* Fabricius | OP756668 | OP761297 | OP757423 | OP758450 | OP758631 | OP761737 | OP761448 | OP761594 | OP745173 |
| *Carabus (Procrustes) coriaceus* L. | OP756609 | OP761239 | OP757365 | OP758388 | OP758569 | OP761677 | OP761387 | OP761539 | OP745117 |
| *Carabus (Procrustes) coriaceus* L. | OP756692 | OP761319 | OP757444 | OP758473 | OP758654 | OP761760 | OP761470 | OP761616 | OP745191 |
| *Carabus (Pseudocranion) gansuensis* Semenov | JQ646587 | AB101183 | n.a. | JQ647377 | HG813126 | n.a. | AY183614 | AY183535 | JQ646933 |
| *Carabus (Pseudocranion) sackeni remondianus* Deuve | OP756644 | OP761276 | OP757401 | OP758426 | OP758607 | OP761714 | n.a. | OP761576 | OP745152 |
| *Carabus (Pseudocranion) tibetanophilus yak* Deuve | JQ646534 | n.a. | n.a. | JQ647312 | n.a. | n.a. | JQ646736 | JQ646824 | JQ646890 |
| *Carabus (Rhigocarabus) buddaicus gansuicus* Deuve | JQ646550 | AB092755 | n.a. | JQ647329 | n.a. | n.a. | JQ646749 | JQ646836 | n.a. |
| *Carabus (Rhigocarabus) cateniger* Morawitz | OP756621 | OP761252 | OP757377 | OP758401 | OP758582 | OP761689 | OP761400 | OP761552 | OP745129 |
| *Carabus (Rhigocarabus) gracilicollis shatanensis* Deuve & Tian | JQ646536 | n.a. | n.a. | JQ647314 | n.a. | n.a. | JQ646738 | JQ646825 | n.a. |
| *Carabus (Rhigocarabus) ladygini* Semenov | OP756620 | OP761251 | OP757376 | OP758400 | OP758581 | OP761688 | OP761399 | OP761551 | OP745128 |
| *Carabus (Rhigocarabus) ladygini* Semenov | OP756648 | OP761278 | OP757403 | OP758430 | OP758611 | OP761718 | OP761428 | OP761578 | OP745154 |
| *Carabus (Rhigocarabus) ladygini* Semenov | OP756649 | OP761279 | OP757404 | OP758431 | OP758612 | OP761719 | OP761429 | OP761579 | OP745155 |
| *Carabus (Rhigocarabus) morawitzianus* Semenov | OP756723 | OP761351 | OP757476 | OP758507 | OP758688 | OP761793 | OP761503 | n.a. | OP745223 |
| *Carabus (Rhigocarabus) morawitzianus* Semenov | OP756725 | OP761353 | OP757478 | OP758509 | OP758690 | OP761795 | OP761505 | n.a. | OP745225 |
| *Carabus (Rhigocarabus) roborowskii* Semenov | OP756631 | OP761262 | OP757387 | OP758411 | OP758592 | OP761699 | OP761410 | OP761562 | OP745139 |
| *Carabus (Rhigocarabus) roborowskii* Semenov | OP756632 | OP761263 | OP757388 | OP758412 | OP758593 | OP761700 | OP761411 | OP761563 | OP745140 |
| *Carabus (Rhigocarabus) xiei abaxianensis* Deuve & Tian | JQ646539 | n.a. | n.a. | JQ647317 | n.a. | n.a. | JQ646740 | JQ646827 | n.a. |
| *Carabus (Sphodristocarabus) armeniacus* Mannerheim | OP756704 | OP761331 | OP757456 | OP758485 | OP758666 | OP761772 | OP761482 | OP761628 | OP745203 |
| *Carabus (Tachypus) auratus* L. | OP756598 | OP761229 | OP757356 | OP758376 | OP758557 | OP761665 | OP761376 | OP761528 | OP745108 |
| *Carabus (Tomocarabus) convexus convexus* Fabricius | OP756599 | OP761231 | OP757357 | OP758378 | OP758559 | OP761667 | OP761378 | OP761530 | OP745109 |
| *Carabus (Tomocarabus) convexus convexus* Fabricius | OP756671 | OP761300 | OP757426 | OP758453 | OP758634 | OP761740 | OP761451 | OP761597 | OP745176 |
| *Carabus (Tomocarabus) convexus hornschuchi* Hoppe | OP756709 | OP761337 | OP757462 | OP758491 | OP758672 | OP761778 | OP761488 | n.a. | OP745209 |
| *Carabus (Tomocarabus) fraterculus jirisanensis* Ishikawa & Kim | JQ646577 | n.a. | n.a. | JQ647368 | n.a. | n.a. | JQ646780 | n.a. | n.a. |
| *Carabus (Tomocarabus) rumelicus syriensis* Breuning | n.a. | n.a. | n.a. | JQ647339 | n.a. | n.a. | JQ646756 | JQ646846 | n.a. |
| *Carabus (Ulocarabus) theanus* Reitter | OP756742 | OP761370 | OP757495 | OP758526 | OP758707 | OP761812 | OP761522 | OP761659 | OP745242 |
| *Cychrus (Cychropsis) mandli* (Paulus) | OP756634 | n.a. | n.a. | OP758414 | OP758595 | OP761702 | OP761413 | n.a. | n.a. |
| *Cychrus (Cychrus) atennuatus* (Fabricius) | OP756683 | n.a. | n.a. | OP758464 | OP758645 | OP761751 | n.a. | n.a. | n.a. |
| *Cychrus (Cychrus) caraboides* (L.) | OP756606 | n.a. | n.a. | OP758385 | OP758566 | OP761674 | n.a. | n.a. | n.a. |
| *Cychrus (Cychrus) kaznakovi tongrenicus* Deuve | OP756647 | n.a. | n.a. | OP758429 | OP758610 | OP761717 | OP761427 | n.a. | n.a. |
| *Cychrus (Cychrus) starcki* Reitter | OP756645 | n.a. | n.a. | OP758427 | OP758608 | OP761715 | OP761425 | n.a. | n.a. |
| *Cychrus (Cychrus) stoetzneri* Roeschke | OP756684 | n.a. | n.a. | OP758465 | OP758646 | OP761752 | OP761462 | n.a. | n.a. |

Table S4. List of primers used in this study.

| Gene | Primer | Direction | Sequence (5’-3’) | Reference |
| --- | --- | --- | --- | --- |
| COI | LCO1490 | forward | GGT CAA CAA ATC ATA AAG ATA TTG G | [1] |
|  | wagCOIfw | forward | CTA GTA TTG CCC ATA GAG GGG CAT C | this study |
|  | JER | forward | CAA CAT TTA TTT TGA TTT TTT GG | [2] |
|  | COI 981F | forward | GGA TTA ACA GGA GTA GTA TTA GCT | this study |
|  | QuinRev | reverse | TGT TGT AAT AAA RTT TAC AGC YCC | this study |
|  | CarabusMT | reverse | YCC YAA AGA ACC AAA GGT TTC CT | this study |
|  | COI 1085R | reverse | TAC TGC TCC TAT TGA TAA AAC A | this study |
|  | PATnew | reverse | TCT AAT ATG GCA GAW TAG TGC AHT | [3] |
| ND5 | V1.61 | forward | CCT GTT TCT GCT TTA GTT CA | [4] |
|  | V1.04 | reverse | GTC ATA CTC TAA ATA TAA GCT A | [4] |
|  | ND5-569R | reverse | CCC TCA TCA TTT AAA GAA TG | this study |
|  | ND5-421F | forward | GTA GTT TCT ATA GAT TTT GT | this study |
| 28S | D1 | forward | GGG AGG AAA AGA AAC TAA C | [5] |
|  | D3i | reverse | GCA TAG TTC ACC ATC TTT C | [6] |
|  | 28S-655R | reverse | CAA CAC CAC AAG TCA TAC | this study |
|  | 28S-477F | forward | GTT GAG TTG TTG TTC AGT | this study |
| 18S | 18Sfw | forward | CCT AYC TGG TTG ATC CTG CCA GT | [7] |
|  | 18Srev | reverse | TAA TGA TCC TTC CGC AGG TT | [7] |
|  | 18S-397F | forward | TAC CAC ATC CAA GGA AGG CAG | this study |
|  | 18S-977F | forward | TTC GAA GGC GAT CAG ATA CCG | this study |
|  | 18S-1548F | forward | GGC GTA CAA TTG AAT CTT CTT | this study |
|  | 18S-577R | reverse | TGG AAT TAC CGC GGC TGC TG | this study |
|  | 18S-1220R | reverse | GCC CTT CCG TCA ATT CCT TT | this study |
|  | 18S-1678R | reverse | GAG GAC ACG CTG ATT CCT TCA | this study |
| ITS2 | ITS3 | forward | GCA TCG ATG AAG AAC GCA GC | [8] |
|  | ITS28 | reverse | CGC CGT TAC TAG GGG AAT CCT TGT AAG | [9] |
| CAD | CD806F | forward | GTN GTN AAR ATG CCN MGN TGG GA | [10] |
|  | CD1231R | reverse | TCC ACG TGT TCN GAN ACN GCC ATR CA | [11] |
|  | CD1098R | reverse | GCT ATG TTG TTN GGN AGY TGD CCN CCC AT | [10] |
| wingless | CARWL1 | forward | ATG TCT GGC ACC TGC ACC GT | [12] |
|  | CARWL2 | reverse | CAA GCG CAC CGT TCC ACA ACG A | [12] |
| PEPCK | CARPEK1 | forward | GCC ATG ATG ACA CCA ACA CT | [13] |
|  | CARPEK3 | reverse | GAC GTG GAA GAT CTT GGG CA | [13] |
| HUWE1 | gwenck1 | forward | GTG ACG AAC AAG AAG ATA TGG | [14] |
|  | carck2 | reverse | GTG GTT CGC ATC TCA ACA GA | [13] |

[1] O. Folmer, et al., DNA primers for amplification of mitochondrial cytochrome c oxidase subunit I from diverse metazoan invertebrates. *Mol. Marine Biol. Biotechnol.* 3, 294–299 (1994).

[2] C. Simon, et al., Evolution, weighting, and phylogenetic utility of mitochondrial gene sequences and a compilation of conserved polymerase chain reaction primers. *Ann. Entomol. Soc. Am.* 87, 651–701 (1994).

[3] J. Schmidt, et al., Into the Himalayan exile: the phylogeography of the ground beetle *Ethira* clade supports the Tibetan origin of forest-dwelling Himalayan species groups. *PLoS One* 7, e45482 (2012).

[4] S. Osawa, Z.H. Su, Y. Imura, Molecular Phylogeny and Evolution of Carabid Ground Beetles, (Springer, 2004), pp. 205.

[5] K. Ober, Phylogenetic relationships of the carabid subfamily Harpalinae (Coleoptera) based on molecular sequence data. *Mol. Phylogenet. Evol*. 24, 228–248 (2002).

[6] K. W. Will, A. S. Gill, Phylogeny and classification of *Hypherpes auctorum* (Coleoptera: Carabidae: Pterostichini: *Pterostichus*). *Ann. Carnegie Mus*. 77, 93–127 (2008).

[7] U. Englisch, S. Koenemann, Preliminary phytogenetic analysis of selected subterranean amphipod crustaceans, using small subunit rDNA gene sequences. *Org. Divers. Evol.* 1, 139–145 (2001).

[8] T. White, et al., “Amplification and direct sequencing of fungal ribosomal RNA genes for phylogenetics” in PCR Protocols: A Guide to Methods and Applications, M. A. Innis, et al., Eds. (Academic Press, 1990), pp. 315–322.

[9] S. J. Wagstaff, P.J. Garnock-Jones, Evolution and biogeography of the Hebe complex (Scrophulariaceae) inferred from ITS sequences. *N.Z. J. Bot.* 36, 425–437 (1998).

[10] J. K. Moulton, B. M. Wiegmann, Evolution and phylogenetic utility of CAD (rudimentary) among Mesozoic-aged Eremoneuran Diptera (Insecta). *Mol. Phylogenet. Evol*. 31, 363–378 (2004).

[11] A. L. Wild, D. R. Maddison, Evaluating nuclear protein-coding genes for phylogenetic utility in beetles. *Mol. Phylogenet. Evol*. 48, 877–891 (2008).

[12] A. B. Zhang, et al., Species status and phylogeography of two closely related *Coptolabrus* species (Coleoptera: Carabidae) in South Korea inferred from mitochondrial and nuclear gene sequences. *Mol. Ecol.* 14, 3823–3841 (2005).

[13] T. Sota, A. P. Vogler, Incongruence of mitochondrial and nuclear gene trees in the carabid beetles *Ohomopterus*. *Syst*. *Biol.* 50, 39–59 (2001).

[14] T. Deuve, et al., Molecular systematics and evolutionary history of the genus *Carabus* (Col. Carabidae). *Mol. Phylogenet. Evol*. 65, 259–75 (2012).

Table S5. PCR conditions applied in this study.

| Primer fw | Primer rev | PCR conditions | No. of cycles |
| --- | --- | --- | --- |
| LCO1490 | PATnew | 94°C/5 min; 94°C/1 min; 52.5°C/1 min; 72°C/1.5 min; 72°C/10 min | 38 |
| LCO1490 | CarabusMT | 94°C/5 min; 94°C/1 min; 52.5°C/1 min; 72°C/1.5 min; 72°C/10 min | 38 |
| JER | PATnew | 94°C/5 min; 94°C/1 min; 53°C/1 min; 72°C/1 min; 72°C/10 min | 38 |
| LCO1490 | QuinRev | 94°C/5 min; 94°C/1 min; 46°C/1 min; 72°C/1.5 min; 72°C/10 min | 38 |
| wagCOIfw | CarabusMT | 94°C/5 min; 94°C/1 min; 54°C/1 min; 72°C/1.5 min; 72°C/10 min | 38 |
| JER | COI 1085R | 94°C/5 min; 94°C/0.5 min; 47.5°C/0.5 min; 72°C/2 min; 72°C/7 min | 36 |
| COI 981F | PATnew | 94°C/5 min; 94°C/0.5 min; 49°C/ 0.5 min; 72°C/2 min; 72°C/7 min | 36 |
| V1.61 | V1.04 | 94°C/5 min; 94°C/1 min; 50°C/2 min; 72°C/2 min; 72°C/7 min | 35 |
| V1.61 | ND5-569R | 94°C/5 min; 94°C/0.5 min; 45°C/0.5 min; 72°C/2 min; 72°C/7 min | 35 |
| ND5-421F | V1.04 | 94°C/5 min; 94°C/0.5 min; 42°C/0.5 min; 72°C/2 min; 72°C/7 min | 35 |
| D1 | D3i | 94°C/5 min; 94°C/20 sec; 53°C/20 sec; 72°C/50 sec; 72°C/7 min | 36 |
| D1 | 28S-655R | 94°C/5 min; 94°C/0.5 min; 47°C/0.5 min; 72°C/2 min; 72°C/7 min | 36 |
| 28S-477F | D3i | 94°C/5 min; 94°C/0.5 min; 47°C/0.5 min; 72°C/2 min; 72°C/7 min | 36 |
| 18Sfw | 18Srev | 94°C/3 min; 94°C/0.5 min; 54°C/0.5 min; 72°C/1.5 min; 72°C/10 min | 35 |
| 18Sfw | 18S-1220R | 94°C/5 min; 94°C/0.5 min; 53°C/0.5 min; 72°C/1.5 min; 72°C/10 min | 35 |
| 18S-977F | 18Srev | 94°C/5 min; 94°C/0.5 min; 53°C/0.5 min; 72°C/1.5 min; 72°C/10 min | 35 |
| 18Sfw | 18S-577R | 94°C/3 min; 94°C/0.5 min; 55°C/0.5 min; 72°C/2 min; 72°C/7 min | 35 |
| 18S-397F | 18S-1220R | 94°C/3 min; 94°C/1 min; 55°C/1 min; 72°C/1 min; 72°C/10 min | 35 |
| 18S-977F | 18S-1678R | 94°C/3 min; 94°C/0.5 min; 55°C/0.5 min; 72°C/2 min; 72°C/7 min | 35 |
| 18S-1548F | 18Srev | 94°C/3 min; 94°C/0.5 min; 48°C/0.5 min; 72°C/2 min; 72°C/7 min | 36 |
| ITS3 | ITS28 | 94°C/5 min; 94°C/1 min; 48°C/1 min; 72°C/1 min; 72°C/5 min | 31 |
| CD806F | CD1231R | 94°C/3 min; 94°C/0.5 min; 60/55°C/0.5 min; 72°C/2 min; 72°C/4 min | 9/30 |
| CD806F | CD1098R | 94°C/3 min; 94°C/0.5 min; 55°C/0.5 min; 72°C/1.5 min; 72°C/10 min | 37 |
| CARWL1 | CARWL2 | 94°C/3 min; 94°C/45 sec; 58°C/45 sec; 72°C/1 min; 72°C/10 min | 35 |
| CARPEK1 | CARPEK3 | 94°C/3 min; 94°C/0.5 min; 55°C/1 min; 72°C/1 min; 72°C/10 min | 38 |
| gwenck1 | carck2 | 94°C/3 min; 94°C/0.5 min; 60°C/0.5 min; 72°C/1 min; 72°C/10 min | 35 |

**Table S6:** Partition schemes selected by PartitionFinder.

|  | Partition scheme (p=codon position) |
| --- | --- |
| BEAST | 18s;  28s;  its2;  cad-p3, co1-p1, wing-p2;  co1-p2;  co1-p3;  nd5-p1;  nd5-p2;  nd5-p3;  cad-p2, pepck-p2, pepck-p3, wing-p1;  cad-p1, pepck-p1, wingless-p3;  huwe1 |
| MrBayes | SYM+I+G: 18s;  GTR+I+G: 28s;  GTR+I+G: its2;  GTR+I+G: cad-p3, co1-p1, pepck-p3;  GTR+I+G: co1-p2;  GTR+I+G: co1-p3;  HKY+I+G: nd5-p1, nd5-p2;  GTR+G: nd5-p3;  GTR+I+G: cad-p2, pepck-p2, wingless-p1, wingless-p2;  SYM+I+G: cad-p1, pepck-p1, wingless-p3;  K80+I+G: huwe1 |
| RAxML | 18s;  28s;  its2;  co1-p1;  co1-p2;  co1-p3;  nd5-p1;  nd5-p2;  nd5-p3;  wingless-p1, cad-p2;  wingless-p2, pepck-p2, pepck-p3, cad-p3;  wingless-p3, pepck-p1, cad-p1;  huwe1 |

**Table S7:** List of *Carabus* and outgroup species used for the ancestral habitat reconstruction with information on the species specifically preferred temperature resp. elevational zones.

|  | Temp. resp. elevational zone(s) | Data source |
| --- | --- | --- |
| *Carabus (Acoptolabrus) gehinii* Fairmaire | B | [1, 2] |
| *Carabus (Apotomopterus) sauteri yunkaicus* Deuve | AB | [1, 2] |
| *Carabus (Apotomopterus) torquatus torquatus* Cavazzuti | B | [1, 2] |
| *Carabus (Archicarabus) nemoralis* Müller | B | [3] |
| *Carabus (Aulonocarabus) canaliculatus* *careniger* Chaudoir | BC | [1, 2] |
| *Carabus (Calocarabus) przewalskii elnarae* Cavazzuti | CD | [1, 2] |
| *Carabus (Carabus) arvensis* Herbst | BC | [3] |
| *Carabus (Carabus) granulatus* L. | ABC | [3] |
| *Carabus (Carabus) menetriesi* Faldermann | BC | [3] |
| *Carabus (Carpathophilus) linnaei* Panzer | BC | [3] |
| *Carabus (Chaetocarabus) intricatus intricatus* L. | AB | [3] |
| *Carabus (Chaetocarabus) intricatus krueperi* Reitter | AB | [3] |
| *Carabus (Chrysocarabus) auronitens auronitens* Fabricius | BC | [3] |
| *Carabus (Chrysocarabus) auronitens festivus* Dejean | BC | [3] |
| *Carabus (Chrysocarabus) hispanus* Fabricius | BC | [1, 3] |
| *Carabus (Cratocephalus) solskyi* Ballion | BC | [1, 4] |
| *Carabus (Ctenocarabus) melancholicus* Fabricius | B | [3] |
| *Carabus (Cupreocarabus) huangi* Deuve | CD | [1, 2] |
| *Carabus (Cytilocarabus) cribratus* Quensel | BCD | [1, 5, 6] |
| *Carabus (Damaster) elysii pulcher* Kleinfeld | AB | [1, 2] |
| *Carabus (Damaster)* *formosus* *grumorum* Semenov | AB | [1, 2] |
| *Carabus (Eurycarabus) famini* Dejean | AB | [3] |
| *Carabus (Eurycarabus) genei* Gené | AB | [3] |
| *Carabus (Hemicarabus) nitens* L. | BC | [3] |
| *Carabus (Hypsocarabus) laotse* Breuning | CD | [1, 2] |
| *Carabus (Hypsocarabus) mikhaili loustei* Deuve & Mourzine | BC | [1, 2] |
| *Carabus (Imaibius) cavifrons* Mandl | BC | own unpubl. data |
| *Carabus (Imaibius) nouristani* Ledoux | BC | [1] |
| *Carabus (Imaibius) olafi* Deuve & Schmidt | A | own unpubl. data |
| *Carabus (Isiocarabus) miaorum* Lassalle & Prunier | AB | [1, 2] |
| *Carabus (Lamprostus) calleyi* Fischer | AB | [1, 5, 7] |
| *Carabus (Leptocarabus) seishinensis seunglaki* Kwon & Lee | B | [1, 2] |
| *Carabus (Leptocarabus) semiopacus* Reitter | B | [1] |
| *Carabus (Leptocarabus) yokoae* Deuve | BC | [1, 2] |
| *Carabus (Limnocarabus) clatratus* L. | AB | [3] |
| *Carabus (Macrothorax) morbillosus* Fabricius | A | [3] |
| *Carabus (Macrothorax) rugosus* Fabricius | A | [3] |
| *Carabus (Meganebrius) alanstivelli* Morvan | CD | own unpubl. data |
| *Carabus (Meganebrius) arunensis* Heinertz | A | own unpubl. data |
| *Carabus (Meganebrius) deliae deliae* Morvan | BCD | own unpubl. data |
| *Carabus (Meganebrius) deliae deliae* Morvan | BCD | own unpubl. data |
| *Carabus (Meganebrius) deliae deliae* Morvan | BCD | own unpubl. data |
| *Carabus (Meganebrius) deliae deliae* Morvan | BCD | own unpubl. data |
| *Carabus (Meganebrius) deliae deliae* Morvan | BCD | own unpubl. data |
| *Carabus (Meganebrius) deliae morvani* Lassalle | BC | own unpubl. data |
| *Carabus (Meganebrius) deliae morvani* Lassalle | BC | own unpubl. data |
| *Carabus (Meganebrius) epsteini annapurnae* Deuve & Schmidt | DE | own unpubl. data |
| *Carabus (Meganebrius) epsteini epsteini* Heinertz | DE | own unpubl. data |
| *Carabus (Meganebrius) epsteini yetiphilus* Deuve & Schmidt | DE | own unpubl. data |
| *Carabus (Meganebrius) everesti everesti* Andrewes | BCD | own unpubl. data |
| *Carabus (Meganebrius) everesti kleinfeldi* Korell | BCD | own unpubl. data |
| *Carabus (Meganebrius) franzi* Mandl | AB | own unpubl. data |
| *Carabus (Meganebrius) granulatocostatus* Mandl | AB | own unpubl. data |
| *Carabus (Meganebrius) indicus* Fairmaire | AB | own unpubl. data |
| *Carabus (Meganebrius) kadoudali* Morvan | B | own unpubl. data |
| *Carabus (Meganebrius) koganae* Colas | BCD | own unpubl. data |
| *Carabus (Meganebrius) lebretae densestriolatus* Mandl | AB | own unpubl. data |
| *Carabus (Meganebrius) lebretae lebretae* Colas | ABC | own unpubl. data |
| *Carabus (Meganebrius) montreuili* Deuve | BC | own unpubl. data |
| *Carabus (Meganebrius) montreuili* Deuve | BC | own unpubl. data |
| *Carabus (Meganebrius) pseudoharmandi* Mandl | AB | own unpubl. data |
| *Carabus (Meganebrius) queinneci* Deuve | CD | own unpubl. data |
| *Carabus (Meganebrius) quinlani brittoni* Mandl | ABC | own unpubl. data |
| *Carabus (Meganebrius) quinlani himalchuliensis* Lassalle | ABC | own unpubl. data |
| *Carabus (Meganebrius) quinlani quinlani* Mandl | ABC | own unpubl. data |
| *Carabus (Meganebrius) quinlani smetanai* Häckel & Brezina | AB | own unpubl. data |
| *Carabus (Meganebrius) salpansis* Deuve | BC | own unpubl. data |
| *Carabus (Meganebrius) santostamangi santostamangi* Deuve & Schmidt | BC | own unpubl. data |
| *Carabus (Meganebrius) santostamangi thanglaensis* Deuve & Schmidt | CD | own unpubl. data |
| *Carabus (Meganebrius) scheibei* Eidam | BC | own unpubl. data |
| *Carabus (Meganebrius)* sp.n. near *granulatocostatus* | B | own unpubl. data |
| *Carabus (Meganebrius) tamang gurkhaorum* Lassalle | AB | own unpubl. data |
| *Carabus (Meganebrius) tamang probsti* Deuve | B | own unpubl. data |
| *Carabus (Meganebrius) tamang tamang* Deuve | ABC | own unpubl. data |
| *Carabus (Meganebrius) thudamensis* Deuve & Schmidt | C | own unpubl. data |
| *Carabus (Meganebrius) tuberculipennis ganjalaensis* Deuve | DE | own unpubl. data |
| *Carabus (Meganebrius) tuberculipennis* ssp.n. | DE | own unpubl. data |
| *Carabus (Meganebrius) tuberculipennis tuberculipennis* Mandl | DE | own unpubl. data |
| *Carabus (Meganebrius) wallichi* Hope | AB | own unpubl. data |
| *Carabus (Megodontus) germarii* Sturm | BC | [3] |
| *Carabus (Megodontus) violaceus dryas* Gistel | BC | [3] |
| *Carabus (Megodontus) violaceus purpurascens* Fabricius | BC | [3] |
| *Carabus (Megodontus) violaceus violaceus* L. | BC | [3] |
| *Carabus (Mesocarabus) dufourii* Dejean | AB | [3] |
| *Carabus (Mesocarabus) lusitanicus lusitanicus* Fabricius | AB | [3] |
| *Carabus (Mesocarabus) macrocephalus* Dejean | BC | [3] |
| *Carabus (Mesocarabus) problematicus andorranus* Lapouge | CD | [3] |
| *Carabus (Mesocarabus) problematicus problematicus* Herbst | BCD | [3] |
| *Carabus (Mesocarabus) riffensis* Fairmaire | A | [2] |
| *Carabus (Mimocarabus) maurus* (Adams) | B | [1, 5, 6] |
| *Carabus (Neoplesius) borodini* Heinz | DE | own unpubl. data |
| *Carabus (Neoplesius) danae* Kaláb | DE | own unpubl. data |
| *Carabus (Neoplesius) nanschanicus nocticolor* Deuve & Kaláb | CD | [1, 2] |
| *Carabus (Neoplesius) paulusi kocmani* Kaláb | DE | own unpubl. data |
| *Carabus (Neoplesius) paulusi paulusi* Kaláb | DE | own unpubl. data |
| *Carabus (Neoplesius) wagae folwarzcnyi* Deuve | DE | own unpubl. data |
| *Carabus (Neoplesius) wagae glasunowi* Heinz | DE | own unpubl. data |
| *Carabus (Neoplesius) wagae glinkai* Heinz | DE | own unpubl. data |
| *Carabus (Neoplesius) wagae mustangensis* Deuve | DE | own unpubl. data |
| *Carabus (Neoplesius) wagae nyalamensis* Deuve | DE | own unpubl. data |
| *Carabus (Neoplesius) wagae tanguticus* Semenov | DE | [2], own unpubl. data |
| *Carabus (Neoplesius) wagae wagae* Fairmaire | DE | [2], own unpubl. data |
| *Carabus (Nesaeocarabus) abbreviatus* Brullé | B | [3] |
| *Carabus (Ophiocarabus) latiballioni* Deuve | BCD | [1, 4] |
| *Carabus (Ophiocarabus) praecox* Semenov | BCD | [1, 4] |
| *Carabus (Ophiocarabus) tekesensis* Deuve & Tian | BCD | [1, 4] |
| *Carabus (Orinocarabus) alpestris dolomitanus* Mandl | DE | [3] |
| *Carabus (Orinocarabus) baudii* Kraatz | DE | [3] |
| *Carabus (Orinocarabus) concolor* Fabricius | DE | [3] |
| *Carabus (Orinocarabus) heteromorphus* Daniel | DE | [3] |
| *Carabus (Orinocarabus) sylvestris* Panzer | BCD | [3] |
| *Carabus (Pachystus) glabratus* Paykull | BCD | [3] |
| *Carabus (Pachystus) hortensis* L. | B | [3] |
| *Carabus (Pachystus) preslii neumeyeri* Schaum | AB | [3] |
| *Carabus (Pagocarabus) crassesculptus* Kraatz | BCD | [1, 2] |
| *Carabus (Piocarabus) reitterianus* Breuning | B | [1, 2] |
| *Carabus (Piocarabus) titanus* Breuning | B | [1, 2] |
| *Carabus (Platycarabus) creutzeri* Fabricius | BCD | [3] |
| *Carabus (Platycarabus) depressus* Bonelli | BCD | [3] |
| *Carabus (Platycarabus) irregularis* Fabricius | BC | [3] |
| *Carabus (Procrustes) coriaceus* L. | AB | [3] |
| *Carabus (Pseudocranion) gansuensis* Semenov | BCD | [1, 2] |
| *Carabus (Pseudocranion) sackeni remondianus* Deuve | CD | [1, 2] |
| *Carabus (Pseudocranion) tibetanophilus yak* Deuve | CD | [1, 2] |
| *Carabus (Rhigocarabus) buddaicus gansuicus* Deuve | CD | [1, 2] |
| *Carabus (Rhigocarabus) cateniger* Morawitz | CD | [1, 2] |
| *Carabus (Rhigocarabus) gracilicollis shatanensis* Deuve & Tian | C | [1, 2] |
| *Carabus (Rhigocarabus) ladygini* Semenov | DE | [1, 2], own unpubl. data |
| *Carabus (Rhigocarabus) morawitzianus* Semenov | DE | [1, 2], own unpubl. data |
| *Carabus (Rhigocarabus) roborowskii* Semenov | DE | [1, 2], own unpubl. data |
| *Carabus (Rhigocarabus) xiei abaxianensis* Deuve & Tian | CD | [1, 2] |
| *Carabus (Sphodristocarabus) armeniacus* Mannerheim | BCD | [1, 5, 7] |
| *Carabus (Tachypus) auratus* L. | BC | [3] |
| *Carabus (Tomocarabus) convexus convexus* Fabricius | AB | [3] |
| *Carabus (Tomocarabus) convexus hornschuchi* Hoppe | AB | [3] |
| *Carabus (Tomocarabus) fraterculus jirisanensis* Ishikawa & Kim | B | [1, 2] |
| *Carabus (Tomocarabus) rumelicus syriensis* Breuning | B | [1, 8] |
| *Carabus (Ulocarabus) theanus* Reitter | BC | [1] |

[1] T. Deuve, Illustrated catalogue of the genus *Carabus* of the World (Pensoft, 2013), pp. 307.

[2] H. Schütze, F. Kleinfeld, Die Caraben Chinas – Systematik – alle Taxa – Bibliographie – Lexikon aller literaturbekannten Fundorte. (Delta Druck Peks, 2007), pp. 332.

[3] H. Turin, L Penev, A. Casale, The genus *Carabus* in Europe. A Synthesis, (Pensoft, 2013), pp. 327-425.

[4] H. Schütze, F. Kleinfeld, Carabusformen Sibiriens und Zentral-Asiens – Taxa – Bibliographie – mit einem besonders ausführlichen Fundorteverzeichnis. (Kleinfeld, 1997), pp. 198.

[5] H. Schütze, F. Kleinfeld, Die Caraben Kaukasiens mit einem besonders ausführlichen Fundorteverzeichnis – Taxa – Systematik – Bibliographie. (Kleinfeld, 2001), pp. 178.

[6] P. Cavazzuti, Fauna des Carabinae de Turquie – I. Collection Systématique 13 (Magellanes, 2006), pp. 155.

[7] P. Cavazzuti, Fauna dei Carabini di Turchia – II. Collection Systématique 25 (Magellanes, (2014), pp. 205.

[8] F. Kleinfeld, I. Rapuzzi, Die *Carabus*-Fauna im Nahen Osten. (Kleinfeld, 2016), pp. 108.

[9] D. Maguerre, *Calosoma*, *Carabus* *et Cychrus* de France (Coleoptera, Carabidae). Collection Systématique 28, (Magellanes, 2016), pp. 395.

**Table S8:** List of supra-specific taxa with information about taxon specific species numbers, number of species used for the analyses, and species group specific distributions. Himalayan species groups are highlighted (grey).

| supra-specific taxon | No of species/ supra-specific taxon | No of species used for molecular analyses | distribution |
| --- | --- | --- | --- |
| Cychrini | 213 | 6 | Holarctic |
| Carabini | 1098 | 109 | Global (extra-antarctic) |
| *Calosoma* Weber | 128 | 4 | Global (extra-polar) |
| *Carabus* L. | 970 | 105 | Holarctic |
| subgenus *Acoptolabrus* Morawitz | 9 | 1 | eastern Palearctic |
| subgenus *Apotomopterus* Hope | 139 | 2 | eastern Palearctic |
| subgenus *Archicarabus* Seidlitz | 10 | 1 | western Palearctic |
| subgenus *Aulonocarabus* Reitter | 14 | 1 | eastern Palearctic |
| subgenus *Calocarabus* Séménov | 24 | 1 | Northeast Tibet endemic |
| subgenus *Carabus* s. str. | 30 | 3 | Holarctic |
| subgenus *Carpathophilus* Reitter | 1 | 1 | Central European |
| subgenus *Chaetocarabus* Thomson | 2 | 1 | European |
| subgenus *Chrysocarabus* Thomson | 7 | 2 | European |
| subgenus *Cratocephalus* Kirsch | 4 | 1 | Central Asiatic |
| subgenus *Ctenocarabus* Thomson | 2 | 1 | atlanto-mediterranean |
| subgenus *Cupreocarabus* Deuve | 31 | 1 | East Tibet endemic |
| subgenus *Cytilocarabus* Reitter | 4 | 1 | Northeast Tibet endemic |
| subgenus *Damaster* Kollar | 19 | 2 | eastern Palearctic |
| subgenus *Eurycarabus* Géhin | 2 | 2 | atlanto-mediterranean |
| subgenus *Hemicarabus* Géhin | 4 | 1 | Holarctic |
| subgenus *Hypsocarabus* Séménov | 8 | 2 | Northeast Tibet endemic |
| subgenus *Imaibius* Séménov | 25 | 3 | West Himalaya endemic |
| subgenus *Isiocarabus* Reitter | 13 | 1 | eastern Palearctic |
| subgenus *Lamprostus* Motschulsky | 16 | 1 | ponto-mediterranean |
| subgenus *Leptocarabus* Géhin | 11 | 3 | eastern Palearctic |
| subgenus *Limnocarabus* Géhin | 2 | 1 | Palearctic |
| subgenus *Macrothorax* Desmarest | 5 | 2 | atlanto-mediterranean |
| subgenus *Meganebrius* Kraatz | 22 | 22 | Central Himalaya endemic |
| *Carabus scheibei* group | 2 | 2 | West Himalaya endemic |
| subgenus *Megodontus* Solier | 21 | 2 | Palearctic |
| subgenus *Mesocarabus* Thomson | 5 | 5 | European |
| subgenus *Mimocarabus* Thomson | 5 | 1 | ponto-mediterranean |
| subgenus *Neoplesius* Reitter | 24 | 5 | Tibet endemic |
| subgenus *Nesaeocarabus* Bedel | 4 | 1 | Canarian |
| subgenus *Ophiocarabus* Reitter | 16 | 3 | Central Asiatic |
| subgenus *Orinocarabus* Kraatz | 9 | 5 | Central European |
| subgenus *Pachystus* Motschulsky | 8 | 3 | western Palearctic |
| subgenus *Pagocarabus* Morawitz | 7 | 1 | East Tibet endemic |
| subgenus *Piocarabus* Reitter | 9 | 2 | eastern Palearctic |
| subgenus *Platycarabus* Morawitz | 5 | 3 | Central European |
| subgenus *Procrustes* Bonelli | 8 | 1 | ponto-mediterranean |
| subgenus *Pseudocranion* Reitter | 20 | 3 | Northeast Tibet endemic |
| subgenus *Rhigocarabus* Reitter | 45 | 7 | East Tibet endemic |
| subgenus *Sphodristocarabus* Géhin | 15 | 1 | ponto-mediterranean |
| subgenus *Tachypus* Weber | 3 | 1 | western Palearctic |
| subgenus *Tomocarabus* Reitter | 20 | 3 | Holarctic |
| subgenus *Ulocarabus* Reitter | 2 | 1 | Middle Asiatic |
